# Supplementary material for: Impact of COVID-19 pandemic social restriction measures on people with rheumatic and musculoskeletal diseases in the UK: a mixed-methods study
Source: BMJ Open. 2021 Jun 2;11(6):e048772. doi: 10.1136/bmjopen-2021-048772 (PMC8182755; doi:10.1136/bmjopen-2021-048772)
Supplement: Supplementary data [file bmjopen-2021-048772supp001.pdf]

Supplementary File 1: Online survey (baseline)

The impact of COVID-19 self-isolation measures on people with musculoskeletal diseases

Questionnaire

Version 0.1 - dated: 19 April 2020

Information to help you decide if you want to take part in this research

You are being invited to take part in a research project so we can understand how you are managing self-isolation during the coronavirus pandemic (Covid-19). We are particular interested in your joint pain, wellbeing and social contacts. Please ensure you have read and understood this information before continuing.

What is this project about?

This study aims to identify how the Covid-19 pandemic is impacting on the health and wellbeing of people with bone, joint or muscle pain (musculoskeletal disease). It aims to better understand how the current Covid-19 self-isolation is affecting people, their experiences of isolating and how they are managing their joint pain during this time. It will tell us about the effects of social isolation measures and changes in health services on people's physical health and wellbeing. The findings will be used to help understand who is at greatest risk of poor health and wellbeing during self-isolation so we can better support them during this outbreak.

Why have I been asked?

You have been sent this survey or have accessed it via social media. The project is entirely voluntary. You do not have to be in isolation to take part.

What will taking part involve?

You will be asked to complete a 15-minute survey online. Then every 2 weeks for the next 12 weeks, we will ask you to complete the same survey again. The whole study will last for about 12 weeks while these social isolation measures are in place.

What will you ask and what will happen to the information I give you?

You will be asked questions about yourself, your current joint pain, your medication and health care requirements for joint pain, and some questions on your physical health and wellbeing. We will also ask you whether you are isolating, some questions on social isolation, loneliness and resilience to this change in lifestyle, and some questions on your arthritis symptoms. Some of these questions are considered sensitive data, such as questions on your wellbeing. No information provided will be passed to any third parties. Your data will then be analysed by researchers from UEA and will be published in scientific papers and used to inform advice given during Covid-19. Nobody will be able to identify you from any materials we publish or present from this study.

You will only be identified to the research team members by your email address. We need this so that we can send you the follow-up fortnightly survey links for the next 12 weeks. We will

not use your email address for any other purpose. We will not share this email address with any other people or organisations.

This study has received approval from the University of East Anglia's Faculty of Medicine and Health Science's Research Ethics Committee.

How long will my data be stored for?

Your data will only be used for this study and not shared. Your data will be stored for 10 years by UEA after the end of the research. At this point the data will be reviewed, and if they are still deemed to be of public interest, they may be retained for longer.

How do I find out the results?

Researchers at UEA will provide a summary of the findings online through the UEA website. We will provide a hyperlink to all participants (via their email address) of this once the data has been analysed and reported.

Concerns

If you have any concerns about the study, you can contact the UEA study team at 01603 597205 or email noar@uea.ac.uk. If you feel your concerns have not been handled satisfactorily, you can contact the Dean of the Norwich Medical School (Professor William Fraser) at: W.Fraser@uea.ac.uk

Consent

By taking part, you are agreeing that you have read and understood the information above about the study. If you have any questions or concerns please contact 01603 597205 before agreeing to take part and completing the survey.

I understand that:

- My participation is completely voluntary.
- The data gathered in this study will be stored securely and it will not be possible to identify me in any outputs from this research.
- Only UEA research staff, will have access to the data.

I consent by ticking this box: ☐

Survey

Date of birth ☐☐ / ☐☐ / ☐☐☐☐

Date completed ☐☐ / ☐☐ / ☐☐☐☐

Email Address (to send the follow-up surveys) ☐

Female ☐

Male ☐

Age (years) ☐

What is was/your occupation:____

Are you currently:

a. Working now ☐
b. Unemployed ☐
c. Off sick ☐
d. Retired ☐
e. Not working – health grounds ☐
f. Never worked ☐
g. Homekeeper/parent/carer ☐
h. Student ☐
i. Working reduced hours ☐
j. Working, but there has been a change in duties ☐
k. Maternity/Paternity leave ☐

	YES	NO
**Are you currently off work because of coronavirus self-isolation?**		
**Are you currently working from home because of coronavirus self-isolation?**		

Which category best describes your ethnic group or background. (Tick only one box)

White	
Black, Caribbean	
Black, African	
Any other Black background	
Indian	
Pakistani	
Bangladeshi	
Any other Asian background	
Chinese	
White and Black Caribbean	
White and Black African	
White and Asian	
Any other mixed background	
Any other ethnic group	

Your Bone, Joint and Muscle Disease

What is your musculoskeletal disease diagnosis? (Please tick all relevant boxes)

Rheumatoid Arthritis (RA)	
Osteoarthritis (OA)	
Fibromyalgia	
Ankylosing Spondylitis	
Psoriatic Arthritis (PsA)	
Inflammatory Polyarthritis (IP)	
Mechanical Low Back Pain	
Other (please specify) ______________________________	
Don't Know	

Where do you feel your pain?

	Left	Right
Hip		
Knee		
Ankle		
Foot		
Shoulder		
Elbow		
Wrist		
Hand		
Neck		
Back (mid or low back)		

General Health Questions

1. Have you had any of the following conditions?

	YES	NO
Heart condition (such as heart failure or past heart attack)		
High blood pressure		
Stroke or TIA (mini stroke/transient visual loss)		
Breathing condition (such as asthma, emphysema, COPD)		
Diabetes		
Stomach Ulcer		
Liver problems		
Kidney problems		
Cancer (except skin cancer)		
Depression or Anxiety		
Glaucoma		
Dementia		
Epilepsy		

Changes since self-isolation

Are you self-isolated for COVID-19 (i.e. not leaving home for activities other than shopping, exercise once daily or essential tasks)?

❑ Yes ❑ No

Have you had difficulty accessing medications?

❑ Yes ❑ No

Have you needed someone to help to access your medications?

❑ Yes ❑ No

Have you had to change your medications since the COVID-19 pandemic started?

❑ Yes ❑ No

Have you needed to seek advice from a health professional on your condition?

❑ Yes ❑ No

Has your normal level of physical activity changed since the COVID self isolation started?

❑ Decreased ❑ Stayed same ❑ Increased

How has your joint pain been overall since the COVID self isolation started?

❑ Decreased ❑ Stayed same ❑ Increased

How have your levels of energy been since the COVID self isolation started?

❑ Decreased ❑ Stayed same ❑ Increased

BMJ Publishing Group Limited (BMJ) disclaims all liability and responsibility arising from any reliance placed on this supplemental material which has been supplied by the author(s)

Clinical Health Assessment Questionnaire (CLINHAQ)

We are interested in learning how your illness affects your ability to function in daily life.

Please tick the response which best describes your usual abilities OVER THE PAST WEEK:

	Without any difficulty	With some difficulty	With much difficulty	Unable to do
DRESSING & GROOMING: Are you able to:				
Dress yourself, including tying shoelaces & doing buttons?	_______	_______	_______	_______
Shampoo your hair?	_______	_______	_______	_______
RISING: Are you able to:				
Stand up from an armless straight chair?	_______	_______	_______	_______
Get in and out of bed?	_______	_______	_______	_______
EATING: Are you able to:				
Cut your meat?	_______	_______	_______	_______
Lift a full cup or glass to your mouth?	_______	_______	_______	_______
Open a new carton of milk (or soap powder)?	_______	_______	_______	_______
WALKING: Are you able to:				
Walk outdoors on flat ground?	_______	_______	_______	_______
Climb up five steps?	_______	_______	_______	_______

Please tick any AIDS or DEVICES that you usually use for any of these activities:

______ Cane (W) ______ Walking frame (W) ______ Built up or special utensils (E)

______ Crutches (W) ______ Wheelchair (W) ______ Special or built up chair (A)

______ Devices used for dressing (button hook, zipper pull, long handled shoe horn) (D)

______ Other, specify ____

Please tick any category for which you usually need HELP FROM ANOTHER PERSON:

________ Dressing & grooming __________ Eating

________ Rising __________ Walking

We are also interested in learning whether or not you are affected by pain because of your illness.

How much pain have you had because of your arthritis IN THE PAST WEEK?

Place a mark on the line to indicate the severity of the pain

No pain Severe pain

0 10 20 30 40 50 60 70 80 90 100

How much trouble have you had with your stomach (ie nausea, heartburn, bloating, pain, etc)

IN THE PAST WEEK? Place a mark on the line below to indicate the severity of the problem:

No stomach problem Severe stomach problem

0 10 20 30 40 50 60 70 80 90 100

Please tick the response which best describes your usual abilities OVER THE PAST WEEK:

	Without any difficulty	With some difficulty	With much difficulty	Unable to do
HYGIENE: Are you able to:				
Wash and dry your entire body?	_______	_______	_______	_______
Take a bath?	_______	_______	_______	_______
Get on and off the toilet?	_______	_______	_______	_______
REACH: Are you able to:				
Reach and get down a 5lb object (eg a bag of potatoes) from just above your head?	_______	_______	_______	_______
Bend down to pick up clothing from the floor?	_______	_______	_______	_______
GRIP: Are you able to:				
Open car doors?	_______	_______	_______	_______
Open jars which have been previously opened?	_______	_______	_______	_______
Turn taps on and off?	_______	_______	_______	_______
ACTIVITIES: Are you able to:				
Run errands and shop?	_______	_______	_______	_______
Get in and out of a car?	_______	_______	_______	_______
Do chores (vacuuming, housework or light gardening)?	_______	_______	_______	_______

Please tick any AIDS OR DEVICES that you usually use for any of these activities:

________ Bath rail (H) _______ Bath seat (H)

________ Raised toilet seat (H) _______ Jar opener (for jars previously opened) (G)

________ Long-handled appliances for reach (R) _______ Other (specify) ____________________

Please tick any categories for which you usually need HELP FROM ANOTHER PERSON:

________ Hygiene _______ Gripping and opening things

_______ Reach _______ Errands and chores

In general, would you say that your HEALTH IS:

_______ Excellent _______ Good _______ Fair _______Poor

Consider ALL THE WAYS THAT YOUR ARTHRITIS AFFECTS YOU. RATE HOW YOU ARE DOING on the following scale by placing a mark on the line below:

Very well Very poorly

0 10 20 30 40 50 60 70 80 90 100

We are interested in knowing about any problems that you may have been having with fatigue.

How much of a problem has fatigue or tiredness been for you IN THE PAST WEEK? Place a mark on the line below:

Fatigue is no problem Fatigue is a major problem

0 10 20 30 40 50 60 70 80 90 100

How much of a problem has sleep (ie resting at night) been for you IN THE PAST WEEK? <u>Place a mark on the line below:</u>

Sleep is no problem — Sleep is a major problem

0 10 20 30 40 50 60 70 80 90 100

Lubben Social Network Scale-6

The following 6 questions are about how often you are in contact with your family and friends. Please **tick** the box to best represent your answer for each question.

	None	1	2	3 or 4	5 to 8	9 or more
Family – considering the people to whom you are related by birth, marriage, adoption etc…						
How many relatives do you see or hear from at least once a month?						
How many relatives do you feel at ease with that you can talk about private matters?						
How many relatives do you feel close to such that you could call on them for help?						
Considering all of your friends, including those who live in your neighbourhood						
How many of your friends do you see or hear from at least once a month?						
How many friends do you feel at ease with that you can talk about private matters?						
How many friends do you feel close to such that you could call on them for help?						

University of California, Los Angeles (UCLA) three-item loneliness scale

The following 3 questions are about how often you feel lonely. Please **tick** the box to best represent your answer for each question.

	Hardly Ever	Some of the Time	Often
How often do you feel that you lack companionship?			
How often do you feel left out?			
How often do you feel isolated from others?			

6-item Brief Resilience Scale

The following 6 statement relate to how 'resilient' you feel. Please respond to each item by marking one box per row.

	Strongly Disagree	Disagree	Neutral	Agree	Strongly Agree

I tend to bounce back quickly after hard times					
I have a hard time making it through stressful events					
It does not take me long to recover from a stressful events					
It is hard for me to snap back when something wrong happens					
I usually come through difficult times with little trouble					
I tend to take a long time to get over setbacks in my life					

Revised Life Orientation Test (LOT-R)

Please answer the following questions about yourself. Be as honest as you can throughout and try not to let your responses to one question influence your response to other questions. There are no right or wrong answers.

	Strongly Disagree	Disagree	Neutral	Agree	Strongly Agree
In uncertain times, I usually expect the best					
It’s easy for me to relax					
If something can go wrong for me, it will					
I’m always optimistic about my future					
I enjoy my friends a lot					
It’s important for me to keep busy					
I hardly ever expect things to go my way					
I don’t get upset too easily					
I rarely count on good things happening to me					
Overall, I expect more good things to happen to me than bad					

We would like to thank you for completing these questionnaires.
Your contribution is much appreciated.
If you have any questions or queries about this form, please contact:
NOAR Office Tel: (01603) 597204/5
Email: noar@uea.ac.uk

Supplementary File 2: Timeline of study activity against UK legislation and COVID policy implementation.

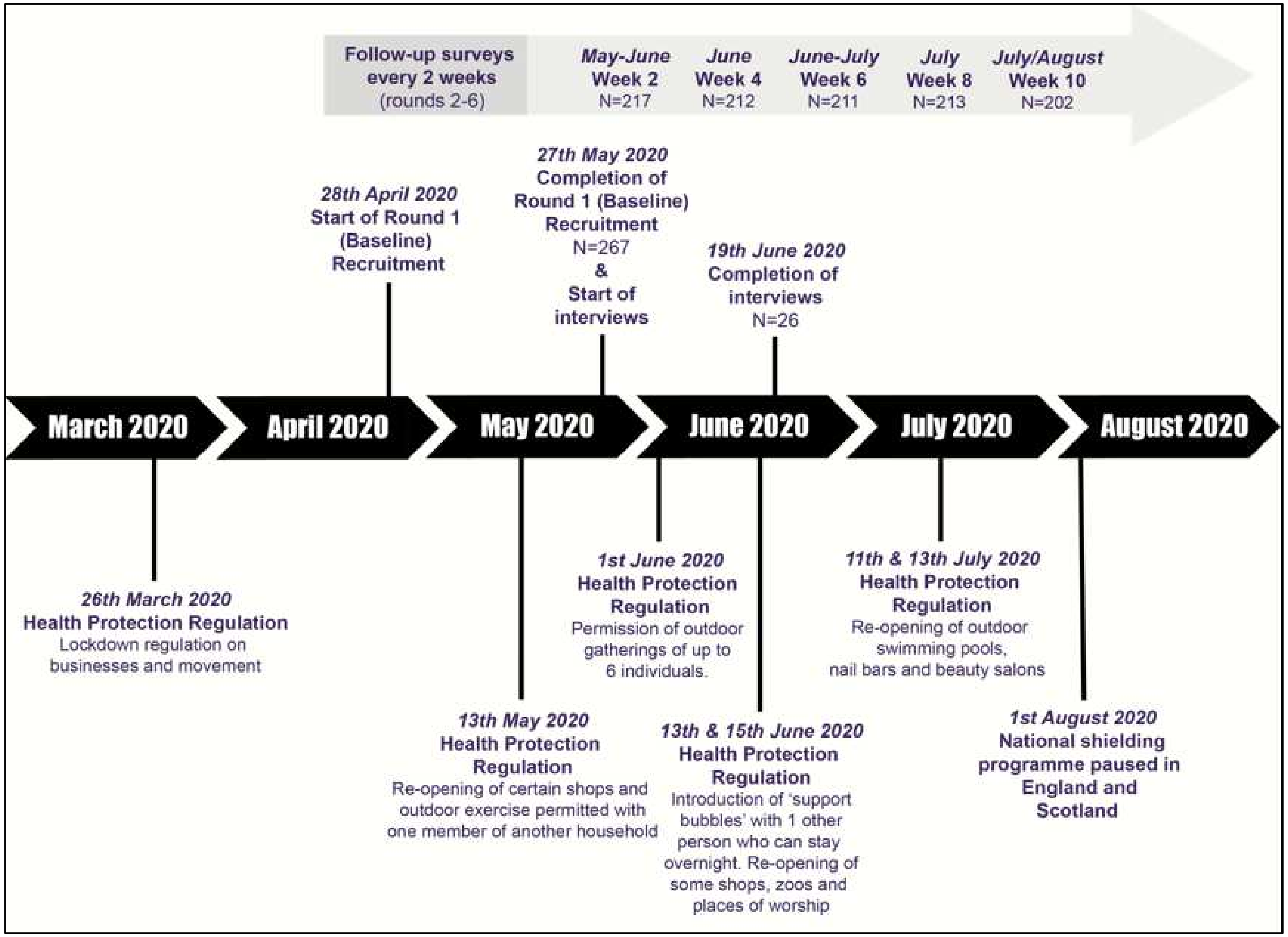

Supplementary File 3: Interview topic guide

1 **How is everyday life for you at the moment?**

Prompts:
- Getting out the house
- Keeping in touch with people
- Changes in routines
- Changes in mood
- Changes in health
- Any adaptions made

2 **How is your joint pain?**

Prompts
- Recent changes and what they think is the cause
- Fatigue
- Medication
- Exercise
- Self help
- Contact with health professionals

3 **How are you managing daily tasks?**

Prompts
- Do you usually have help shopping, cleaning
- Have you made changes to the ways you do things

3a How do you feel about these changes?

4 **How have your social activities changed?**

Prompts
- Keeping in touch with family and friends
- Activities in the community i.e. clubs, quizzes crafts, pub
- Using social media
- Accessing support groups
- New social contacts

4a How do you feel about these changes?

5 **Is there anything else you would like to tell us about your life during this period?**

Supplementary File 4: Characteristics of participants at baseline and Week 10

	Timepoint		P-value (chi-squared)
	Baseline	Week 10	
N (% of baseline)	703 (100)	490 (70)	
**Age group**			p=0.022
18-60	351 (50)	212 (43)	
60 plus	351 (50)	278 (57)	
**Gender**			p=0.804
Male	574 (82)	402 (83)	
Female	126 (18)	85 (17)	
**MSK Diagnosis**			p=0.632
RA	321 (46)	241 (48)	
IA	41 (6)	29 (6)	
PsA	65 (9)	48 (9)	
Other	269 (39)	171 (37)	

Data are frequency (%); MSK – Musculoskeletal; RA - Rheumatoid arthritis, IA - Inflammatory arthritis; PsA - Psoriatic arthritis; Other - mainly Osteoarthritis (55%) but also including any diagnosis that was not RA, IA or PsA (see **Table 1**)

Supplementary File 5: Interpretive synthesis of quantitative and qualitative findings

Survey Findings (See Tables 1-2)	Interview Findings (See Tables 3-4)
People with musculoskeletal disease reported an increase in pain and symptoms	*Attributions from symptom increase:*<br>- Changes in access to healthcare<br>- Reduced physical activity<br>- Ergonimics of working at home<br>- Reduced wellbeing<br>- Increased demands at home
Those who reported decreased physical activity were more likely to report increased symptoms	*Increase in symptoms attributed to:*<br>- Reduced routine activitiy<br>- Reduced opportunities for exercise
Increase in symptoms was significantly worse for those aged 18-60 years of age in comparison to older respondents	*Changes to daily life described by working age people included:*<br>- Disrupted daily routines<br>- Managing multiple roles at home, including childcare<br>- Unsuitable home working environment
Over the 10 weeks, greater social isolation, loneliness and reduced optimism were reported	*Feelings of low mood, boredom, social isolation or anxiety were related to:*<br>- Experience of restrictive measures<br>- Being in a clinically vulnerable group<br>- Disease symptoms<br>- A sense of being forgotten about
Half of those (53%) with increased symptoms at baseline had not consulted a healthcare professional	*Disrupted access to healthcare:*<br>- 'Holding on to concerns', wary of burdening healthcare staff, or uncertainty around service availability<br>- Reservations about telephone consultations<br>- Appointments, treatments and investigations postponed or cancelled
