# Supplementary material for: Impact of COVID-19 pandemic social restriction measures on people with rheumatic and musculoskeletal diseases in the UK: a mixed-methods study
Source: BMJ Open. 2021 Jun 2;11(6):e048772. doi: 10.1136/bmjopen-2021-048772 (PMC8182755; doi:10.1136/bmjopen-2021-048772)
Supplement: Author's manuscript [file bmjopen-2021-048772.draft_revisions.pdf]

BMJ Open is committed to open peer review. As part of this commitment we make the peer review history of every article we publish publicly available.

When an article is published we post the peer reviewers' comments and the authors' responses online. We also post the versions of the paper that were used during peer review. These are the versions that the peer review comments apply to.

The versions of the paper that follow are the versions that were submitted during the peer review process. They are not the versions of record or the final published versions. They should not be cited or distributed as the published version of this manuscript.

BMJ Open is an open access journal and the full, final, typeset and author-corrected version of record of the manuscript is available on our site with no access controls, subscription charges or pay-per-view fees (http://bmjopen.bmj.com).

If you have any questions on BMJ Open's open peer review process please email info.bmjopen@bmj.com

BMJ Open

The impact of COVID-19 social restriction measures on people with chronic musculoskeletal diseases in the UK: a mixed-methods study.

Journal:	*BMJ Open*
Manuscript ID	bmjopen-2021-048772
Article Type:	Original research
Date Submitted by the Author:	07-Jan-2021
Complete List of Authors:	Smith, Toby O.; University of Oxford , Nuffield Department of Orthopaedics, Rheumatology and Musculoskeletal Sciences; University of East Anglia,<br>Belderson, P; University of East Anglia<br>Dainty, Jack; University of East Anglia Faculty of Medicine and Health Sciences<br>Birt, Linda; University of East Anglia, School of Health Sciences<br>Durrant, K; University of East Anglia<br>Chipping, JR; University of East Anglia<br>Tsigarides, J; University of East Anglia, Rheumatology Department; Norfolk and Norwich University Hospital, Rheumatology Department<br>Yates, M; University of East Anglia, Rheumatology Department; Norfolk and Norwich University Hospital, Rheumatology Department<br>Werry, S<br>Naughton, Felix; University of East Anglia Faculty of Medicine and Health Sciences, School of Health Sciences; University of East Anglia<br>Notley, Caitlin; University of East Anglia, Norwich Medical School<br>Shepstone, Lee; University of East Anglia, Norwich Medical School<br>MacGregor, Alex; University of East Anglia, Rheumatology Department; Norfolk and Norwich University Hospital, Rheumatology Department
Keywords:	Rheumatology < INTERNAL MEDICINE, Pain management < ANAESTHETICS, COVID-19, Musculoskeletal disorders < ORTHOPAEDIC & TRAUMA SURGERY

SCHOLARONE™
Manuscripts

BMJ

I, the Submitting Author has the right to grant and does grant on behalf of all authors of the Work (as defined in the below author licence), an exclusive licence and/or a non-exclusive licence for contributions from authors who are: i) UK Crown employees; ii) where BMJ has agreed a CC-BY licence shall apply, and/or iii) in accordance with the terms applicable for US Federal Government officers or employees acting as part of their official duties; on a worldwide, perpetual, irrevocable, royalty-free basis to BMJ Publishing Group Ltd ("BMJ") its licensees and where the relevant Journal is co-owned by BMJ to the co-owners of the Journal, to publish the Work in this journal and any other BMJ products and to exploit all rights, as set out in our licence.

The Submitting Author accepts and understands that any supply made under these terms is made by BMJ to the Submitting Author unless you are acting as an employee on behalf of your employer or a postgraduate student of an affiliated institution which is paying any applicable article publishing charge ("APC") for Open Access articles. Where the Submitting Author wishes to make the Work available on an Open Access basis (and intends to pay the relevant APC), the terms of reuse of such Open Access shall be governed by a Creative Commons licence – details of these licences and which Creative Commons licence will apply to this Work are set out in our licence referred to above.

Other than as permitted in any relevant BMJ Author's Self Archiving Policies, I confirm this Work has not been accepted for publication elsewhere, is not being considered for publication elsewhere and does not duplicate material already published. I confirm all authors consent to publication of this Work and authorise the granting of this licence.

For peer review only

BMJ Open

TITLE PAGE

TITLE: The impact of COVID-19 social restriction measures on people with chronic musculoskeletal diseases in the UK: a mixed-methods study.

AUTHORS: Smith TO,[1,2] Belderson P,[1] Dainty JR,[1] Birt L,[1] Durrant K,[1] Chipping JR,[1] Tsigarides J,[1,3] Yates M,[1,3] Naughton F,[1] Werry S,[4] Notley C,[1] Shepstone L,[1] Macgregor AJ[1,3]

AFFILIATIONS

1. Faculty of Medicine and Health Sciences, University of East Anglia, Norwich, UK
2. Nuffield Department of Rheumatology, Orthopaedics and Musculoskeletal Sciences, University of Oxford, Botnar Research Centre, Windmill Road, Oxford, UK
3. Rheumatology Department, Norfolk and Norwich University Hospital, Norwich, UK
4. Patient and Public Representative, Norwich, UK

Corresponding Author: Dr Toby Smith, Nuffield Department of Rheumatology, Orthopaedics and Musculoskeletal Sciences, University of Oxford, Botnar Research Centre, Windmill Road, Oxford, UK. Email: toby.smith@ndorms.ox.ac.uk

Word Count: 4572 manuscript

For peer review only

For peer review only - http://bmjopen.bmj.com/site/about/guidelines.xhtml

ABSTRACT

OBJECTIVES: To determine the impact of COVID-19 social restriction measures on people with chronic musculoskeletal diseases and to explore how people adapted to these measures over time.

DESIGN: Mixed-methods investigation comprising a national online longitudinal survey and embedded qualitative study.

SETTING: UK online survey and interviews with community-dwelling individuals in the East of England.

PARTICIPANTS: People in the UK with chronic musculoskeletal disease, were invited to participate in an online survey. A subsection of respondents were invited to participate in the embedded qualitative study.

PRIMARY AND SECONDARY OUTCOME MEASURES: The online survey, completed fortnightly over 10 weeks from April 2020 to August 2020, investigated changes in symptoms, social isolation and loneliness, resilience and optimism. Qualitative interviews were undertaken assessing participant's perspectives on changes in symptoms, exercising, managing instrumental tasks such a shopping, medication and treatment regimens, and how they experienced changes in their social networks.

RESULTS: 708 people completed the online survey. These people frequently reported a deterioration in symptoms as a result of COVID-19 social restrictions (53% reported increase vs. 6% reported a decrease; $p<0.001$). This was significantly worse for those aged 18 to 60 years compared to older participants ($p=0.012$). The qualitative findings from 30 individuals with chronic musculoskeletal diseases suggest that the greatest change in daily life was experienced by those in employment. Although some retired people reported reduced opportunity for exercise outside their homes, they did not face the many competing demands experienced by employed people and people with children at home.

CONCLUSIONS: People with chronic musculoskeletal diseases reported a deterioration in symptoms when COVID-19 social restriction measures were enforced. This was worse for working-aged people. Consideration of this at-risk group, specifically for the promotion of physical activity, changing home-working practices and awareness of healthcare provision is important, as social restrictions continue in the UK.

Keywords: coronavirus; rheumatology; joint pain; social isolation; occupational adaptation; qualitative; survey

STRENGTHS AND LIMITATIONS OF THIS STUDY

- This is the first study to detail the impact of COVID-19 social restrictions on the lived-experiences of people with chronic musculoskeletal diseases.
- This UK longitudinal survey of 708 people included an embedded qualitative study providing explanations on the quantitative results.
- A breadth of important health domains were examined over the initial COVID-19 10-week social restriction period, including pain, social isolation and loneliness, resilience and optimism.
- We only recruited UK residents so while the principles may apply to countries experiencing social restrictions due to COVID-19, results may not be generalisable beyond the UK.

For peer review only

For peer review only - http://bmjopen.bmj.com/site/about/guidelines.xhtml

INTRODUCTION

Musculoskeletal disease is a major cause of disability and reduced quality of life worldwide.[1] People with chronic musculoskeletal diseases experience pain, joint stiffness, fatigue and muscle weakness with resultant physical disability. These symptoms are frequently managed with a combination of physical activity and medications, which for some people include immunosuppressive drugs.

In March 2020, the World Health Organization (WHO) declared the outbreak of a novel coronavirus disease SARs-CoV-2 (COVID-19) to be a pandemic. On 16th March 2020, the UK government instructed all people aged 70 years and over to self-isolate in their homes for up to four months. This was extended to all people on 23rd March for an initial three-week period. People who were taking immunosuppressant medications or with chronic medical comorbidities were instructed to maintain strict self-isolation principles (often referred to as shielding) for an extended period of at least 12 weeks (**Supplementary File 1**).

At the outset, there was concern that people with musculoskeletal diseases may be particularly vulnerable to the unintended consequences of these 'lockdown' measures. They are recognised to be at increased risk of social isolation, through poor social support,[2] the frequent presence of other medical conditions[3,4] and functional disability.[5] Social isolation itself can affect levels of physical disability, psychological distress and pain in musculoskeletal disease.[6] The loss of exercise opportunities, a key component of self-management programmes, risked increasing levels of disability and reducing mental wellbeing. COVID-19 measures also significantly impacted on routine healthcare access and NHS services.[7,8] Prior to this study, the resilience of this group to these enforced changes was unknown, as were likely long-term impacts.

This study explores the impact of COVID-19 social restriction measures on people with chronic musculoskeletal diseases and determines how people adapted to these measures over time. Our aim was to provide insight into the challenges and potential impacts of those most at risk, and to make recommendations as to how best to support people with musculoskeletal disease throughout further social restriction measures and beyond.

METHODS AND ANALYSIS

Design

Mixed-methods investigation comprising a national online longitudinal survey and embedded qualitative study. The study is reported in accordance with the STROBE statement for reporting observational studies[9] and the COREQ statement for reporting qualitative research.[10]

Survey Study

Cohort and Recruitment

We invited participants who self-reported a diagnosis of chronic (three months or longer) musculoskeletal disease (disease of the bone, joint or muscle), and who lived in the UK to participate in an online survey.

We used three approaches to recruit potential participants. Firstly, we approached patient-based organisations (Versus Arthritis, Arthritis Action, Royal Osteoporosis Society, National Rheumatoid Arthritis Society, National Ankylosing Spondylitis Society, Fibromyalgia UK, Age UK, PainConcern and

For peer review only - http://bmjopen.bmj.com/site/about/guidelines.xhtml

Scope). All agreed to disseminate an invitation to the survey via their online patient forum or as an email. Secondly, we contacted members from an inception cohort of 1,396 people with inflammatory arthritis – the Norfolk Arthritis Register (NOAR).[11] Finally, we used research team members' Twitter accounts to publicise the study.

Data Collection

An online survey administered through the Qualtrics platform (Qualitrics XM, https://www.qualtrics.com/uk/) was provided to all participants. Participant consent was obtained through the platform before proceeding to the study survey. The survey collected information on:

- age, gender, ethnicity, duration of symptoms, medical co-morbidities.
- clinical disease activity.
- changes in medication use, access to healthcare support, physical activity, disease symptoms since COVID-19 lock-down.
- Clinical Health Assessment Questionnaire (CLINHAQ)[12] to assess disability and symptom status including: pain, fatigue, sleep, anxiety.
- the Lubben Social Network Scale 6[13] – short assessment of social isolation.
- University of California, Los Angeles (UCLA) three-item loneliness scale.[14]
- 6-item Brief Resilience Scale (BRS-6).[15]
- revised Life Orientation Test (LOT-R).[16]

We opened Round 1 (baseline) recruitment from 28th April 2020 to 27th May 2020. We sent tweets weekly during Round 1 recruitment period. For those who completed Round 1, we sent the same survey at two-week intervals to investigate how symptoms, social isolation, loneliness, resilience and optimism changed over time (Rounds 2 to 6). We sent a reminder email to participants who missed a subsequent round. The final survey was completed on 20th August 2020.

Data Analysis

The analysis first addressed the perceived changes in symptoms since the start of lockdown in response to the question, *"How have your symptoms been since the current COVID measures started?".* The responses were scored as decreased, stable, or increased. The association with possible explanatory variables was assessed through univariate ordinal regression. Data were analysed using R (R Core Team[17]) using the 'polr' function in the 'MASS' package.[18] A proportional chance criterion (PCC) test was used to determine if there was a significant difference in the proportion of participants who had increased compared to decreased symptoms assuming there was an equal likelihood of either.

A second analysis examined whether there were any prospective changes over the 10-week observation period in variables (pain, fatigue, anxiety, sleep, social networks, loneliness, resilience, optimism) that could be sensitive to change as the UK Government guidelines on social restriction measures altered (**Supplementary File 1**). There was a focus on differences in the two age groups (18-60 years and 60+ years) with the cut-off chosen to approximate to 'working' (n=353) and 'non-working' groups (n=354). Mixed models were used to analyse repeated data from the same participants. Time was included as a fixed-effect and 'participant ID' as a random-effect. All analyses were conducted in R using the 'clmm' function in the 'ordinal' package[19] for ordinal response variables or the 'lmer' function in the 'lme4' package[20] for continuous response variables.

Qualitative Study

The qualitative study was an embedded interview study which aimed to explore the subjective experiences of people with chronic musculoskeletal disease during COVID-19 social restrictions.

For peer review only - http://bmjopen.bmj.com/site/about/guidelines.xhtml

Sample and Recruitment

Survey participants recruited through NOAR were offered the option to take part in interviews; 137 expressed an interest. We designed a purposive sampling strategy, recruiting to achieve variation in age and gender, recognising that rheumatoid arthritis (RA) is more common in women and people aged over 40 years. A sample size of 25 to 30 was planned to support data collection in the window of guidance on self-isolation. This sample target was sufficient to enable robust themes to be developed. All those contacted agreed to take part.

Data Collection

The interview topic guide took a narrative approach, starting with open questions on everyday life and then focused on the participant's perspective on changes in symptoms, exercising, managing instrumental tasks such a shopping, medication and treatment regimens, and how they experienced changes in their social networks. Questions were reviewed by clinical and lay colleagues for clarity and relevance. Participants were approached by two researchers (LB, PB) over the telephone or email and received study information 72 hours prior to interview. All gave recorded verbal or written consent before the interview commenced. All interviews were conducted by two experienced qualitative researchers (LB, PB) over the telephone due to COVID-19 restrictions and were audio recorded for transcription. Interviews lasted between 30 and 90 minutes; average duration was 45 minutes.

Data Analysis

Data were analysed using a thematic analysis approach guided by the six-steps outlined by Braun and Clarke.[21] As a first step, interviews were transcribed and read to aid familiarization with the data. Two researchers (LB, PB) coded the data in NVivo 12. During coding, descriptive categories were identified relating to symptom attribution, changes to everyday life and wellbeing, narrative of vulnerability, and risk management. Frequent researcher and multidisciplinary meetings led to interpretive themes.[22] To enhance the dependability and confirmability of the results, peer and participants validation was completed.[23] Emerging themes and categories were shared across the research team and rheumatology health practitioners. Interview participants were sent a summary of key themes over email, followed by a phone call to each seeking feedback during August-September 2020. There was strong resonance between the themes and participants' experiences. The qualitative data were reviewed alongside the survey data to provide detailed explanations of experience of symptoms and wellbeing since restrictive measures commenced.

PATIENT AND PUBLIC INVOLVEMENT

Patient involvement began during protocol development stage of the study protocol and continued throughout. A patient-member (SW) provided her personal insights on the questions posed in the survey and qualitative topic guide. She provided advice into the interpretation of the survey and interview analysis. Our patient member assisted in the preparation of the final paper. She will continue to support dissemination through the preparation of public documents and social media outputs to share the results to wider patient communities.

RESULTS

Survey Results

For peer review only - http://bmjopen.bmj.com/site/about/guidelines.xhtml

Baseline Assessment

In total, 708 respondents (578 females, 127 males, three others or non-binary) were recruited (267 from NOAR and 441 from the wider-UK base). Their characteristics are summarised in **Table 1**. The majority of respondents were aged 51 to 70 (511/708; 72.2%) and residing in England (665/708; 93.9%). A range of inflammatory and non-inflammatory diseases were represented with RA (44.6%) and osteoarthritis (21.5%) reported most frequently. There were no important differences in the characteristics or responses in the NOAR and non-NOAR identified RA patients, and their data were pooled for analysis.

Table 2 illustrates respondent's symptoms at baseline compared with before the imposition of the COVID-19 social restriction measures. In total, 366 (52%) reported an increase in symptoms related to their musculoskeletal disease, 289 (41%) reported that they had stayed the same and 45 (6%) reported decreased reduction in symptoms (increased vs. decreased; p<0.001).

The increase in symptoms was greater in those who were younger (age ranged 18 to 60 years) (p=0.012). People who experienced an increase in symptoms were more likely to have difficulty accessing medication (p=0.04), were more likely to have changed their medication (p<0.001) and have needed access medical advice (p<0.001). There was a decrease in physical activity across the groups as a whole. This decrease was greatest in those reporting increased symptoms (**Table 2**).

Follow-up Assessment

Figure 1 and **Figure 2** illustrate the changes in responses over 10 weeks stratified by age (the strongest predictor of the increased level of symptoms). For all variables examined (symptoms, supportive social networks, loneliness, resilience and optimism), the younger age groups (18 to 60 years) fared worse than the older group (60 years plus) across all time points. Levels of optimism amongst the younger age groups fell into the range that is classified as 'low'. For the 18 to 60 years age group, there was a significant improvement in the levels of pain (p<0.001) and sleep (p<0.001), over the 10-week period, whilst anxiety levels (p=0.769) and fatigue (p=0.932) stayed the same. The 60 years plus age group had significant improvements in pain (p<0.001) and fatigue (p=0.001) but sleep (p=0.841) and anxiety (p=0.610) stayed the same over the 10 weeks (**Figure 1**). The size of any improvements was marginal in both age groups. In contrast, feelings of social isolation intensified (18 to 60 years: p<0.001; 60 years plus: p<0.001), and levels of resilience (18 to 60 years: p<0.001; 60 years plus: p<0.001) and optimism were significantly reduced (18 to 60 years: p=0.010; 60 years plus: p=0.013; **Figure 2**) but, the effect sizes were, again, small and unlikely to be of clinically relevant.

Qualitative Study

Interviews were undertaken with 26 participants between 27th May 2020 to 19th June 2020. The sample characteristics are presented in **Table 3**. The sample consisted of nine men and 17 women, with a mean age of 59 years (range: 29 to 83 years). Eighteen participants were diagnosed with RA, five with psoriatic arthritis, two with polymyalgia rheumatica and one with inflammatory polyarthritis. At the time of the baseline survey, 14 had reported increased symptoms, nine stable symptoms and three decreased symptoms.

Most participants described themselves as shielding, although there was variation in interpretation and adherence to the formal guidelines. There were narratives of resilience, acceptance and adaptive coping strategies:

> *I put up a little poster just to remind myself that I can only change what I am able to change. I just need to try and accept what I can't change.* [MSK1 Female 50-54]

For peer review only - http://bmjopen.bmj.com/site/about/guidelines.xhtml

Nonetheless, most highlighted challenges or stresses associated with the COVID-19 pandemic. In some, this was accompanied by reported worsening symptoms, including increased pain, stiffness and fatigue. Not all participants directly attributed their experience of symptoms to the COVID-19 context: *"My arthritis has flared up. I don't know if it's the change in situation or it was going to happen anyway."* However, many spoke about reasons related to the pandemic which they perceived had underpinned symptom changes. Several speculated that a combination of these factors may have been at play. The reasons are outlined thematically below and summarised in **Table 4.**

Accessing health care

Many of those interviewed described 'holding on' to concerns about their disease because they did not want to trouble health services at this time for fear of burdening the service, stating staff might be too busy due to COVID-19. Some had decided to miss blood tests due to anxiety around exposure to infection. Others were self-managing their symptoms:

> *If I get any breakthrough pain where I'm really, really not happy, then I can take two of my tramadol and…I take nortriptyline at night and I make sure that I'm getting a decent night's sleep. Therefore, I've just been doing a lot of self-care.* [MSK26 female 55-59]

Health concerns were also sometimes not reported due to postponed or cancelled appointments. There was a commonly expressed acceptance of this situation, feeling that others are 'worse off' at a time of crisis:

> *They cancelled my appointments…. under the circumstances, you accept it. It would have been nice to see someone because if your dosages are wrong, they can give you something or do something that would help. But obviously, I accept the situation and think well, there's people who are suffering and I'll just stand by.* [MSK6 Male 70-74]

However, missing or postponed appointments created anxiety and a sense of detachment from healthcare support for some, compounding delayed advice seeking: *"I would have brought it up [at postponed appointment] and it would have been good to discuss those things, and you do worry"* [MSK13 female 30-34]. Some had telephone appointments instead and although many preferred this, feeling it to be safer during the pandemic, some said that telephone appointments did not substitute entirely for face to face contact: *"They can't see the pain you're in; they can't feel your joints, they can't do any of that. And it's not their fault, obviously, but it does make everything so much trickier."* [MSK23 female 45-49]. Others explained that they avoided raising what they thought of as smaller concerns during a telephone call. One person described a physiotherapy telephone appointment which they acknowledged had been helpful, but also said *"[I] need human intervention for my body'"* [MSK10 female age 55-59].

Physical activity

Shielding instructions, lockdown and fear of catching COVID-19 meant many of those interviewed reported being much more sedentary and not leaving the house at all. The most common attribution for symptom change, including pain, stiffness and fatigue, was reduced activity.

> *I think that's just lack of movement, the lack of being able to move about. I think, over time, your body just shuts down due to lack of mobility.* [MSK10 female age 55-59]

Some discussed reduced motivation or limited opportunity to be able to engage in exercise such as walks or jogs outdoors while shielding, as well as being unable to access facilities such as gyms and swimming pools: " *I used to go to the gym a lot, just to force myself. But they're all closed."* [MSK 22 male 45-49] Reduced activity was also linked to disruption of everyday routine. This was more often

For peer review only - http://bmjopen.bmj.com/site/about/guidelines.xhtml

discussed by those of working age who described ceasing a regular daily walk or cycle commute to work, or being on their feet, not only in manual jobs but also within the context of office-based work:

> *Obviously, with me not working, then that's a huge change, isn't it, because I do a lot of walking during the day. Although, you look at it and just think 'well, that's admin', I actually spend my life going from one of the departments to another… I would say my exercise routine is pretty rubbish, I'll be honest I have put some weight on during lockdown. That could be part of [worse symptoms], let's face it."* [MSK1 female 50-54]

None reported having received specific advice about maintaining exercise for joint health at the time of interview, but a number were trying to substitute with exercise at home, (e.g. using a treadmill, online classes or walking round the garden). Exercising while avoiding contact with others was even more challenging without access to a private garden space:

> *I've got a long balcony here, it's about 100 yards long. So I should walk round there, but again, you've got to restrict that, because you never know if you're going to meet someone the other way. There isn't two metres to pass. So it restricts you from what you can do and what exercise you can get…. you find you're just getting stiffer and stiffer.* [MSK6 male 70-74]

Home work-space and tasks at home

Several interviewees explained they were working from home in environments which were far from ideal for the maintenance of good joint health, for example using a dining room chair and laptop rather than a desktop computer and office chair. Many envisaged home working for the foreseeable future. Levels of support and provision of equipment from employers was variable. Some employers had made swift efforts to prioritise provision of suitable equipment. However others had not received this support, and one participant was hesitant to complain for fear of losing their job at a time of job insecurity:

> *I'm just sitting at the kitchen table with my laptop. [My employer] said they couldn't do anything because there were so many of us that weren't at work. So to be honest, I was just like well, I'll just get on with it then, because I kind of need my job*. [MSK23 female 45-49]

In response to exacerbation of pain, some interviewees had purchased more ergonomic equipment themselves, while others felt they did not have the space to accommodate this kind of office set-up within their home.

A few participants who were shielding discussed no longer having access to assistance with household tasks which had adversely impacted on their physical or mental health, e.g. not having cleaners visit or because family members had moved out of the house during shielding. A few spoke of pacing out household activities but had not received professional advice on this.

Wellbeing

Descriptions of low mood, isolation and boredom were common in the interviews. Many explained they were anxious about being in a clinically-vulnerable group, and also reported a sense of being left behind or forgotten about. Many expressed worries about the future and some felt they would continue staying at home, regardless of relaxation of guidelines. A few said only a vaccine would make them feel 'safe'. Some made an explicit direct link with pain and fatigue symptoms, speculating for example that increased pain may be linked to stress surrounding their current situation. Others commented on the effect on their fatigue levels:

For peer review only - http://bmjopen.bmj.com/site/about/guidelines.xhtml

I've felt more tired, but I think that is because I felt more anxious and I haven't slept so well…..I'd say it's more linked to worrying about COVID and what it means managing that really. [MSK11 female 50-54]

For some with caring responsibilities, increased demands and stress associated with managing multiple roles at home, working while schools were closed, was identified as having contributed to symptom flare-up. There was also additional stress around decision-making and risk assessment in the context of family life, managing dilemmas about protecting their own health against wider family wellbeing.

It's been exhausting, absolutely exhausting. I was getting [child] to try and do his work, but he really needs one-to-one because he gets bored so quickly. So that became a bit of a nightmare… and then working in the evening to kind of catch up….no way of, if my arthritis is really bad, of just resting. [MSK23 female 45-49]

Improving or stable symptoms

In those who perceived no change in their condition, some attributed this to it having been in a stable, long-term state, for instance with disease was already well-controlled by medication or conversely was '*already bad*'. One participant who reported stable symptoms credited, in part, the support he had received from his employer prioritising him to receive appropriate office equipment for working at home. Others attributed their stable condition to maintaining routine, exercise and other protective coping behaviours. This retired person already had a daily routine in place for symptom management:

[My symptoms] seem to be about the same really. I know if I sit about I'll get stiff, so I do an exercise programme every day and try to keep myself moving around. [MSK16 female 75-79]

A small number of those interviewed explained that their symptoms had markedly improved as a consequence of COVID-19 measures. One cited better opportunity and motivation to take more exercise such as time to take walks, or joining children for daily exercises. Another participant described benefitting from being at home and a slower pace of activity, and that shielding had offered a period of respite during which they were better able to cope with their condition and prioritise self-care:

I'm furloughed….Having the time to just be at home, no one's expectations, my body has a chance to heal and find some balance… it makes you wonder. There's obviously no cure for RA at the moment but it does make you, you know, that emotional health is so key. [MSK 21 female 45-49]

Supplementary File 2 provides a summary of an interpretive synthesis of results, with interview findings, offering an explanatory insight into potential reasons for changes identified through the quantitative investigation.

DISCUSSION

We found that people with chronic musculoskeletal diseases frequently reported a deterioration in pain and symptoms, in addition to greater social isolation, loneliness and reduced optimism to their circumstances. This was significantly worse for those aged 18 to 60 years in comparison to older participants. The qualitative findings suggest that more types of change to daily life was experienced by those in employment. Although some retired people reported reduced opportunity for exercise outside the home, they did not face the many competing demands experienced by employed people and people with children at home.

For peer review only - http://bmjopen.bmj.com/site/about/guidelines.xhtml

Relationship to Previous Evidence

The findings that worsening symptoms and experiences occurred among younger, working-aged people may be attributed to the disruption in their occupations, changes in their home environment and loss of social networks. Our qualitative interviews indicated that the enforced COVID-19 social restrictions led to stressful changes in working routine, the addition of extra roles within the home (e.g. through home schooling) and deleterious workstation ergonomics due to lack of space and equipment. The latter point may be particularly important. Where occupational health requires work-station assessments for ergonomic safety, these were not practical in this phase of the pandemic. As people with pre-existing musculoskeletal diseases are particularly sensitive to the negative impacts of poor static postures, changes in workspace and working hours may have accounted for these reported physical responses.[24] As homeworking continues, employers should be aware of this challenge for employees with existing musculoskeletal diseases, to help reduce home issues that could lead to symptom flares.

Clinical Implications

The findings have implications for clinical practice. Firstly, the cohort we recruited largely demonstrated persistence in their worsening symptoms over time. As the qualitative research highlighted, reluctance to engage with health services, particularly during times of higher perceived risk, may mean that individuals are less likely to seek help for physical and mental health or only seek help when their symptoms become more severe, which may be more challenging to manage. Advertising and promoting services and how to access these may therefore be important strategies across primary and secondary care services to negate this issue. Secondly, due to changes in daily routine and increased symptoms, there may be greater disability within this population. Support to encourage resumption of physical activity routines and encouraging activity and engagement may be important for all.[25,26] Finally, these findings indicate that younger, working aged-people with musculoskeletal diseases were particularly affected by COVID-19 restrictions. Specific consideration of the impact on these individuals should be considered as they, potentially unexpectedly, may be at considerable risk of poorer musculoskeletal outcomes compared to older individuals. Employers should ensure that their workers, whilst working from home, have suitable risk assessments and provision of equipment to minimise the risks of exacerbating musculoskeletal pain, particularly for those with pre-existing musculoskeletal diseases.

In the months after the data for this paper were collected (August 2020), some enforced changes have been lifted through the resumption of schools and some workplaces. However, for many people in the UK and internationally, measures to restrict social activity remain and their impacts are ongoing which suggests the findings from this study remain pertinent.[27] Furthermore, whilst some activities can be recommenced, such as attending hospital appointments and communal exercise centres, many people reported longer-term concerns regarding their risk of COVID-19 and have refrained from resuming their normal routines. Additional support may be required to encourage individuals with the greatest anxiety to return to some of these social pursuits. This will be particularly important to avoid problems associated with social isolation, once longer-term restrictions are lifted. Previous literature has demonstrated the association between social isolation and higher incidence of cardiovascular disease,[28,29] mental conditions,[30] dementia[31] and mortality.[32]

Strengths and Limitations

This study is a large, national cohort of individuals with musculoskeletal diseases. The mixed-methods approach provides novel and important real-time exploratory and explanatory findings to the changes

For peer review only - http://bmjopen.bmj.com/site/about/guidelines.xhtml

in symptoms that this cohort reported during the initial 10 weeks of COVID-19 restrictions. Method triangulation[33] led to a more comprehensive understanding of the experiences of this specific group of people who may be considered particularly at risk of unintended consequences from social restrictions during the pandemic.

Whilst these are key strengths, the study presents with three important limitations. Firstly, whilst we can report the self-reported levels of disability and impairment, the sample were self-selecting when completing the survey. Whilst the characteristics of this cohort are typical of those with musculoskeletal diseases,[34] responder bias may have an impact on the external validity of these findings. Secondly, whilst we are able to make assumptions based on a cohort that includes a range of different musculoskeletal diseases, we acknowledged that only a small proportion of the population were BAME and we did not collect data to assess the respondents' level of social deprivation. Previous literature has highlighted these two factors to be potentially important in determining COVID-19 prognosis.[35] Including these groups may have provided different a different description of the behaviours and perceptions of these individuals towards COVID-19 and the impact on their symptoms. Finally, the qualitative study recruited people from the NOAR cohort who had also completed the survey. These were all patients with inflammatory rheumatological conditions. It remains unclear whether the responses those individuals included in our study can be applicable to non-inflammatory musculoskeletal conditions.

CONCLUSIONS

People with chronic musculoskeletal diseases frequently experienced deterioration in pain and symptoms when COVID-19 social restriction measures were enforced. As the restrictions continued over the following 10-weeks, levels of social isolation and loneliness increased and optimism decreased. These changes were greater among those aged 18 to 60 years when compared with older age groups. Close attention to those at risk through the promotion of physical activity, changing home-working practices and awareness of healthcare provision is important as social restrictions continue in the UK.

For peer review only - http://bmjopen.bmj.com/site/about/guidelines.xhtml

DECLARATIONS & ACKNOWLEDGEMENTS

Authors' Contributors: TS, PB, JD, KD, JC, JT, MY, FN, SW, CN, LS, AM researched the topic and devised the study; KD, JC, TS, MY performed the quantitative data collection; JD performed the statistical analyses; PB, LB performed the qualitative data collection; PB, LB performed the qualitative analysis; SW provided Patient and Public Involvement support; TS, PB, JD, KD, JC, JT, MY, FN, SW, CN, LS, AM provided the first draft of the manuscript; TS, PB, JD, KD, JC, JT, MY, FN, SW, CN, LS, AM contributed equally to manuscript preparation; TS, PB, JD, KD, JC, JT, MY, FN, SW, CN, LS, AM approved the submitted manuscript. AM and TS obtained research funding. AM acts a guarantor.

Funding: Funding was obtained to support the conduct of this study from Action Arthritis (Norfolk) and the University of East Anglia.

Ethical Considerations: Ethical approval was obtained from the University of East Anglia's Faculty of Medicine and Health Science's Research Ethics Committee (RE: 2019/20-104 & 2019/20-105)

Conflict of Interest: No author declares a conflict of interest in relation to this work.

Trial Sponsor: University of East Anglia (Research and Innovation Team, Registry and Council House, University of East Anglia, Norwich, NR4 7TJ. Email: RIN.reception@uea.ac.uk). The views expressed are those of the author(s) and not necessarily those of the sponsor.

Provenance and Peer Review: Not commissioned; externally peer reviewed.

Acknowledgements: The authors thank the following organisations who assisted in distributing the survey across their networks: Pain Concern; Arthritis Action; National Rheumatoid Arthritis Society; National Ankylosing Spondylitis Society; Paget's Association; Parathyroid UK; The Health Policy Partnership; and Scope.

Data Sharing: Data are available upon reasonable request. Additional data available upon request.

Patient consent for publication: Not required.

Word count: 4572

For peer review only

For peer review only - http://bmjopen.bmj.com/site/about/guidelines.xhtml

TABLE AND FIGURE LEGENDS

Figure 1: Change in symptoms from baseline over the 10-week follow-up interval.

Figure 2: Change in behaviour outcomes from baseline over the 10-week follow-up interval.

Table 1: Respondent characteristics and responses to health provision access from online survey at baseline.

Table 2: Association at baseline between musculoskeletal (MSK) symptoms and selected participant characteristics and questionnaire responses.

Table 3: Interview sub-sample characteristics (N=26).

Table 4: Key themes generated from the qualitative study - perceived underlying attributions for symptom change.

Supplementary File 1: Timeline of study activity against UK legislation and COVID policy implementation.

Supplementary File 2: Interpretive synthesis of quantitative and qualitative findings.

For peer review only

REFERENCES

1. GBD 2015 Disease and Injury Incidence and Prevalence Collaborators. Global, regional, and national incidence, prevalence, and years lived with disability for 310 diseases and injuries, 1990–2015: a systematic analysis for the Global Burden of Disease Study 2015. Lancet. 2016;388(10053):1545–1602.

2. Ethgen O, Vanparijs P, Delhalle S, Rosant S, Bruyère O, Reginster J-Y. Social support and health-related quality of life in hip and knee osteoarthritis. Quality of Life Research. 2004;13(2):321-30.

3. Veronese N, Smith T, Reginster JY, Maggi S. Osteoarthritis increases the risk of cardiovascular disease: data from the osteoarthritis initiative. J Nutr Health Aging. 2018;22(3):371-376.

4. Veronese N, Stubbs B, Solmi M, Smith TO, Noale M, Cooper C, et al. Association between lower limb osteoarthritis and incidence of depressive symptoms: data from the osteoarthritis initiative. Age Ageing. 2017;46(3):470-476.

5. McAlindon T, Cooper C, Kirwan J, Dieppe P. Determinants of disability in osteoarthritis of the knee. Annals of the rheumatic diseases. 1993;52(4):258.

6. Smith TO, Dainty JR, Williamson E, Martin KR. Association between musculoskeletal pain with social isolation and loneliness: analysis of the English Longitudinal Study of Ageing. Br J Pain. 2019;13(2):82-90.

7. Pope JE. What does the COVID-19 pandemic mean for rheumatology patients? Curr Treatm Opt Rheumatol. 2020;1-4.

8. Nune A, Iyengar K, Ahmed A, Sapkota H. Challenges in delivering rheumatology care during COVID-19 pandemic. Clin Rheumatol. 2020;39:2817-2821.

9. von Elm E, Altman DG, Egger M, Pocock SJ, Gotzsche PC, Vandenbroucke JP. The Strengthening the Reporting of Observational Studies in Epidemiology (STROBE) Statement: guidelines for reporting observational studies. J Clin Epidemiol. 2008;61(4):344-349.

10. Tong A, Sainsbury P, Craig J. Consolidated criteria for reporting qualitative research (COREQ): a 32-item checklist for interviews and focus groups. Int J Qual Health Care. 2007;19(6):349-357.

11. Symmons DP, Barrett EM, Bankhead CR, Scott DG, Silman AJ. The incidence of rheumatoid arthritis in the United Kingdom: results from the Norfolk Arthritis Register. Br J Rheumatol 1994;33:735–9.

12. Wolfe F. A brief clinical health assessment instrument: CLINHAQ. Arthritis Rheum. 1989;32 (suppl): S9.

13. Lubben J. Assessing social networks among elderly populations. Family Community Health. 1988;11:42-52.

14. Hughes ME, Waite LJ, Hawkley LC, Cacioppo JT. A short scale for measuring loneliness in large surveys. Results from two population-based studies. Res Aging. 2004;26:655-672.

For peer review only

15. Smith BW, Tooley EM, Christopher PJ, Kay VS. Resilience as the ability to bounce back from stress: a neglected personal resource? J Positive Psychol. 2010;5:166-176.

16. Scheier MF, Carver C, Bridges MW. Distinguishing optimism from neuroticism (and trait anxiety, self-mastery, and self-esteem): A re-evaluation of the Life Orientation Test. J Personality Social Psychol. 1994;67:1063-1078.

17. R Core Team. R: A language and environment for statistical computing. R Foundation for Statistical Computing, Vienna, Austria. Available at: www.R-project.org/. Accessed on: 04 Jan 2021.

18. Venables WN, Ripley BD. Modern applied statistics with S. Fourth Edition. Springer, New York, 2002

19. Christensen RHB. Ordinal - regression models for ordinal data. R package version 2019.12-10. Available at: CRAN.R-project.org/package=ordinal. Accessed: 04 Jan 2021.

20. Bates D, Maechler M, Bolker B, Walker S. Fitting linear mixed-effects models using lme4. J Statist Software. 2015;67(1):1-48.

21. Braun V, Clarke V. Using thematic analysis in psychology. Qualitative Res Psychol. 2006;3(2):77-101.

22. Morse JM. Confusing categorises and themes**.** Qualitative Health Res. 2008;18(6):727-728.

23. Birt L, Scott S, Cavers D, Campbell C, Walter F. Member checking: a tool to enhance trustworthiness or merely a nod to validation? Qualitat Health Res. 2016;26(13):1802-1811.

24. Baldwin D, Johnstone B, Ge B, Hewett J, Smith M, Sharp G. Randomized prospective study of a work place ergonomic intervention for individuals with rheumatoid arthritis and osteoarthritis. Arthritis Care Res. 2012;64:1527-1535.

25. Castro da Rocha FA, Melo LDP, Berenbaum F. Tackling osteoarthritis during COVID-19 pandemic. Ann Rheumat Dis. 2020. In Press. Online*:* doi: 10.1136/annrheumdis-2020-218372

26. Pinto AJ, Dunstan DW, Owen N, Bonfa E, Gualano B. Combating physical inactivity during the COVID-19 pandemic. Nat Rev Rheumatol. 2020;16:347–348.

27. Rogers NT, Waterlow NR, Brindle H, Enria L, Eggo RM, Lees S, et al. Behavioral change towards reduced intensity physical activity is disproportionately prevalent among adults with serious health issues or self-perception of high risk during the UK COVID-19 lockdown. Front Public Health. 2020;8:575091.

28. Valtorta NK, Kanaan M, Gilbody S, Ronzi S, Hanratty B. Loneliness and social isolation as risk factors for coronary heart disease and stroke: systematic review and meta-analysis of longitudinal observational studies. Heart. 2016;102(13):1009-1016.

29. Holt-Lunstad J, Smith TB. Loneliness and social isolation as risk factors for CVD: implications for evidence-based patient care and scientific inquiry. Heart. 2016;102(13):987-989.

30. Alpass FM, Neville S. Loneliness, health and depression in older males. Aging Mental Health. 2003;7(3):212-216.

For peer review only - http://bmjopen.bmj.com/site/about/guidelines.xhtml

31. Rafnsson SB, Orrell M, d'Orsi E, Hogervorst E, Steptoe A. Loneliness, social integration, and incident dementia over 6 years: prospective findings from the English Longitudinal Study of Ageing. J Gerontol B Psychol Sci Soc Sci2020; 75(1):114-124.

32. Steptoe A, Shankar A, Demakakos P, Wardle J. Social isolation, loneliness, and all-cause mortality in older men and women. Proc Natl Acad Sci USA. 2013;110(15):5797-5801.

33. Denzin NK. The research act: A theoretical introduction to sociological methods. New Jersey: Transaction Publishers, 1970.

34. Danielsson L, Kvarstein G, Bergvik S. Mediators of pain and physical function in female and male patients with chronic pain. J Pain Res. 2020;13:1059–1071.

35. Raisi-Estabragh Z, McCracken C, Bethell MS, Cooper J, Cooper C, Caulfield MJ, Munroe PB, Harvey NC, Petersen SE Greater risk of severe COVID- 19 in Black, Asian and Minority Ethnic populations is not explained by cardiometabolic, socioeconomic or behavioural factors, or by 25(OH)-vitamin D status: study of 1326 cases from the UK Biobank. J Public Health. 2020;42(3):451-460.

For peer review only

Table 1: Respondent characteristics and responses to health provision access from online survey at baseline

**Characteristics**		**Frequency (%)**
N		708
Gender	Female	578 (81.6)
	Male	127 (17.9)
	Non-binary	1 (0.1)
	Prefer to self-describe	1 (0.1)
	Prefer not to say	1 (0.1)
Age	18-30	21 (3.1)
	31-40	44 (6.2)
	41-50	114 (15.6)
	51-60	174 (24.8)
	61-70	204 (28.8)
	71-80	133 (18.8)
	80+	17 (2.4)
Region of respondent	England	665 (93.9)
	Scotland	21 (3.0)
	Wales	19 (2.7)
	Northern Ireland	2 (0.3)
Ethnic Group	White	687 (97.0)
	Mixed/Multiple ethnic group	4 (0.6)
	Asian	7 (1.0)
	Black, African, Caribbean	3 (0.4)
	Arab	1 (0.1)
Musculoskeletal (MSK) Diagnosis	Rheumatoid Arthritis	316 (44.6)
	Osteoarthritis	152 (21.5)
	Mechanical low back pain	70 (9.9)
	Fibromyalgia	68 (9.6)
	Psoriatic Arthritis	64 (9)
	Inflammatory Polyarthritis	63 (8.9)
	Hypermobility	40 (5.6)
	Specific MSK diagnosis not reported	33 (4.7)
	Connective tissue disease (e.g. Lupus, Scleroderma, Myositis)	25 (3.5)
	Ankylosing Spondylitis	22 (3.1)
	Osteoporosis	18 (2.5)
	Polymyalgia Rheumatica	10 (1.4)
	Ligament/Tendon Injury/Bursitis	9 (1.3)
	Neck pain	6 (0.8)
	Gout	5 (0.7)
	Other	63 (8.9)
How have your MSK symptoms been since the COVID measures started?	Decreased	45 (6.4)
	Stayed the same	289 (41.3)
	Increased	366 (52.3)
Pain (Scale: 0-10)	Mean (SD)	4.8 (2.6)
General Health (Scale: 0-10)	Mean (SD)	4.1 (2.4)
Total Lubben Social Network Score (Scale: 0-30)	Mean (SD)	14.8 (5.5)
Total UCLA Loneliness (Scale: 3-9)	Mean (SD)	5.1 (2.0)
Difficulty accessing medication	Yes	82 (11.7)
	No	619 (88.3)
Required someone to help access	Yes	312 (44.5)

For peer review only - http://bmjopen.bmj.com/site/about/guidelines.xhtml

medications	No	389 (55.5)
Changed medications since COVID-19 outbreak	Yes	103 (14.7)
	No	598 (85.3)
Required to seek advice from a health professional on condition	Yes	253 (36.1)
	No	448 (63.9)
Who did you contact	General Practitioner	158 (22.3)
	Practice Nurse/GP Nurse Practitioner	23 (3.2)
	Rheumatology Department	97 (13.7)
	Physiotherapy or Occupational Therapist	16 (2.3)
	Pharmacist	16 (2.3)
	Hospital Department (non-MSK)	10 (1.4)
	A&E	3 (0.4)
	Private chiropractor, osteopath or massage therapist	1 (0.1)
	Royal Osteoporosis Society	1 (0.1)
	Endocrinology Department	7 (1.0)
	Pain Clinic	2 (0.3)
	Counsellor or Health Psychologist	2 (0.3)
	Massage Therapist	1 (0.1)
	Podiatrist	1 (0.1)
	Nutritionist	1 (0.1)
	NHS 111	2 (0.3)
How easy has it been to get advice? (Scale: 0-10)	(Mean (SD) value. scale 0-10)	4.8 (3.3)

A&E- accident and emergency; CI – confidence intervals; JIA – Juvenile Inflammatory Arthritis; MSK – musculoskeletal; NHS – National Health Service; SD – standard deviation; UCLA – University of California Los Angeles Loneliness Score

For peer review only

For peer review only - http://bmjopen.bmj.com/site/about/guidelines.xhtml

Table 2: Association at baseline between musculoskeletal (MSK) symptoms and selected participant characteristics and questionnaire responses.

	"How have your symptoms been since the current COVID measures started?"			**Analysis of association with outcome (symptoms)**
	**Decreased**	**Stable**	**Increased**	p-value
n (%)	45 (6)	289 (41)	366 (52)	<0.001
Age group	60 Plus	60 Plus	18-60	0.012
Gender				
Male	8 (18)	60 (21)	59 (16)	reference
Female	37 (82)	226 (79)	315 (84)	0.068
MSK diagnosis*				
RA	17 (39)	156 (55)	152 (41)	reference
IA	4 (9)	25 (9)	13 (4)	0.037
PsA	4 (9)	22 (8)	39 (11)	0.101
Other	19 (43)	83 (29)	167 (45)	0.001
Situation				
At home	24 (55)	141 (49)	194 (52)	reference
Self-isolating	8 (18)	49 (17)	65 (18)	0.884
Shielding	13 (30)	99 (35)	111 (30)	0.419
Difficulty accessing Medication?				
Yes	4 (9)	26 (9)	52 (14)	reference
No	41 (91)	263 (91)	315 (86)	0.036
Change medication?				
Yes	9 (20)	21 (7)	73 (20)	reference
No	36 (80)	268 (93)	294 (80)	<0.001
Consult health professional?				
Yes	15 (33)	64 (22)	174 (47)	reference
No	30 (67)	225 (78)	193 (53)	<0.001
Physical activity				
Same	3 (7)	89 (31)	26 (7)	reference
Decreased	31 (69)	142 (49)	304 (83)	<0.001
Increased	11 (24)	58 (20)	37 (10)	0.390

Data are frequency (%) unless stated otherwise

* RA=Rheumatoid arthritis, IA=Inflammatory arthritis, PsA=Psoriatic arthritis, Other=mainly Osteoarthritis (55%) but also including any diagnosis that wasn't RA, IA or PsA (see **Table 1**)

For peer review only

For peer review only - http://bmjopen.bmj.com/site/about/guidelines.xhtml

Table 3: Interview sub-sample characteristics (N=26)

Characteristics		Frequency
**Gender**	Female	17
	Male	9
**Age**	18-30	1
	31-40	4
	41-50	5
	51-60	4
	61-70	3
	71-80	8
	80+	1
**Ethnic Group**	White	26
**Musculoskeletal (MSK) Diagnosis**	Rheumatoid Arthritis	18
	Psoriatic Arthritis	5
	Inflammatory Polyarthritis	1
	Polymyalgia Rheumatica	2
**How have your MSK symptoms been since the COVID measures started? (reported at baseline)**	Decreased	3
	Stayed the same	9
	Increased	14

For peer review only

Table 4: Key themes generated from the qualitative study - perceived underlying attributions for symptom change

Experience of symptoms	Attributions from interview data	Representative quote
**Increased symptoms**	**Accessing healthcare**<br>Holding on to concerns	*I don't like to worry them, they've got enough to do … It's something that I feel I can manage until when this is all over hopefully I'll get an appointment to go in and I'll mention it then.* [MSK17 Female age 70-74]
	Postponement or cancelation of appointments, treatments and investigations	*I think that's something I feel like I've missed out on with the lockdown is, I would actually quite have liked to have some proper physio, especially on the knees and that. Because the swelling just doesn't seem to want to go, as much as I poke and prod it and things like that.* [MSK 10 Female age 50-59]<br><br>*It was planned for me to have an MRI scan on my neck, but then this all happened and it's just sort gone out the window…. It's quite hard, day-to-day, with the pain levels in my neck, but under the current situation, I would rather put up with the pain than put myself at risk by going into a hospital.* [MSK20 Male age 50-55]<br><br>*I would have had a rheumatology appointment at the hospital during lockdown, but that was cancelled….I have some concerns at the moment and, again, I haven't done anything about it. I've just sort of put it all away.* [MSK Female age 40-44]
	Telephone appointments	*Where I've missed out because of the lockdown, is that although when I phoned the rheumatology department and things like that, they've been really helpful, it's not like having proper management, if that makes any sense. So all of the questions that I would normally have asked, I don't seem to ask so much on the phone….. It would be nice to say to somebody, "This bit hurts, what can I do about it? Is this any good?" and things like that. But you need to have the physical and visual side of that.* [MSK 10 Female age 50-54]
	**Reduced physical activity**<br>Reduced planned exercise (including less opportunity or access to facilities to exercise)	*I think I'm so much less active, before all this I was going swimming three times a week, that was the best exercise for me really, because it didn't put as much pressure on my body as going to the gym, running or anything like that. And, I don't think I fully appreciated how much swimming helped, whereas now, obviously, I'm not doing anything and I am just only getting out of the house for a little bit once a week, I've just found everywhere has just stiffened up so much…It's a bit scary, really.* [MSK5 female 25-29]<br><br>*[My joints are] getting stiff because you don't go out much to get exercise…. sitting down a lot makes your hips an your back ache and don't get up often where your chair is.* [MSK6 Male 70-74]<br><br>*I've got swelling in my fingers, which I haven't had for a long time, since probably my 20s…..I don't know if it's right, but I really was an active person. So, I would do like 15,000 steps easily in a day. It depends if I did a run, it would be more. I think it's lack of mobility.* [MSK2 Female 35-39]
	Reduced routine activity (e.g. walking to shops, going to work)	*I've been finding, like, sitting at my desk all day for work, I do get quite sore and quite stiff after work, because I think maybe when I was in an office environment I would be going out at lunchtime or just walking around the office to talk to different people.,,,, generally if I'm not sat at my desk working, I suppose, I'm sat on the sofa, I'm not really that mobile around the house.* [MSK5 female 25-29]<br><br>*[Pain and stiffness] have been a bit more apparent because I haven't been able to get out as much and use my joints, if that makes sense. So sometimes I find when I've been cooped up in a space and sitting down for a while, then obviously it affects you more. Obviously when I go to work…I can walk to work and back again and it frees things up a little bit. But obviously not being able to do that, it's not the same… it gets worse when you can't be as mobile as you could be.* [MSK12 male 35-39]

For peer review only - http://bmjopen.bmj.com/site/about/guidelines.xhtml

	**Working at home** Lack of ergonomic work-space	*At work I have a Varidesk…so, I can stand up. I also have like a proper chair, like a medical chair… [Working at home] was horrendous because I was working off my lap on a laptop, bending over. So, my back and my shoulder was very, very painful...* [MSK2 Female 35-39]
	Struggling with household tasks	*I used to have someone come on a Thursday and do my chores for me…So perhaps it's something like that as well, you know, sort of without the help there…. I think with the loss of energy you get more of a hurting in the legs at night and that.* [MSK19 female 70-74]
	**Wellbeing** Managing multiple roles within the home (Home-schooling, childcare and work)	*I probably went way too hard on myself, trying to plan lessons for [children]. You're also then working, you're also trying to run the house… completely overdid it……I do think the flare that I had was related to being in lockdown. I'm not sure I would have had it that bad if we were out of lockdown, because I would have been able to maybe adjust working life, the girls would have been at school, or my parents would have had them…. It was definitely probably reactive from overdoing it… trying to juggle so many plates* [MSK13 female 30-34]
	Anxiety or low mood associated with the pandemic and being in a clinically vulnerable group.	*I do struggle quite a lot with my mental health and I've found that my physical health has got so much worse, as well. So, there have been some days, especially when I was furloughed, where I just thought, I don't have to get out of bed today so let's just let it happen…. it's definitely harder at the moment.* [MSK5 female 25-29]<br><br>*Currently, I've got swelling ….and I've got pain in multiple areas. So, I would say I'm having a flare up…maybe it's the stress had an impact on my immune system* [MSK2 female 35-39]
**Stable symptoms**	Maintaining exercise	*In terms of my arthritis and stuff, that's not been – touch wood – too bad. I've tried to keep on top of it by trying to stay as active as I can really… … I've been probably doing 2K, 3K, 5Ks a week and then us, as a family unit, we try and go for a walk…so the pain has not been too bad.* [MSK14-male-35-39]
	Pre-existing stable or well-controlled condition	*I don't think there's a lot of difference really, it's quite well controlled anyway.* [MSK4 female 65-69]<br><br>*I haven't had a lot of trouble with joint pain….the methotrexate seems to be, you know, doing the trick.* [MSK15 male 75-79]
**Decreased symptoms**	An opportunity for respite, improved wellbeing	*The arthritis was getting worse [and since lockdown] I've had no flare ups. It's all been very much under control, and I think that's partly being removed from the work situation but it's partly having that easier pace of life, and if my body needs to sleep there's not an alarm set, it just sleeps. I think before this I was feeling very tired and unwell….I now feel far calmer, physically rested, which helps no end.* [MSK21 female 45-49]
	More opportunity for exercise	*I'm actually feeling actually really good right now….I like to think it's the exercises …..I've definitely done more daily exercises with my son and skipping and stuff and more bike rides. I like cycling anyway, but I've been on more long bike rides and probably been taking the dog for a walk probably more than I'd done before.* [MSK11 female 50-54]

2

3

For peer review only - http://bmjopen.bmj.com/site/about/guidelines.xhtml

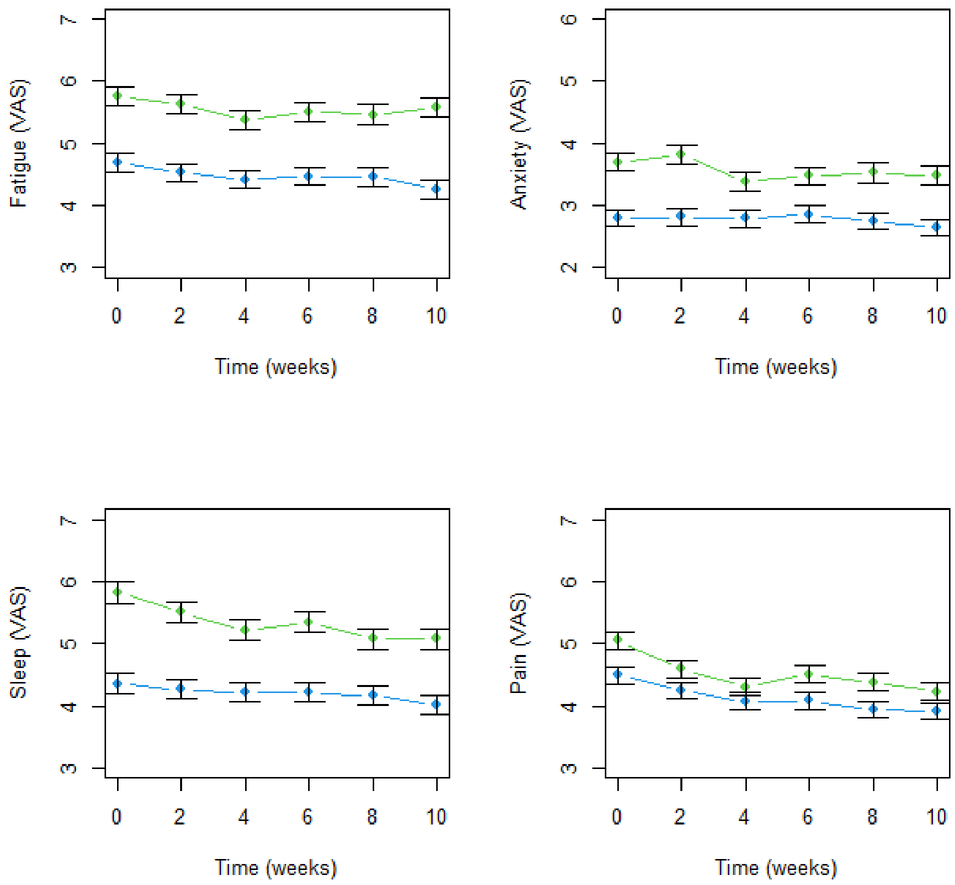

Figure 1: Change in symptoms from baseline over the 10-week follow-up interval.

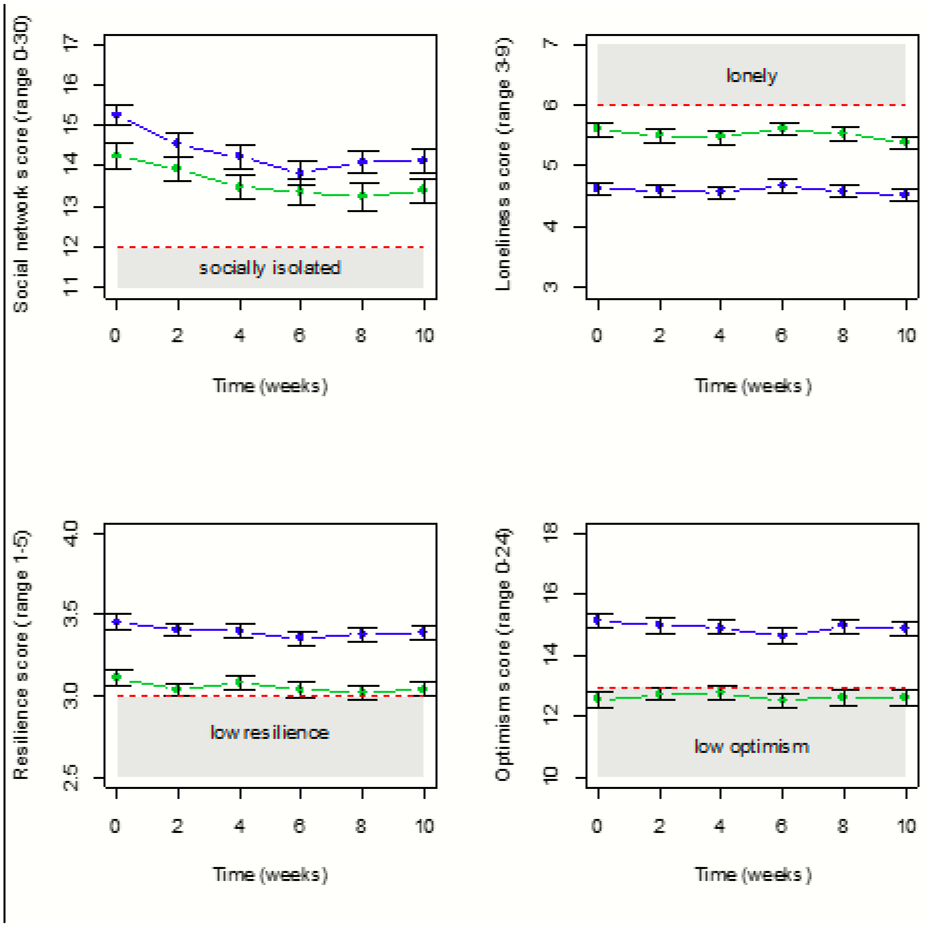

Figure 2: Change in behaviour outcomes from baseline over the 10-week follow-up interval.

Supplementary File 1: Timeline of study activity against UK legislation and COVID policy implementation.

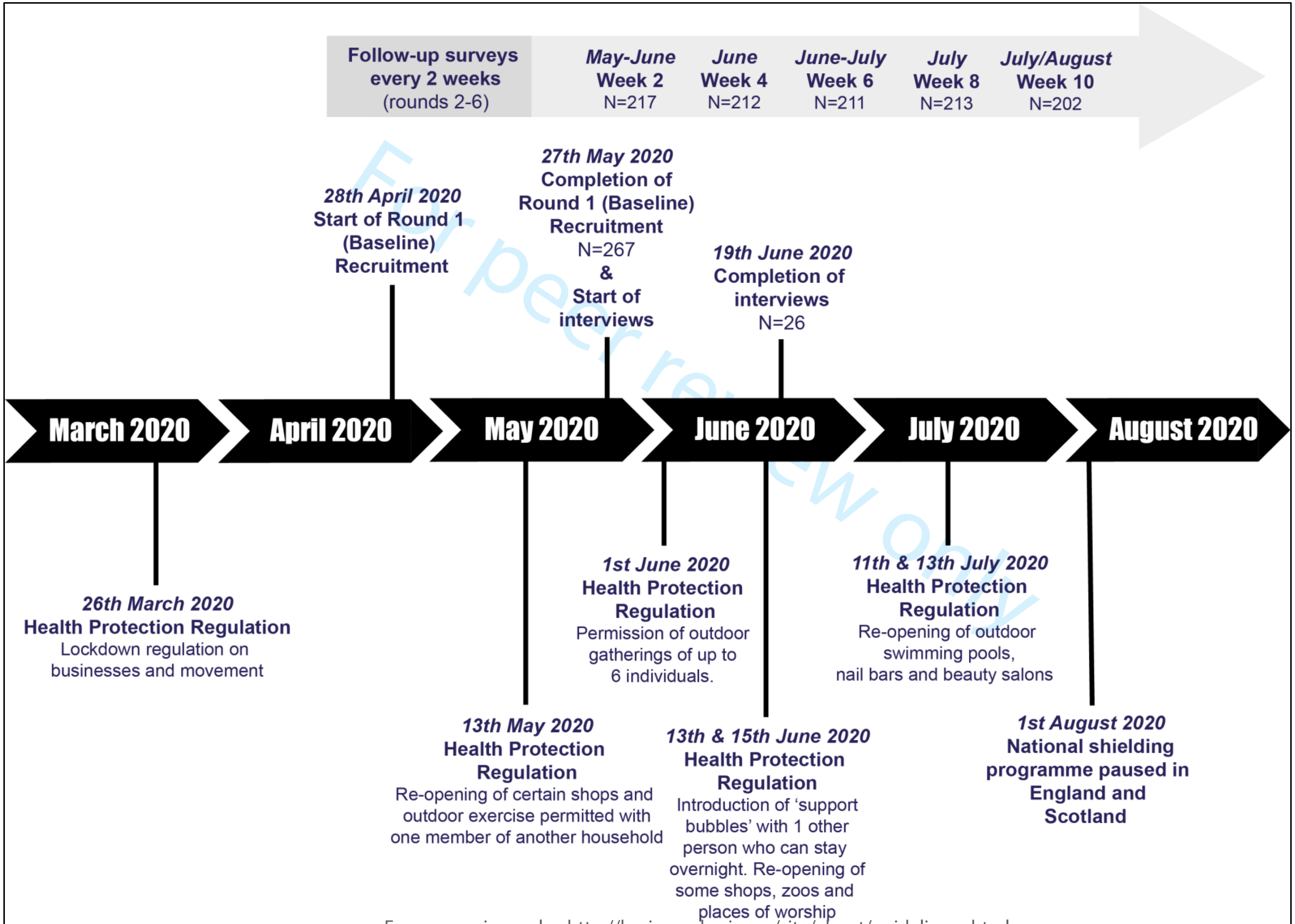

For peer review only - http://bmjopen.bmj.com/site/about/guidelines.xhtml

Supplementary File 2: Interpretive synthesis of quantitative and qualitative findings.

Survey Findings (See Tables 1-2)	Interview Findings (See Tables 3-4)
People with musculoskeletal disease reported an increase in pain and symptoms	*Attributions from symptom increase:*<br>- Changes in access to healthcare<br>- Reduced physical activity<br>- Ergonimics of working at home<br>- Reduced wellbeing<br>- Increased demands at home
Those who reported decreased physical activity were more likely to report increased symptoms	*Increase in symptoms attributed to:*<br>- Reduced routine activitiy<br>- Reduced opportunities for exercise
Increase in symptoms was significantly worse for those aged 18-60 years of age in comparison to older respondents	*Changes to daily life described by working age people included:*<br>- Disrupted daily routines<br>- Managing multiple roles at home, including childcare<br>- Unsuitable home working environment
Over the 10 weeks, greater social isolation, loneliness and reduced optimism were reported	*Feelings of low mood, boredom, social isolation or anxiety were related to:*<br>- Experience of restrictive measures<br>- Being in a clinically vulnerable group<br>- Disease symptoms<br>- A sense of being forgotten about
Half of those (53%) with increased symptoms at baseline had not consulted a healthcare professional	*Disrupted access to healthcare:*<br>- 'Holding on to concerns', wary of burdening healthcare staff, or uncertainty around service availability<br>- Reservations about telephone consultations<br>- Appointments, treatments and investigations postponed or cancelled

STROBE Statement—checklist of items that should be included in reports of observational studies

	Item No	Recommendation	Page No
**Title and abstract**	1	(*a*) Indicate the study's design with a commonly used term in the title or the abstract	Title Page
		(*b*) Provide in the abstract an informative and balanced summary of what was done and what was found	Abstract
**Introduction**			
Background/rationale	2	Explain the scientific background and rationale for the investigation being reported	Intro Para 1-3
Objectives	3	State specific objectives, including any prespecified hypotheses	Intro Para 4
**Methods**			
Study design	4	Present key elements of study design early in the paper	Methods Para 1
Setting	5	Describe the setting, locations, and relevant dates, including periods of recruitment, exposure, follow-up, and data collection	Methods Para 2-4, 9
Participants	6	(*a*) *Cohort study*—Give the eligibility criteria, and the sources and methods of selection of participants. Describe methods of follow-up *Case-control study*—Give the eligibility criteria, and the sources and methods of case ascertainment and control selection. Give the rationale for the choice of cases and controls *Cross-sectional study*—Give the eligibility criteria, and the sources and methods of selection of participants	Methods, Para 2, 8
		(*b*) *Cohort study*—For matched studies, give matching criteria and number of exposed and unexposed *Case-control study*—For matched studies, give matching criteria and the number of controls per case	Not applicable
Variables	7	Clearly define all outcomes, exposures, predictors, potential confounders, and effect modifiers. Give diagnostic criteria, if applicable	Methods Para 4,6
Data sources/ measurement	8*	For each variable of interest, give sources of data and details of methods of assessment (measurement). Describe comparability of assessment methods if there is more than one group	Methods Para 4
Bias	9	Describe any efforts to address potential sources of bias	Discussion Para 5,6
Study size	10	Explain how the study size was arrived at	Methods, Para 3, 6, 10
Quantitative variables	11	Explain how quantitative variables were handled in the analyses. If applicable, describe which groupings were chosen and why	Methods, Para 7, 8
Statistical methods	12	(*a*) Describe all statistical methods, including those used to control for confounding	Methods, Para 7, 8
		(*b*) Describe any methods used to examine subgroups and interactions	Methods, Para 8
		(*c*) Explain how missing data were addressed	Methods Para 8
		(*d*) *Cohort study*—If applicable, explain how loss to follow-up was addressed	Not applicable

For peer review only - http://bmjopen.bmj.com/site/about/guidelines.xhtml

		Case-control study—If applicable, explain how matching of cases and controls was addressed<br>*Cross-sectional study*—If applicable, describe analytical methods taking account of sampling strategy	
		(*e*) Describe any sensitivity analyses	Methods Para 8
**Results**			
Participants	13*	(a) Report numbers of individuals at each stage of study—eg numbers potentially eligible, examined for eligibility, confirmed eligible, included in the study, completing follow-up, and analysed	Results, Para 1
		(b) Give reasons for non-participation at each stage	Results, Para 1
		(c) Consider use of a flow diagram	Considered but not prioritised
Descriptive data	14*	(a) Give characteristics of study participants (eg demographic, clinical, social) and information on exposures and potential confounders	Results, Para 1, Table 1
		(b) Indicate number of participants with missing data for each variable of interest	Results, Para 1, Table 1-3
		(c) *Cohort study*—Summarise follow-up time (eg, average and total amount)	Methods, Para 6
Outcome data	15*	*Cohort study*—Report numbers of outcome events or summary measures over time	Results, Para 1, Table 1-3
		Case-control study—Report numbers in each exposure category, or summary measures of exposure	No CC study
		Cross-sectional study—Report numbers of outcome events or summary measures	Not cross-sectional
Main results	16	(*a*) Give unadjusted estimates and, if applicable, confounder-adjusted estimates and their precision (eg, 95% confidence interval). Make clear which confounders were adjusted for and why they were included	Table 2
		(*b*) Report category boundaries when continuous variables were categorized	Methods, Para 8
		(*c*) If relevant, consider translating estimates of relative risk into absolute risk for a meaningful time period	Not applicable
Other analyses	17	Report other analyses done—eg analyses of subgroups and interactions, and sensitivity analyses	Results, Table 3
**Discussion**			
Key results	18	Summarise key results with reference to study objectives	Discussion Para 1
Limitations	19	Discuss limitations of the study, taking into account sources of potential bias or imprecision. Discuss both direction and magnitude of any potential bias	Discussion Para 5,6
Interpretation	20	Give a cautious overall interpretation of results considering objectives, limitations, multiplicity of analyses, results from similar studies, and other relevant evidence	Conclusion Para 1

For peer review only

For peer review only - http://bmjopen.bmj.com/site/about/guidelines.xhtml

Generalisability	21	Discuss the generalisability (external validity) of the study results	Discussion Para 5,6
**Other information**			
Funding	22	Give the source of funding and the role of the funders for the present study and, if applicable, for the original study on which the present article is based	Declarations

*Give information separately for cases and controls in case-control studies and, if applicable, for exposed and unexposed groups in cohort and cross-sectional studies.

Note: An Explanation and Elaboration article discusses each checklist item and gives methodological background and published examples of transparent reporting. The STROBE checklist is best used in conjunction with this article (freely available on the Web sites of PLoS Medicine at http://www.plosmedicine.org/, Annals of Internal Medicine at http://www.annals.org/, and Epidemiology at http://www.epidem.com/). Information on the STROBE Initiative is available at www.strobe-statement.org.

For peer review only

COREQ (COnsolidated criteria for REporting Qualitative research) Checklist

A checklist of items that should be included in reports of qualitative research. You must report the page number in your manuscript where you consider each of the items listed in this checklist. If you have not included this information, either revise your manuscript accordingly before submitting or note N/A.

Topic	Item No.	Guide Questions/Description	Reported on Page No.
**Domain 1: Research team and reflexivity**			
Personal characteristics			
Interviewer/facilitator	1	Which author/s conducted the interview or focus group?	
Credentials	2	What were the researcher's credentials? E.g. PhD, MD	
Occupation	3	What was their occupation at the time of the study?	
Gender	4	Was the researcher male or female?	
Experience and training	5	What experience or training did the researcher have?	
Relationship with participants			
Relationship established	6	Was a relationship established prior to study commencement?	
Participant knowledge of the interviewer	7	What did the participants know about the researcher? e.g. personal goals, reasons for doing the research	
Interviewer characteristics	8	What characteristics were reported about the inter viewer/facilitator? e.g. Bias, assumptions, reasons and interests in the research topic	
**Domain 2: Study design**			
Theoretical framework			
Methodological orientation and Theory	9	What methodological orientation was stated to underpin the study? e.g. grounded theory, discourse analysis, ethnography, phenomenology, content analysis	
Participant selection			
Sampling	10	How were participants selected? e.g. purposive, convenience, consecutive, snowball	
Method of approach	11	How were participants approached? e.g. face-to-face, telephone, mail, email	
Sample size	12	How many participants were in the study?	
Non-participation	13	How many people refused to participate or dropped out? Reasons?	
Setting			
Setting of data collection	14	Where was the data collected? e.g. home, clinic, workplace	
Presence of non-participants	15	Was anyone else present besides the participants and researchers?	
Description of sample	16	What are the important characteristics of the sample? e.g. demographic data, date	
Data collection			
Interview guide	17	Were questions, prompts, guides provided by the authors? Was it pilot tested?	
Repeat interviews	18	Were repeat inter views carried out? If yes, how many?	
Audio/visual recording	19	Did the research use audio or visual recording to collect the data?	
Field notes	20	Were field notes made during and/or after the inter view or focus group?	
Duration	21	What was the duration of the inter views or focus group?	
Data saturation	22	Was data saturation discussed?	
Transcripts returned	23	Were transcripts returned to participants for comment and/or	

For peer review only

Topic	Item No.	Guide Questions/Description	Reported on Page No.
		correction?	
**Domain 3: analysis and findings**			
Data analysis			
Number of data coders	24	How many data coders coded the data?	
Description of the coding tree	25	Did authors provide a description of the coding tree?	
Derivation of themes	26	Were themes identified in advance or derived from the data?	
Software	27	What software, if applicable, was used to manage the data?	
Participant checking	28	Did participants provide feedback on the findings?	
Reporting			
Quotations presented	29	Were participant quotations presented to illustrate the themes/findings? Was each quotation identified? e.g. participant number	
Data and findings consistent	30	Was there consistency between the data presented and the findings?	
Clarity of major themes	31	Were major themes clearly presented in the findings?	
Clarity of minor themes	32	Is there a description of diverse cases or discussion of minor themes?	

Developed from: Tong A, Sainsbury P, Craig J. Consolidated criteria for reporting qualitative research (COREQ): a 32-item checklist for interviews and focus groups. *International Journal for Quality in Health Care*. 2007. Volume 19, Number 6: pp. 349 – 357

Once you have completed this checklist, please save a copy and upload it as part of your submission. DO NOT include this checklist as part of the main manuscript document. It must be uploaded as a separate file.

For peer review only

BMJ Open

The impact of COVID-19 pandemic social restriction measures on people with rheumatic and musculoskeletal diseases in the UK: a mixed-methods study.

Journal:	*BMJ Open*
Manuscript ID	bmjopen-2021-048772.R1
Article Type:	Original research
Date Submitted by the Author:	04-Apr-2021
Complete List of Authors:	Smith, Toby O.; University of Oxford, Nuffield Department of Orthopaedics, Rheumatology and Musculoskeletal Sciences; University of East Anglia,<br>Belderson, P; University of East Anglia<br>Dainty, Jack; University of East Anglia Faculty of Medicine and Health Sciences<br>Birt, Linda; University of East Anglia, School of Health Sciences<br>Durrant, K; University of East Anglia<br>Chipping, JR; University of East Anglia<br>Tsigarides, J; University of East Anglia, Rheumatology Department; Norfolk and Norwich University Hospital, Rheumatology Department<br>Yates, M; University of East Anglia, Rheumatology Department; Norfolk and Norwich University Hospital, Rheumatology Department<br>Werry, S<br>Naughton, Felix; University of East Anglia Faculty of Medicine and Health Sciences, School of Health Sciences; University of East Anglia<br>Notley, Caitlin; University of East Anglia, Norwich Medical School<br>Shepstone, Lee; University of East Anglia, Norwich Medical School<br>MacGregor, Alex; University of East Anglia, Norwich Medical School
**Primary Subject Heading**:	Rheumatology
Secondary Subject Heading:	Epidemiology
Keywords:	Rheumatology < INTERNAL MEDICINE, Pain management < ANAESTHETICS, COVID-19, Musculoskeletal disorders < ORTHOPAEDIC & TRAUMA SURGERY

SCHOLARONE™ Manuscripts

I, the Submitting Author has the right to grant and does grant on behalf of all authors of the Work (as defined in the below author licence), an exclusive licence and/or a non-exclusive licence for contributions from authors who are: i) UK Crown employees; ii) where BMJ has agreed a CC-BY licence shall apply, and/or iii) in accordance with the terms applicable for US Federal Government officers or employees acting as part of their official duties; on a worldwide, perpetual, irrevocable, royalty-free basis to BMJ Publishing Group Ltd ("BMJ") its licensees and where the relevant Journal is co-owned by BMJ to the co-owners of the Journal, to publish the Work in this journal and any other BMJ products and to exploit all rights, as set out in our licence.

The Submitting Author accepts and understands that any supply made under these terms is made by BMJ to the Submitting Author unless you are acting as an employee on behalf of your employer or a postgraduate student of an affiliated institution which is paying any applicable article publishing charge ("APC") for Open Access articles. Where the Submitting Author wishes to make the Work available on an Open Access basis (and intends to pay the relevant APC), the terms of reuse of such Open Access shall be governed by a Creative Commons licence – details of these licences and which Creative Commons licence will apply to this Work are set out in our licence referred to above.

Other than as permitted in any relevant BMJ Author's Self Archiving Policies, I confirm this Work has not been accepted for publication elsewhere, is not being considered for publication elsewhere and does not duplicate material already published. I confirm all authors consent to publication of this Work and authorise the granting of this licence.

For peer review only

BMJ OPEN

TITLE PAGE

TITLE: The impact of COVID-19 pandemic social restriction measures on people with rheumatic and musculoskeletal diseases in the UK: a mixed-methods study.

AUTHORS: Smith TO,[1,2] Belderson P,[1] Dainty JR,[1] Birt L,[1] Durrant K,[1] Chipping JR,[1] Tsigarides J,[1,3] Yates M,[1,3] Naughton F,[1] Werry S,[4] Notley C,[1] Shepstone L,[1] Macgregor AJ[1,3]

AFFILIATIONS

1. Faculty of Medicine and Health Sciences, University of East Anglia, Norwich, UK
2. Nuffield Department of Rheumatology, Orthopaedics and Musculoskeletal Sciences, University of Oxford, Botnar Research Centre, Windmill Road, Oxford, UK
3. Rheumatology Department, Norfolk and Norwich University Hospital, Norwich, UK
4. Patient and Public Representative, Norwich, UK

Corresponding Author: Professor Alex Macgregor, Norwich Medical School, University of East Anglia, Norwich, NR4 7TJ, UK. Email: a.macgregor@uea.ac.uk

Word Count: 4524 manuscript

For peer review only

For peer review only - http://bmjopen.bmj.com/site/about/guidelines.xhtml

ABSTRACT

OBJECTIVES: To determine the impact of COVID-19 pandemic social restriction measures on people with rheumatic and musculoskeletal diseases (RMDs) and to explore how people adapted to these measures over time.

DESIGN: Mixed-methods investigation comprising a national online longitudinal survey and embedded qualitative study.

SETTING: UK online survey and interviews with community-dwelling individuals in the East of England.

PARTICIPANTS: People in the UK with chronic RMDs, were invited to participate in an online survey. A subsection of respondents were invited to participate in the embedded qualitative study.

PRIMARY AND SECONDARY OUTCOME MEASURES: The online survey, completed fortnightly over 10 weeks from April 2020 to August 2020, investigated changes in symptoms, social isolation and loneliness, resilience and optimism. Qualitative interviews were undertaken assessing participant's perspectives on changes in symptoms, exercising, managing instrumental tasks such a shopping, medication and treatment regimens, and how they experienced changes in their social networks.

RESULTS: 703 people completed the online survey. These people frequently reported a deterioration in symptoms as a result of COVID-19 pandemic social restrictions (52% reported increase vs. 6% reported a decrease). This was significantly worse for those aged 18 to 60 years compared to older participants (p=0.017). The qualitative findings from 30 individuals with chronic RMDs suggest that the greatest change in daily life was experienced by those in employment. Although some retired people reported reduced opportunity for exercise outside their homes, they did not face the many competing demands experienced by employed people and people with children at home.

CONCLUSIONS: People with chronic RMDs reported a deterioration in symptoms when COVID-19 pandemic social restriction measures were enforced. This was worse for working-aged people. Consideration of this at-risk group, specifically for the promotion of physical activity, changing home-working practices and awareness of healthcare provision is important, as social restrictions continue in the UK.

Keywords: coronavirus; rheumatology; chronic musculoskeletal diseases; social isolation; occupational adaptation; qualitative; survey

For peer review only

STRENGTHS AND LIMITATIONS OF THIS STUDY

- This study reports the impact of COVID-19 social restrictions during the first COVID-19 'lockdown' on the lived-experiences of people with chronic RMDs.
- This UK longitudinal survey of 703 people included an embedded qualitative study providing explanations on the quantitative results.
- A breadth of important health domains were examined over the initial COVID-19 10-week social restriction period, including pain, social isolation and loneliness, resilience and optimism.
- We only recruited UK residents so while the principles may apply to countries experiencing social restrictions due to COVID-19, results may not be generalisable beyond the UK.

For peer review only - http://bmjopen.bmj.com/site/about/guidelines.xhtml

INTRODUCTION

Rheumatic and musculoskeletal disease (RMDs) are a major cause of disability and reduced quality of life worldwide.[1] People with chronic RMDs experience pain, joint stiffness, fatigue and muscle weakness with resultant physical disability. These symptoms are frequently managed with a combination of physical activity and medications, which for some people include immunosuppressive drugs.

In March 2020, the World Health Organization (WHO) declared the outbreak of a novel coronavirus disease SARs-CoV-2 (COVID-19) to be a pandemic. On 16th March 2020, the UK government instructed all people aged 70 years and over to self-isolate in their homes for up to four months. This was extended to all people on 23rd March for an initial three-week period. People who were taking immunosuppressant medications or with chronic medical comorbidities were instructed to maintain strict self-isolation principles (often referred to as shielding) for an extended period of at least 12 weeks (**Supplementary File 1**).

At the outset, there was concern that people with RMDs may be particularly vulnerable to the unintended consequences of these 'lockdown' measures. They are recognised to be at increased risk of social isolation, through poor social support,[2] the frequent presence of other medical conditions[3,4] and functional disability.[5] Social isolation itself can affect levels of physical disability, psychological distress and pain in RMDs.[6] The loss of exercise opportunities, a key component of self-management programmes, risked increasing levels of disability and reducing mental wellbeing. COVID-19 pandemic measures also significantly impacted on routine healthcare access and NHS services.[7,8] Prior to this study, the resilience of this group to these enforced changes was unknown, as were likely long-term impacts.

This study explores the impact of COVID-19 pandemic social restriction measures on people with chronic RMDs and determines how people adapted to these measures over time. Our aim was to provide insight into the challenges and potential impacts of those most at risk, and to make recommendations as to how best to support people with RMDs throughout further social restriction measures and beyond.

METHODS AND ANALYSIS

Design

Mixed-methods investigation comprising a national online longitudinal survey and embedded qualitative study. The study is reported in accordance with the STROBE statement for reporting observational studies[9] and the COREQ statement for reporting qualitative research.[10]

Survey Study

Cohort and recruitment

We invited participants who self-reported a diagnosis of chronic (three months or longer) RMDs (disease of the bone, joint or muscle), and who lived in the UK to participate in an online survey.

We used three approaches to recruit potential participants. Firstly, we approached patient-based organisations (Versus Arthritis, Arthritis Action, Royal Osteoporosis Society, National Rheumatoid

For peer review only - http://bmjopen.bmj.com/site/about/guidelines.xhtml

Arthritis Society, National Ankylosing Spondylitis Society, Fibromyalgia UK, Age UK, PainConcern and Scope). All agreed to disseminate an invitation to the survey via their online patient forum or as an email. Secondly, we contacted members from an inception cohort of 1,396 people with inflammatory arthritis – the Norfolk Arthritis Register (NOAR).[11] Finally, we used research team members' Twitter accounts to publicise the study.

Data collection

An online survey administered through the Qualtrics platform (Qualitrics XM, https://www.qualtrics.com/uk/) was provided to all participants. The survey is presented in **Supplementary File 1**. Participant consent was obtained through the platform before proceeding to the study survey. The survey collected information on:

- age, gender, ethnicity, duration of symptoms, type of RMD, medical co-morbidities.
- clinical disease activity.
- changes in medication use, access to healthcare support, physical activity, disease symptoms since COVID-19 lock-down.
- Clinical Health Assessment Questionnaire (CLINHAQ)[12] to assess disability and symptom status including: pain, fatigue, sleep, anxiety.
- the Lubben Social Network Scale 6[13] – short assessment of social isolation.
- University of California, Los Angeles (UCLA) three-item loneliness scale.[14]
- 6-item Brief Resilience Scale (BRS-6).[15]
- revised Life Orientation Test (LOT-R).[16]

We opened Round 1 (baseline) recruitment from 28th April 2020 to 27th May 2020. We sent tweets weekly during Round 1 recruitment period. For those who completed Round 1, we sent the same survey at two-week intervals to investigate how symptoms, social isolation, loneliness, resilience and optimism changed over time (Rounds 2 to 6). We sent a reminder email to participants who missed a subsequent round. The final survey was completed on 20th August 2020.

Data analysis

The analysis first addressed the perceived changes in symptoms since the start of lockdown in response to the question, *"How have your symptoms been since the current COVID measures started?".* The responses were scored as decreased, stable, or increased. The influence of possible explanatory variables on the categories of symptom response was assessed through chi-squared tests. Data were analysed using R (R Core Team[17]).

A second analysis examined whether there were any prospective changes over the 10-week observation period in variables (pain, fatigue, anxiety, sleep, social networks, loneliness, resilience, optimism) that could be sensitive to change as the UK Government guidelines on social restriction measures altered (**Supplementary File 2**). There was a focus on differences in the two age groups (18-60 years and 60+ years) with the cut-off chosen to approximate to 'working' (n=351) and 'non-working' groups (n=351). Mixed models were used to analyse repeated data from the same participants. Time was included as a fixed-effect and 'participant ID' as a random-effect. All analyses were conducted in R using the 'clmm' function in the 'ordinal' package[18] for ordinal response variables or the 'lmer' function in the 'lme4' package[19] for continuous response variables.

Qualitative Study

The qualitative study was an embedded interview study which aimed to explore the subjective experiences of people with chronic RMDs during COVID-19 pandemic social restrictions.

For peer review only - http://bmjopen.bmj.com/site/about/guidelines.xhtml

Sample and recruitment

Survey participants recruited through NOAR were offered the option to take part in interviews; 137 expressed an interest. We designed a purposive sampling strategy, recruiting to achieve variation in age and gender, recognising that rheumatoid arthritis (RA) is more common in women and people aged over 40 years. A sample size of 25 to 30 was planned to support data collection in the window of guidance on self-isolation. This sample target was sufficient to enable robust themes to be developed. All those contacted agreed to take part.

Data collection

The interview topic guide (**Supplementary File 3**) took a narrative approach, starting with open questions on everyday life and then focused on the participant's perspective on changes in symptoms, exercising, managing instrumental tasks such a shopping, medication and treatment regimens, and how they experienced changes in their social networks. Questions were reviewed by clinical and lay colleagues for clarity and relevance. Participants were approached by two researchers (LB, PB) over the telephone or email and received study information 72 hours prior to interview. All gave recorded verbal or written consent before the interview commenced. All interviews were conducted by two experienced qualitative researchers (LB, PB) over the telephone due to COVID-19 restrictions and were audio recorded for transcription. Interviews lasted between 30 and 90 minutes; average duration was 45 minutes.

Data analysis

Data were analysed using a thematic analysis approach guided by the six-steps outlined by Braun and Clarke.[20] As a first step, interviews were transcribed and read to aid familiarization with the data. Two researchers (LB, PB) coded the data in NVivo 12. During coding, descriptive categories were identified relating to symptom attribution, changes to everyday life and wellbeing, narrative of vulnerability, and risk management. Frequent researcher and multidisciplinary meetings led to interpretive themes.[21] To enhance the dependability and confirmability of the results, peer and participants validation was completed.[22] Emerging themes and categories were shared across the research team and rheumatology health practitioners. Interview participants were sent a summary of key themes over email, followed by a phone call to each seeking feedback during August-September 2020. There was strong resonance between the themes and participants' experiences.

The qualitative data were reviewed alongside the survey data to provide detailed explanations of experience of symptoms and wellbeing since restrictive measures commenced. Through this, an interpretative synthesis of results from each research methodology was presented using a mixed-methods approach.

PATIENT AND PUBLIC INVOLVEMENT

Patient involvement began during protocol development stage of the study protocol and continued throughout. A patient-member (SW) provided her personal insights on the questions posed in the survey and qualitative topic guide. She provided advice into the interpretation of the survey and interview analysis. Our patient member assisted in the preparation of the final paper. She will continue to support dissemination through the preparation of public documents and social media outputs to share the results to wider patient communities.

For peer review only - http://bmjopen.bmj.com/site/about/guidelines.xhtml

RESULTS

Survey Results

Baseline assessment

In total, 703 respondents (574 females, 126 males, three others or non-binary) were recruited (262 from NOAR and 441 from the wider-UK base). Their characteristics are summarised in **Table 1**. The majority of respondents were aged 51 to 70 (508/703; 72.3%) and residing in England (660/703; 93.9%). A range of inflammatory and non-inflammatory diseases were represented with RA (44.2%) and osteoarthritis (21.6%) reported most frequently. There were no important differences in the characteristics or responses in the NOAR and non-NOAR identified RA patients, and their data were pooled for analysis.

Table 2 illustrates respondent's symptoms at baseline compared with before the imposition of the COVID-19 pandemic social restriction measures. In total, 365 (52%; 95% CI: 49% to 56%) reported an increase in symptoms related to their RMD, 287 (41%; 95% CI: 37% to 45%) reported that they had stayed the same and 45 (6%; 95% CI: 5% to 9%) reported decreased reduction in symptoms.

Age had a significant influence on the change in symptoms, with a higher proportion of younger respondents (age ranged 18 to 60 years) reporting an increase in symptoms (p=0.017). As might be expected, people who experienced an increase in symptoms were more likely to have changed their medication (p<0.001) and have needed to access medical advice (p<0.001). Physical activity also varied according to the change in reported symptoms (p<0.001): those who reported that their symptoms had increased also reported a decreased level of physical activity (**Table 2**).

Follow-up assessment

Approximately 25-30% of the participants were lost to attrition over the course of the study. At the second timepoint (week=2), there were 525 participants, at week=4 there were 506, at week=6 there were 540, at week=8 there were 521 and at week=10 there were 491 participants. **Supplementary File 4** compares the characteristics of participants at baseline and week=10 and indicates that they were similar. **Figure 1** and **Figure 2** illustrate the changes in responses over 10 weeks stratified by age (the strongest predictor of the increased level of symptoms). For all variables examined (symptoms, supportive social networks, loneliness, resilience and optimism), the younger age groups (18 to 60 years) fared worse than the older group (60 years plus) across all time points. Levels of optimism amongst the younger age groups fell into the range that is classified as 'low'. For the 18 to 60 years age group, there was a significant improvement in the levels of pain (p<0.001) and sleep (p<0.001), over the 10-week period, whilst anxiety levels (p=0.769) and fatigue (p=0.920) stayed the same. The 60 years plus age group had significant improvements in pain (p<0.001) and fatigue (p=0.002) but sleep (p=0.080) and anxiety (p=0.610) stayed the same over the 10 weeks (**Figure 1**). The size of any improvements was marginal in both age groups. In contrast, feelings of social isolation intensified (18 to 60 years: p<0.001; 60 years plus: p<0.001), and levels of resilience (18 to 60 years: p<0.001; 60 years plus: p<0.001) and optimism were significantly reduced (18 to 60 years: p=0.008; 60 years plus: p=0.009; **Figure 2**) but, the effect sizes were, again, small and unlikely to be of clinically relevant.

Qualitative Study

For peer review only - http://bmjopen.bmj.com/site/about/guidelines.xhtml

Interviews were undertaken with 26 participants between 27th May 2020 to 19th June 2020. The sample consisted of nine men and 17 women, with a mean age of 59 years (range: 29 to 83 years). Eighteen participants were diagnosed with RA, five with psoriatic arthritis, two with polymyalgia rheumatica and one with inflammatory polyarthritis. At the time of the baseline survey, 14 had reported increased symptoms, nine stable symptoms and three decreased symptoms (**Table 3)**.

Most participants described themselves as shielding, although there was variation in interpretation and adherence to the formal guidelines. There were narratives of resilience, acceptance and adaptive coping strategies:

> *I put up a little poster just to remind myself that I can only change what I am able to change. I just need to try and accept what I can't change.* [MSK1 Female 50-54]

Nonetheless, most highlighted challenges or stresses associated with the COVID-19 pandemic. In some, this was accompanied by reported worsening symptoms, including increased pain, stiffness and fatigue. Not all participants directly attributed their experience of symptoms to the COVID-19 context: *"My arthritis has flared up. I don't know if it's the change in situation or it was going to happen anyway."* However, many spoke about reasons related to the pandemic which they perceived had underpinned symptom changes. Several speculated that a combination of these factors may have been at play. The reasons are outlined thematically below and summarised in **Table 4.**

<u>Accessing health care</u>

Many participants described 'holding on' to concerns about their disease because they did not want to trouble health services for fear of burdening the service, stating staff might be too busy due to COVID-19. Some had decided to miss blood tests due to anxiety around exposure to infection. Others were self-managing their symptoms:

> *If I get any breakthrough pain where I'm really not happy, I can take two of my tramadol and…I take nortriptyline at night and I make sure that I'm getting a decent night's sleep. I've just been doing a lot of self-care.* [MSK26 female 55-59]

Health concerns were also sometimes not reported due to postponed or cancelled appointments. There was a commonly expressed acceptance of this situation, feeling that others are 'worse off' at a time of crisis: *They cancelled my appointments…under the circumstances, you accept it…and think well, there's people who are suffering and I'll just stand by.* [MSK6 male 70-74]

However, missing or postponed appointments created anxiety and a sense of detachment from healthcare support for some, compounding delayed advice seeking: *"I would have brought it up [at postponed appointment] and it would have been good to discuss those things, and you do worry"* [MSK13 female 30-34]. Some had telephone appointments instead and although many preferred this, feeling it to be safer during the pandemic, some said that telephone appointments did not substitute entirely for face to face contact: *"They can't see the pain you're in; they can't feel your joints, they can't do any of that…does make everything so much trickier"* [MSK23 female 45-49]. Others explained that they avoided raising what they thought of as smaller concerns during a telephone call. One person described a physiotherapy telephone appointment which they acknowledged had been helpful, but also said *"[I] need human intervention for my body"* [MSK10 female age 55-59].

<u>Physical activity</u>

Shielding instructions, lockdown and fear of catching COVID-19 meant many interviewed participants reported being much more sedentary and not leaving the house at all. The most common attribution for symptom change, including pain, stiffness and fatigue, was reduced activity: *"I think that's just*

For peer review only - http://bmjopen.bmj.com/site/about/guidelines.xhtml

lack of movement, the lack of being able to move about. I think, over time, your body just shuts down due to lack of mobility" [MSK10 female age 55-59].

Some discussed reduced motivation or limited opportunity to be able to engage in exercise: *" I used to go to the gym a lot, just to force myself. But they're all closed"* [MSK22 male 45-49]. Reduced activity was also linked to disruption of everyday routine. This was more often discussed by those of working age who described ceasing a regular daily walk or cycle commute to work, or being on their feet, not only in manual jobs but also within the context of office-based work:

> *Obviously, with me not working, then that's a huge change, I actually spend my life going from one of the departments to another… I would say my exercise routine is pretty rubbish, I'll be honest I have put some weight on during lockdown. That could be part of [worse symptoms], let's face it."* [MSK1 female 50-54]

None reported having received specific advice about maintaining exercise for joint health at the time of interview, but a number were trying to substitute with exercise at home, (e.g. using a treadmill, online classes or walking round the garden). Exercising while avoiding contact with others was even more challenging without access to a private garden space:

> *I've got a long balcony here, it's about 100 yards long. So I should walk round there, but again, you've got to restrict that, because you never know if you're going to meet someone the other way. There isn't two metres to pass….you find you're just getting stiffer and stiffer.* [MSK6 male 70-74]

<u>Home work-space and tasks at home</u>

Several interviewees explained they were working from home in environments which were far from ideal for the maintenance of good joint health, for example using a dining room chair and laptop rather than a desktop computer and office chair. Many envisaged home working for the foreseeable future. Levels of support and provision of equipment from employers was variable. Some employers had made swift efforts to prioritise provision of suitable equipment. However others had not received this support, and one participant was hesitant to complain for fear of losing their job at a time of job insecurity:

> *I'm just sitting at the kitchen table with my laptop. [My employer] said they couldn't do anything because there were so many of us that weren't at work. I was just like well, I'll just get on with it then, because I kind of need my job.* [MSK23 female 45-49]

In response to exacerbation of pain, some interviewees had purchased more ergonomic equipment themselves, while others felt they did not have the space to accommodate this kind of office set-up within their home.

A few participants who were shielding discussed no longer having access to assistance with household tasks which had adversely impacted on their physical or mental health. A few spoke of pacing out household activities but had not received professional advice on this.

<u>Wellbeing</u>

Descriptions of low mood, isolation and boredom were common in the interviews. Many explained they were anxious about being in a clinically-vulnerable group, and also reported a sense of being left behind or forgotten about. Many expressed worries about the future and some felt they would continue staying at home, regardless of relaxation of guidelines. A few said only a vaccine would make them feel 'safe'. Some made an explicit direct link with pain and fatigue symptoms, speculating for example that increased pain may be linked to stress surrounding their current situation. Others commented on the effect on their fatigue levels: *"I've felt more tired, but I think that is because I felt*

For peer review only - http://bmjopen.bmj.com/site/about/guidelines.xhtml

more anxious and I haven't slept so well…..I'd say it's more linked to worrying about COVID" [MSK11 female 50-54].

For some with caring responsibilities, increased demands and stress associated with managing multiple roles at home, working while schools were closed, was identified as having contributed to symptom flare-up. There was also additional stress around decision-making and risk assessment in the context of family life, managing dilemmas about protecting their own health against wider family wellbeing.

> *It's been exhausting, absolutely exhausting. I was getting [child] to try and do his work, but he really needs one-to-one because he gets bored so quickly. So that became a bit of a nightmare… and then working in the evening to kind of catch up….no way of, if my arthritis is really bad, of just resting.* [MSK23 female 45-49]

<u>Improving or stable symptoms</u>

In those who perceived no change in their condition, some attributed this to it having been in a stable, long-term state, for instance with disease already well-controlled by medication or conversely was "*already bad*". One participant who reported stable symptoms credited, in part, the support he had received from his employer prioritising him to receive appropriate office equipment for working at home. Others attributed their stable condition to maintaining routine, exercise and other protective coping behaviours: *[My symptoms] seem to be about the same really. I know if I sit about I'll get stiff, so I do an exercise programme every day and try to keep myself moving around."* [MSK16 female 75-79]

A small number of those interviewed explained that their symptoms had markedly improved as a consequence of COVID-19 measures. One cited better opportunity and motivation to take more exercise such as walks, or joining children for daily exercises. Another participant described benefitting from being at home and a slower pace of activity, and that shielding had offered a period of respite during which they were better able to cope with their condition and prioritise self-care:

> *I'm furloughed….Having the time to just be at home, no one's expectations, my body has a chance to heal and find some balance… it makes you wonder. There's obviously no cure for RA at the moment but it does make you, you know, that emotional health is so key.* [MSK21 female 45-49]

Supplementary File 5 provides a summary of an interpretive synthesis of results, with interview findings, offering an explanatory insight into potential reasons for changes identified through the quantitative investigation.

DISCUSSION

Within the survey cohort, we found that people with chronic RMDs frequently reported a deterioration in pain and symptoms, in addition to greater social isolation, loneliness and reduced optimism to their circumstances. This was significantly worse for those aged 18 to 60 years in comparison to older participants. The qualitative findings suggest that more types of change to daily life was experienced by those in employment. Although some retired people reported reduced opportunity for exercise outside the home, they did not face the many competing demands experienced by employed people and people with children at home.

<u>Relationship to previous evidence</u>

For peer review only - http://bmjopen.bmj.com/site/about/guidelines.xhtml

Recently, Persiani et al[23] reported their findings of the impact of the first COVID-19 pandemic 'lockdown' in Italy from April to May 2020 with 292 people with RMDs. These findings reflect those of our UK cohort which illustrated a reduction in physical activity and increase in pain, particularly amongst people aged 50 to 70 years and in work.[23] These findings may be attributed to a disruption in their normal working patterns, changes in their home environment and loss of social networks. Our qualitative interviews indicated that the enforced COVID-19 pandemic social restrictions led to stressful changes in working routine, the addition of extra roles within the home (e.g. through home schooling) and deleterious workstation ergonomics due to lack of space and equipment. The latter point may be particularly important. Where occupational health requires work-station assessments for ergonomic safety, these were not practical in this phase of the pandemic. As people with pre-existing RMDs are particularly sensitive to the negative impacts of poor static postures, changes in workspace and working hours may have accounted for these reported physical responses.[24] As homeworking continues, employers should be aware of this challenge for employees with existing RMDs, to help reduce home issues that could lead to symptom flares.

Clinical implications

The findings have implications for clinical practice. Firstly, the cohort we recruited largely demonstrated persistence in their worsening symptoms over time. As the qualitative research highlighted, reluctance to engage with health services, particularly during times of higher perceived risk, may mean that individuals are less likely to seek help for physical and mental health or only seek help when their symptoms become more severe, which may be more challenging to manage. Advertising and promoting services and how to access these may therefore be important strategies across primary and secondary care services to negate this issue. Secondly, due to changes in daily routine and increased symptoms, there may be greater disability within this population. Support to encourage resumption of physical activity routines and encouraging activity and engagement may be important for all.[25,26] Finally, these findings indicate that younger, working aged-people with RMDs were particularly affected by COVID-19 pandemic restrictions. Specific consideration of the impact on these individuals should be considered as they, potentially unexpectedly, may be at considerable risk of poorer musculoskeletal outcomes compared to older individuals. Employers should ensure that their workers, whilst working from home, have suitable risk assessments and provision of equipment to minimise the risks of exacerbating musculoskeletal pain, particularly for those with pre-existing RMDs.

In the months after the data for this paper were collected (August 2020), some enforced changes have been lifted. However, for many people in the UK and internationally, measures to restrict social activity remain and their impacts are ongoing. The findings from this study therefore remain pertinent.[27] Furthermore, whilst some activities can be recommenced, such as attending hospital appointments and communal exercise, many people reported longer-term concerns regarding their risk of COVID-19 and have refrained from resuming their normal routines. Additional support may be required to encourage individuals with the greatest anxiety to return to some of these social pursuits. This will be particularly important to avoid problems associated with social isolation, once longer-term restrictions are lifted. Previous literature has demonstrated the association between social isolation and higher incidence of cardiovascular disease,[28,29] mental conditions,[30] dementia[31] and mortality.[32]

Strengths and limitations

This study is a large, national cohort of individuals with RMDs. The mixed-methods approach provides novel and important real-time exploratory and explanatory findings to the changes in symptoms that

For peer review only - http://bmjopen.bmj.com/site/about/guidelines.xhtml

this cohort reported during the initial 10 weeks of COVID-19 pandemic restrictions. Method triangulation[33] led to a more comprehensive understanding of the experiences of this specific group of people who may be considered particularly at risk of unintended consequences from social restrictions during the pandemic.

Whilst these are key strengths, the study presents with three important limitations. Firstly, whilst we can report the self-reported levels of disability and impairment, the sample were self-selecting when completing the survey. Whilst the characteristics of this cohort are typical of those with RMDs,[34] responder bias may have an impact on the external validity of these findings. Secondly, whilst we are able to make assumptions based on a cohort that includes a range of different RMDs, we acknowledged that only a small proportion of the population were BAME and we did not collect data to assess the respondents' level of social deprivation. Previous literature has highlighted these two factors to be potentially important in determining COVID-19 prognosis.[35] Including these groups may have provided a different description of the behaviours and perceptions of these individuals towards COVID-19 and the impact on their symptoms. Finally, the qualitative study recruited people from the NOAR cohort who had also completed the survey. These were all patients with inflammatory rheumatological conditions. It remains unclear whether the responses those individuals included in our study can be applicable to non-inflammatory musculoskeletal conditions.

CONCLUSIONS

People with chronic RMDs frequently experienced deterioration in pain and symptoms when COVID-19 pandemic social restriction measures were enforced. As the restrictions continued over the following 10-weeks, levels of social isolation and loneliness increased and optimism decreased. These changes were greater among those aged 18 to 60 years when compared with older age groups. Close attention to those at risk through the promotion of physical activity, changing home-working practices and awareness of healthcare provision is important as social restrictions continue in the UK.

For peer review only

For peer review only - http://bmjopen.bmj.com/site/about/guidelines.xhtml

DECLARATIONS & ACKNOWLEDGEMENTS

Authors' Contributors: TS, PB, JD, KD, JC, JT, MY, FN, SW, CN, LS, AM researched the topic and devised the study; KD, JC, TS, MY performed the quantitative data collection; JD performed the statistical analyses; PB, LB performed the qualitative data collection; PB, LB performed the qualitative analysis; SW provided Patient and Public Involvement support; TS, PB, JD, KD, JC, JT, MY, FN, SW, CN, LS, AM provided the first draft of the manuscript; TS, PB, JD, KD, JC, JT, MY, FN, SW, CN, LS, AM contributed equally to manuscript preparation; TS, PB, JD, KD, JC, JT, MY, FN, SW, CN, LS, AM approved the submitted manuscript. AM and TS obtained research funding. AM acts a guarantor.

Funding: Funding was obtained to support the conduct of this study from Action Arthritis (Norfolk) and the University of East Anglia. Number reference number: N/A.

Ethical Considerations: Ethical approval was obtained from the University of East Anglia's Faculty of Medicine and Health Science's Research Ethics Committee (RE: 2019/20-104 & 2019/20-105)

Conflict of Interest: No author declares a conflict of interest in relation to this work.

Trial Sponsor: University of East Anglia (Research and Innovation Team, Registry and Council House, University of East Anglia, Norwich, NR4 7TJ. Email: RIN.reception@uea.ac.uk). The views expressed are those of the author(s) and not necessarily those of the sponsor.

Provenance and Peer Review: Not commissioned; externally peer reviewed.

Acknowledgements: The authors thank the following organisations who assisted in distributing the survey across their networks: Pain Concern; Arthritis Action; National Rheumatoid Arthritis Society; National Ankylosing Spondylitis Society; Paget's Association; Parathyroid UK; The Health Policy Partnership; and Scope.

Data Sharing: Data are available upon reasonable request. Additional data available upon request.

Patient consent for publication: Not required.

Word count: 4524

For peer review only

TABLE AND FIGURE LEGENDS

Figure 1: Change in symptoms from baseline over the 10-week follow-up interval.

Figure 2: Change in behaviour outcomes from baseline over the 10-week follow-up interval.

Table 1: Respondent characteristics and responses to health provision access from online survey at baseline.

Table 2: Association at baseline between musculoskeletal (MSK) symptoms and selected participant characteristics and questionnaire responses.

Table 3: Interview sub-sample characteristics (N=26).

Table 4: Key themes generated from the qualitative study - perceived underlying attributions for symptom change.

Supplementary File 1: Online survey (baseline)

Supplementary File 2: Timeline of study activity against UK legislation and COVID policy implementation.

Supplementary File 3: Interview topic guide

Supplementary File 4: Characteristics of participants at baseline and Week 10

Supplementary File 5: Interpretive synthesis of quantitative and qualitative findings

For peer review only - http://bmjopen.bmj.com/site/about/guidelines.xhtml

REFERENCES

1. GBD 2015 Disease and Injury Incidence and Prevalence Collaborators. Global, regional, and national incidence, prevalence, and years lived with disability for 310 diseases and injuries, 1990–2015: a systematic analysis for the Global Burden of Disease Study 2015. Lancet. 2016;388(10053):1545–1602.

2. Ethgen O, Vanparijs P, Delhalle S, Rosant S, Bruyère O, Reginster J-Y. Social support and health-related quality of life in hip and knee osteoarthritis. Quality of Life Research. 2004;13(2):321-30.

3. Veronese N, Smith T, Reginster JY, Maggi S. Osteoarthritis increases the risk of cardiovascular disease: data from the osteoarthritis initiative. J Nutr Health Aging. 2018;22(3):371-376.

4. Veronese N, Stubbs B, Solmi M, Smith TO, Noale M, Cooper C, et al. Association between lower limb osteoarthritis and incidence of depressive symptoms: data from the osteoarthritis initiative. Age Ageing. 2017;46(3):470-476.

5. McAlindon T, Cooper C, Kirwan J, Dieppe P. Determinants of disability in osteoarthritis of the knee. Annals of the rheumatic diseases. 1993;52(4):258.

6. Smith TO, Dainty JR, Williamson E, Martin KR. Association between musculoskeletal pain with social isolation and loneliness: analysis of the English Longitudinal Study of Ageing. Br J Pain. 2019;13(2):82-90.

7. Pope JE. What does the COVID-19 pandemic mean for rheumatology patients? Curr Treatm Opt Rheumatol. 2020;1-4.

8. Nune A, Iyengar K, Ahmed A, Sapkota H. Challenges in delivering rheumatology care during COVID-19 pandemic. Clin Rheumatol. 2020;39:2817-2821.

9. von Elm E, Altman DG, Egger M, Pocock SJ, Gotzsche PC, Vandenbroucke JP. The Strengthening the Reporting of Observational Studies in Epidemiology (STROBE) Statement: guidelines for reporting observational studies. J Clin Epidemiol. 2008;61(4):344-349.

10. Tong A, Sainsbury P, Craig J. Consolidated criteria for reporting qualitative research (COREQ): a 32-item checklist for interviews and focus groups. Int J Qual Health Care. 2007;19(6):349-357.

11. Symmons DP, Barrett EM, Bankhead CR, Scott DG, Silman AJ. The incidence of rheumatoid arthritis in the United Kingdom: results from the Norfolk Arthritis Register. Br J Rheumatol 1994;33:735–9.

12. Wolfe F. A brief clinical health assessment instrument: CLINHAQ. Arthritis Rheum. 1989;32 (suppl): S9.

13. Lubben J. Assessing social networks among elderly populations. Family Community Health. 1988;11:42-52.

14. Hughes ME, Waite LJ, Hawkley LC, Cacioppo JT. A short scale for measuring loneliness in large surveys. Results from two population-based studies. Res Aging. 2004;26:655-672.

For peer review only - http://bmjopen.bmj.com/site/about/guidelines.xhtml

For peer review only

15. Smith BW, Tooley EM, Christopher PJ, Kay VS. Resilience as the ability to bounce back from stress: a neglected personal resource? J Positive Psychol. 2010;5:166-176.

16. Scheier MF, Carver C, Bridges MW. Distinguishing optimism from neuroticism (and trait anxiety, self-mastery, and self-esteem): A re-evaluation of the Life Orientation Test. J Personality Social Psychol. 1994;67:1063-1078.

17. R Core Team. R: A language and environment for statistical computing. R Foundation for Statistical Computing, Vienna, Austria. Available at: www.R-project.org/. Accessed on: 04 Jan 2021.

18. Christensen RHB. Ordinal - regression models for ordinal data. R package version 2019.12-10. Available at: CRAN.R-project.org/package=ordinal. Accessed: 04 Jan 2021.

19. Bates D, Maechler M, Bolker B, Walker S. Fitting linear mixed-effects models using lme4. J Statist Software. 2015;67(1):1-48.

20. Braun V, Clarke V. Using thematic analysis in psychology. Qualitative Res Psychol. 2006;3(2):77-101.

21. Morse JM. Confusing categorises and themes**.** Qualitative Health Res. 2008;18(6):727-728.

22. Birt L, Scott S, Cavers D, Campbell C, Walter F. Member checking: a tool to enhance trustworthiness or merely a nod to validation? Qualitat Health Res. 2016;26(13):1802-1811.

23. Persiani P, De Meo D, Giannini E, Calogero V, Speziale Varsamis T, Cavallo AU, Martini L, Cera G, Coluzzi F, Villani C. The aftermath of COVID-19 lockdown on daily life activities in orthopaedic patients. J Pain Res. 2021;14:575-583.

24. Baldwin D, Johnstone B, Ge B, Hewett J, Smith M, Sharp G. Randomized prospective study of a work place ergonomic intervention for individuals with rheumatoid arthritis and osteoarthritis. Arthritis Care Res. 2012;64:1527-1535.

25. Castro da Rocha FA, Melo LDP, Berenbaum F. Tackling osteoarthritis during COVID-19 pandemic. Ann Rheumat Dis. 2020. In Press. Online*:* doi: 10.1136/annrheumdis-2020-218372

26. Pinto AJ, Dunstan DW, Owen N, Bonfa E, Gualano B. Combating physical inactivity during the COVID-19 pandemic. Nat Rev Rheumatol. 2020;16:347–348.

27. Rogers NT, Waterlow NR, Brindle H, Enria L, Eggo RM, Lees S, et al. Behavioral change towards reduced intensity physical activity is disproportionately prevalent among adults with serious health issues or self-perception of high risk during the UK COVID-19 lockdown. Front Public Health. 2020;8:575091.

28. Valtorta NK, Kanaan M, Gilbody S, Ronzi S, Hanratty B. Loneliness and social isolation as risk factors for coronary heart disease and stroke: systematic review and meta-analysis of longitudinal observational studies. Heart. 2016;102(13):1009-1016.

29. Holt-Lunstad J, Smith TB. Loneliness and social isolation as risk factors for CVD: implications for evidence-based patient care and scientific inquiry. Heart. 2016;102(13):987-989.

30. Alpass FM, Neville S. Loneliness, health and depression in older males. Aging Mental Health. 2003;7(3):212-216.

For peer review only - http://bmjopen.bmj.com/site/about/guidelines.xhtml

31. Rafnsson SB, Orrell M, d'Orsi E, Hogervorst E, Steptoe A. Loneliness, social integration, and incident dementia over 6 years: prospective findings from the English Longitudinal Study of Ageing. J Gerontol B Psychol Sci Soc Sci2020; 75(1):114-124.

32. Steptoe A, Shankar A, Demakakos P, Wardle J. Social isolation, loneliness, and all-cause mortality in older men and women. Proc Natl Acad Sci USA. 2013;110(15):5797-5801.

33. Denzin NK. The research act: A theoretical introduction to sociological methods. New Jersey: Transaction Publishers, 1970.

34. Danielsson L, Kvarstein G, Bergvik S. Mediators of pain and physical function in female and male patients with chronic pain. J Pain Res. 2020;13:1059–1071.

35. Raisi-Estabragh Z, McCracken C, Bethell MS, Cooper J, Cooper C, Caulfield MJ, Munroe PB, Harvey NC, Petersen SE Greater risk of severe COVID- 19 in Black, Asian and Minority Ethnic populations is not explained by cardiometabolic, socioeconomic or behavioural factors, or by 25(OH)-vitamin D status: study of 1326 cases from the UK Biobank. J Public Health. 2020;42(3):451-460.

For peer review only

Table 1: Respondent characteristics and responses to health provision access from online survey at baseline

**Characteristics**		**Frequency (%)**
N		703
Gender	Female	574 (82.0)
	Male	126 (18.0)
	Non-binary	1 (0.1)
	Prefer to self-describe	1 (0.1)
	Prefer not to say	1 (0.1)
Age	18-30	21 (3.0)
	31-40	44 (6.3)
	41-50	113 (16.1)
	51-60	173 (24.6)
	61-70	203 (28.9)
	71-80	132 (18.8)
	80+	16 (2.3)
Region of respondent	England	660 (93.9)
	Scotland	21 (3.0)
	Wales	19 (2.7)
	Northern Ireland	2 (0.3)
Ethnic Group	White	682 (97.8)
	Mixed/Multiple ethnic group	4 (0.6)
	Asian	7 (1.0)
	Black, African, Caribbean	3 (0.4)
	Arab	1 (0.1)
Musculoskeletal (MSK) Diagnosis	Rheumatoid Arthritis	311 (44.2)
	Osteoarthritis	152 (21.6)
	Mechanical low back pain	70 (9.9)
	Fibromyalgia	68 (9.6)
	Psoriatic Arthritis	64 (9.1)
	Inflammatory Polyarthritis	63 (8.9)
	Hypermobility	40 (5.6)
	Specific MSK diagnosis not reported	33 (4.7)
	Connective tissue disease (e.g. Lupus, Scleroderma, Myositis)	25 (3.5)
	Ankylosing Spondylitis	22 (3.1)
	Osteoporosis	18 (2.5)
	Polymyalgia Rheumatica	10 (1.4)
	Ligament/Tendon Injury/Bursitis	9 (1.3)
	Neck pain	6 (0.8)
	Gout	5 (0.7)
	Other	63 (8.9)
How have your MSK symptoms been since the COVID measures started?	Decreased	45 (6.5)
	Stayed the same	287 (41.1)
	Increased	365 (52.4)
Pain (Scale: 0-10)	Mean (SD)	4.8 (2.6)
General Health (Scale: 0-10)	Mean (SD)	4.1 (2.4)
Total Lubben Social Network Score (Scale: 0-30)	Mean (SD)	14.8 (5.5)
Total UCLA Loneliness (Scale: 3-9)	Mean (SD)	5.1 (2.0)
Difficulty accessing medication	Yes	82 (11.7)
	No	616 (88.3)
Required someone to help access	Yes	309 (44.3)

For peer review only - http://bmjopen.bmj.com/site/about/guidelines.xhtml

medications	No	389 (55.7)
Changed medications since COVID-19 outbreak	Yes	103 (14.8)
	No	595 (85.2)
Required to seek advice from a health professional on condition	Yes	252 (36.1)
	No	446 (63.9)
Who did you contact	General Practitioner	158 (22.3)
	Practice Nurse/GP Nurse Practitioner	23 (3.2)
	Rheumatology Department	97 (13.7)
	Physiotherapy or Occupational Therapist	16 (2.3)
	Pharmacist	16 (2.3)
	Hospital Department (non-MSK)	10 (1.4)
	A&E	3 (0.4)
	Private chiropractor, osteopath or massage therapist	1 (0.1)
	Royal Osteoporosis Society	1 (0.1)
	Endocrinology Department	7 (1.0)
	Pain Clinic	2 (0.3)
	Counsellor or Health Psychologist	2 (0.3)
	Massage Therapist	1 (0.1)
	Podiatrist	1 (0.1)
	Nutritionist	1 (0.1)
	NHS 111	2 (0.3)
How easy has it been to get advice? (Scale: 0-10)	(Mean (SD) value. scale 0-10)	4.8 (3.3)

A&E- accident and emergency; CI – confidence intervals; JIA – Juvenile Inflammatory Arthritis; MSK – musculoskeletal; NHS – National Health Service; SD – standard deviation; UCLA – University of California Los Angeles Loneliness Score

For peer review only

Table 2: Association at baseline between musculoskeletal (MSK) symptoms and selected participant characteristics and questionnaire responses.

	"How have your symptoms been since the current COVID measures started?"			**Chi-squared test of association with outcome (symptoms)**
	**Decreased**	**Stable**	**Increased**	p-value
n (%)	45 (6)	287 (41)	365 (52)	
Age group				p=0.017
18-60	22 (6)	125 (36)	200 (58)	
60 plus	33 (7)	162 (46)	165 (47)	
Gender				p=0.11
Male	8 (7)	60 (49)	54 (44)	
Female	37 (6)	224 (39)	311 (54)	
MSK diagnosis*				p<0.001
RA	17 (5)	155 (48)	149 (46)	
IA	4 (10)	24 (60)	12 (30)	
PsA	4 (6)	22 (34)	38 (59)	
Other	19 (7)	83 (31)	166 (62)	
Situation				p=0.880
At home	24 (7)	141 (40)	191 (54)	
Self-isolating	8 (7)	49 (40)	64 (53)	
Shielding	13 (6)	97 (44)	110 (50)	
Difficulty accessing medication?				p=0.103
Yes	4 (5)	26 (32)	52 (63)	
No	41 (7)	261 (42)	313 (51)	
Change medication?				p<0.001
Yes	9 (9)	21 (20)	73 (71)	
No	36 (6)	266 (45)	292 (49)	
Consult health professional?				p<0.001
Yes	15 (6)	64 (25)	172 (69)	
No	30 (7)	223 (50)	193 (43)	
Physical activity				p<0.001
Decreased	31 (7)	141 (30)	302 (64)	
Same	3 (3)	89 (75)	26 (22)	
Increased	11 (10)	57 (54)	37 (35)	

Data are frequency (%) unless stated otherwise

* RA=Rheumatoid arthritis, IA=Inflammatory arthritis, PsA=Psoriatic arthritis, Other=mainly Osteoarthritis (55%) but also including any diagnosis that wasn't RA, IA or PsA (see **Table 1**)

For peer review only

Table 3: Interview sub-sample characteristics (N=26)

Characteristics		Frequency
**Gender**	Female	17
	Male	9
**Age**	18-30	1
	31-40	4
	41-50	5
	51-60	4
	61-70	3
	71-80	8
	80+	1
**Ethnic Group**	White	26
**Musculoskeletal (MSK) Diagnosis**	Rheumatoid Arthritis	18
	Psoriatic Arthritis	5
	Inflammatory Polyarthritis	1
	Polymyalgia Rheumatica	2
**How have your MSK symptoms been since the COVID measures started? (reported at baseline)**	Decreased	3
	Stayed the same	9
	Increased	14

For peer review only

For peer review only - http://bmjopen.bmj.com/site/about/guidelines.xhtml

Table 4: Key themes generated from the qualitative study - perceived underlying attributions for symptom change

Experience of symptoms	Attributions from interview data	Representative quote
**Increased symptoms**	**Accessing healthcare**<br>Holding on to concerns	*I don't like to worry them, they've got enough to do … It's something that I feel I can manage until when this is all over hopefully I'll get an appointment to go in and I'll mention it then.* [MSK17 female age 70-74]
	Postponement or cancelation of appointments, treatments and investigations	*I think that's something I feel like I've missed out on with the lockdown is, I would actually quite have liked to have some proper physio, especially on the knees and that. Because the swelling just doesn't seem to want to go, as much as I poke and prod it and things like that.* [MSK 10 female age 50-59]<br><br>*It was planned for me to have an MRI scan on my neck, but then this all happened and it's just sort gone out the window…. It's quite hard, day-to-day, with the pain levels in my neck, but under the current situation, I would rather put up with the pain than put myself at risk by going into a hospital.* [MSK20 male age 50-55]
	Telephone appointments	*Where I've missed out because of the lockdown, is that although when I phoned the rheumatology department they've been really helpful, it's not like having proper management. So all of the questions that I would normally have asked, I don't seem to ask so much on the phone... It would be nice to say to somebody, "This bit hurts, what can I do about it? Is this any good?" and things like that. But you need to have the physical and visual side of that.* [MSK 10 female age 50-54]
	**Reduced physical activity**<br>Reduced planned exercise (including less opportunity or access to facilities to exercise)	*I think I'm so much less active, before all this I was going swimming three times a week, that was the best exercise for me really, because it didn't put as much pressure on my body as going to the gym, running or anything like that. And, I don't think I fully appreciated how much swimming helped, whereas now, obviously, I'm not doing anything and I am just only getting out of the house for a little bit once a week, I've just found everywhere has just stiffened up so much…It's a bit scary, really.* [MSK5 female 25-29]<br><br>*[My joints are] getting stiff because you don't go out much to get exercise…. sitting down a lot makes your hips an your back ache and don't get up often where your chair is*. [MSK6 male 70-74]
	Reduced routine activity (e.g. walking to shops, going to work)	*I've been finding, like, sitting at my desk all day for work, I do get quite sore and quite stiff after work, because I think maybe when I was in an office environment I would be going out at lunchtime or just walking around the office to talk to different people…generally if I'm not sat at my desk working, I suppose I'm sat on the sofa, I'm not really that mobile around the house.* [MSK5 female 25-29]<br><br>*[Pain and stiffness] have been a bit more apparent because I haven't been able to get out as much and use my joints. I find when I've been cooped up in a space and sitting down for a while, then obviously it affects you more. I can walk to work and back again and it frees things up a little bit. But obviously not being able to do that, it's not the same… it gets worse when you can't be as mobile as you could be.* [MSK12 male 35-39]
	**Working at home**<br>Lack of ergonomic work-space	*At work I have a Varidesk…so, I can stand up. I also have like a proper chair, like a medical chair… [Working at home] was horrendous because I was working off my lap on a laptop, bending over. So, my back and my shoulder was very, very painful.* [MSK2 female 35-39]

For peer review only - http://bmjopen.bmj.com/site/about/guidelines.xhtml

	Struggling with household tasks	*I used to have someone come on a Thursday and do my chores for me…So perhaps it's something like that as well, you know, sort of without the help there…. I think with the loss of energy you get more of a hurting in the legs at night and that.* [MSK19 female 70-74]
	**Wellbeing** Managing multiple roles within the home (Home-schooling, childcare and work)	*I probably went way too hard on myself, trying to plan lessons for [children]. You're also then working, you're also trying to run the house… completely overdid it……I do think the flare that I had was related to being in lockdown. I'm not sure I would have had it that bad if we were out of lockdown, because I would have been able to maybe adjust working life, the girls would have been at school, or my parents would have had them…. It was definitely probably reactive from overdoing it… trying to juggle so many plates.* [MSK13 female 30-34]
	Anxiety or low mood associated with the pandemic and being in a clinically vulnerable group.	*I do struggle quite a lot with my mental health and I've found that my physical health has got so much worse, as well… it's definitely harder at the moment.* [MSK5 female 25-29]<br><br>*Currently, I've got swelling ….and I've got pain in multiple areas. So, I would say I'm having a flare up…maybe it's the stress had an impact on my immune system.* [MSK2 female 35-39]
**Stable symptoms**	Maintaining exercise	*In terms of my arthritis and stuff, that's not been – touch wood – too bad. I've tried to keep on top of it by trying to stay as active as I can really… I've been probably doing 2K, 3K, 5Ks a week and then us, as a family unit, we try and go for a walk…so the pain has not been too bad.* [MSK14-male-35-39]
	Pre-existing stable or well-controlled condition	*I don't think there's a lot of difference really, it's quite well controlled anyway.* [MSK4 female 65-69]
**Decreased symptoms**	An opportunity for respite, improved wellbeing	*The arthritis was getting worse [and since lockdown] I've had no flare ups. It's all been very much under control, and I think that's partly being removed from the work situation but it's partly having that easier pace of life, and if my body needs to sleep there's not an alarm set, it just sleeps. I think before this I was feeling very tired and unwell….I now feel far calmer, physically rested, which helps no end.* [MSK21 female 45-49]
	More opportunity for exercise	*I'm actually feeling really good right now….I like to think it's the exercises…I've definitely done more daily exercises with my son and skipping and stuff and more bike rides. I like cycling anyway, but I've been on more long bike rides and probably been taking the dog for a walk probably more than I'd done before.* [MSK11 female 50-54]

For peer review only

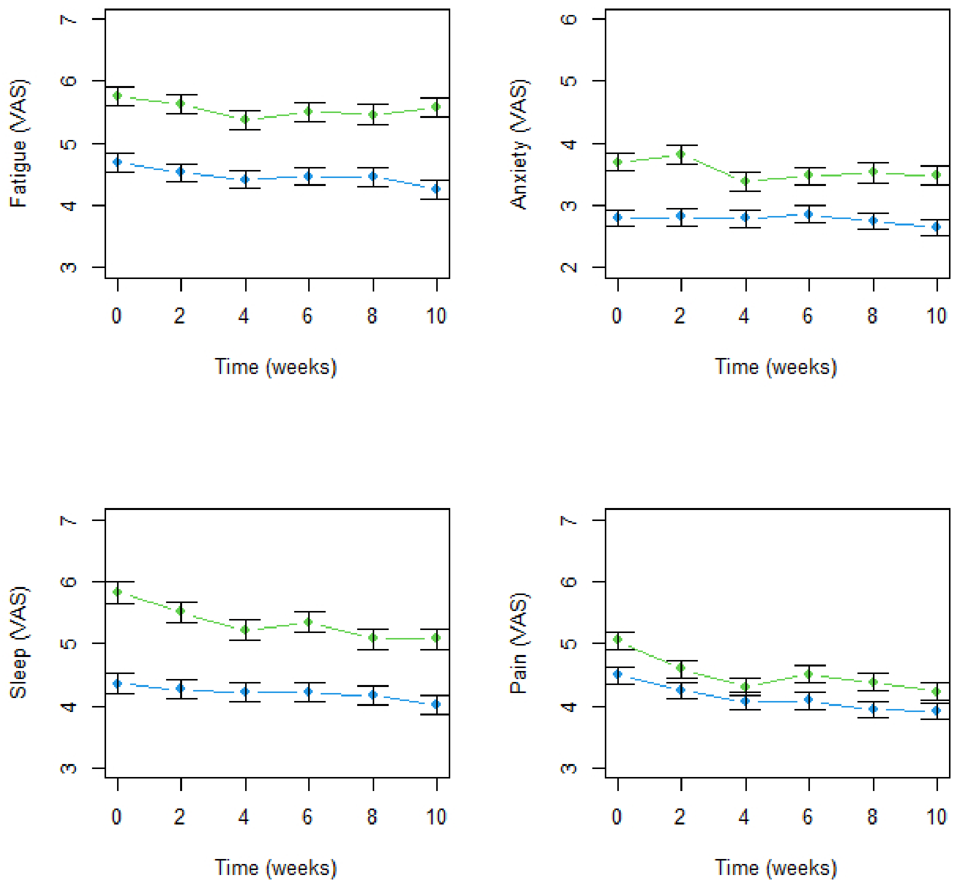

Figure 1: Change in symptoms from baseline over the 10-week follow-up interval.

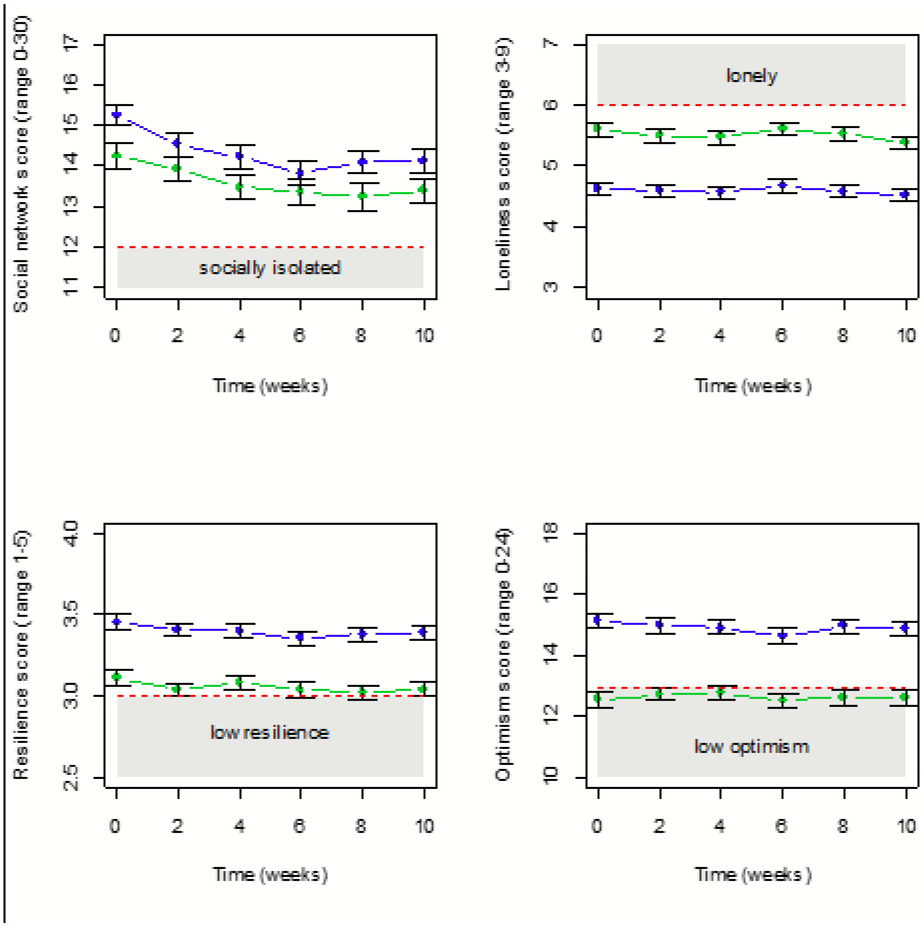

Figure 2: Change in behaviour outcomes from baseline over the 10-week follow-up interval.

Supplementary File 1: Online survey (baseline)

The impact of COVID-19 self-isolation measures on people with musculoskeletal diseases

Questionnaire

Version 0.1 - dated: 19 April 2020

Information to help you decide if you want to take part in this research

You are being invited to take part in a research project so we can understand how you are managing self-isolation during the coronavirus pandemic (Covid-19). We are particular interested in your joint pain, wellbeing and social contacts. Please ensure you have read and understood this information before continuing.

What is this project about?

This study aims to identify how the Covid-19 pandemic is impacting on the health and wellbeing of people with bone, joint or muscle pain (musculoskeletal disease). It aims to better understand how the current Covid-19 self-isolation is affecting people, their experiences of isolating and how they are managing their joint pain during this time. It will tell us about the effects of social isolation measures and changes in health services on people's physical health and wellbeing. The findings will be used to help understand who is at greatest risk of poor health and wellbeing during self-isolation so we can better support them during this outbreak.

Why have I been asked?

You have been sent this survey or have accessed it via social media. The project is entirely voluntary. You do not have to be in isolation to take part.

What will taking part involve?

You will be asked to complete a 15-minute survey online. Then every 2 weeks for the next 12 weeks, we will ask you to complete the same survey again. The whole study will last for about 12 weeks while these social isolation measures are in place.

What will you ask and what will happen to the information I give you?

You will be asked questions about yourself, your current joint pain, your medication and health care requirements for joint pain, and some questions on your physical health and wellbeing. We will also ask you whether you are isolating, some questions on social isolation, loneliness and resilience to this change in lifestyle, and some questions on your arthritis symptoms. Some of these questions are considered sensitive data, such as questions on your wellbeing. No information provided will be passed to any third parties. Your data will then be analysed by researchers from UEA and will be published in scientific papers and used to inform advice given during Covid-19. Nobody will be able to identify you from any materials we publish or present from this study.

You will only be identified to the research team members by your email address. We need this so that we can send you the follow-up fortnightly survey links for the next 12 weeks. We will

not use your email address for any other purpose. We will not share this email address with any other people or organisations.

This study has received approval from the University of East Anglia's Faculty of Medicine and Health Science's Research Ethics Committee.

How long will my data be stored for?

Your data will only be used for this study and not shared. Your data will be stored for 10 years by UEA after the end of the research. At this point the data will be reviewed, and if they are still deemed to be of public interest, they may be retained for longer.

How do I find out the results?

Researchers at UEA will provide a summary of the findings online through the UEA website. We will provide a hyperlink to all participants (via their email address) of this once the data has been analysed and reported.

Concerns

If you have any concerns about the study, you can contact the UEA study team at 01603 597205 or email noar@uea.ac.uk. If you feel your concerns have not been handled satisfactorily, you can contact the Dean of the Norwich Medical School (Professor William Fraser) at: W.Fraser@uea.ac.uk

Consent

By taking part, you are agreeing that you have read and understood the information above about the study. If you have any questions or concerns please contact 01603 597205 before agreeing to take part and completing the survey.

I understand that:

- My participation is completely voluntary.
- The data gathered in this study will be stored securely and it will not be possible to identify me in any outputs from this research.
- Only UEA research staff, will have access to the data.

I consent by ticking this box: ☐

Survey

Date of birth __ __ / __ __ / __ __ __ __

Date completed __ __ / __ __ / __ __ __ __

Email Address (to send the follow-up surveys) ☐

Female ☐

Male ☐

Age (years) ☐

What is was/your occupation:______________________________

Are you currently:

a. Working now ☐
b. Unemployed ☐
c. Off sick ☐
d. Retired ☐
e. Not working – health grounds ☐
f. Never worked ☐
g. Homekeeper/parent/carer ☐
h. Student ☐
i. Working reduced hours ☐
j. Working, but there has been a change in duties ☐
k. Maternity/Paternity leave ☐

	YES	NO
**Are you currently off work because of coronavirus self-isolation?**		
**Are you currently working from home because of coronavirus self-isolation?**		

For peer review only

Which category best describes your ethnic group or background. (Tick only one box)

White	☐
Black, Caribbean	☐
Black, African	☐
Any other Black background	☐
Indian	☐
Pakistani	☐
Bangladeshi	☐
Any other Asian background	☐
Chinese	☐
White and Black Caribbean	☐
White and Black African	☐
White and Asian	☐
Any other mixed background	☐
Any other ethnic group	☐

For peer review only

Your Bone, Joint and Muscle Disease

What is your musculoskeletal disease diagnosis? (Please tick all relevant boxes)

Rheumatoid Arthritis (RA)	
Osteoarthritis (OA)	
Fibromyalgia	
Ankylosing Spondylitis	
Psoriatic Arthritis (PsA)	
Inflammatory Polyarthritis (IP)	
Mechanical Low Back Pain	
Other (please specify) ____________	
Don't Know	

Where do you feel your pain?

	Left	Right
Hip		
Knee		
Ankle		
Foot		
Shoulder		
Elbow		
Wrist		
Hand		
Neck		
Back (mid or low back)		

For peer review only

General Health Questions

1. Have you had any of the following conditions?

	YES	NO
Heart condition (such as heart failure or past heart attack)		
High blood pressure		
Stroke or TIA (mini stroke/transient visual loss)		
Breathing condition (such as asthma, emphysema, COPD)		
Diabetes		
Stomach Ulcer		
Liver problems		
Kidney problems		
Cancer (except skin cancer)		
Depression or Anxiety		
Glaucoma		
Dementia		
Epilepsy		

For peer review only

Changes since self-isolation

Are you self-isolated for COVID-19 (i.e. not leaving home for activities other than shopping, exercise once daily or essential tasks)?

❑ Yes ❑ No

Have you had difficulty accessing medications?

❑ Yes ❑ No

Have you needed someone to help to access your medications?

❑ Yes ❑ No

Have you had to change your medications since the COVID-19 pandemic started?

❑ Yes ❑ No

Have you needed to seek advice from a health professional on your condition?

❑ Yes ❑ No

Has your normal level of physical activity changed since the COVID self isolation started?

❑ Decreased ❑ Stayed same ❑ Increased

How has your joint pain been overall since the COVID self isolation started?

❑ Decreased ❑ Stayed same ❑ Increased

How have your levels of energy been since the COVID self isolation started?

❑ Decreased ❑ Stayed same ❑ Increased

For peer review only

Clinical Health Assessment Questionnaire (CLINHAQ)

We are interested in learning how your illness affects your ability to function in daily life.

Please tick the response which best describes your usual abilities OVER THE PAST WEEK:

	Without any difficulty	With some difficulty	With much difficulty	Unable to do
DRESSING & GROOMING: Are you able to:				
Dress yourself, including tying shoelaces & doing buttons?	_______	_______	_______	_______
Shampoo your hair?	_______	_______	_______	_______
RISING: Are you able to:				
Stand up from an armless straight chair?	_______	_______	_______	_______
Get in and out of bed?	_______	_______	_______	_______
EATING: Are you able to:				
Cut your meat?	_______	_______	_______	_______
Lift a full cup or glass to your mouth?	_______	_______	_______	_______
Open a new carton of milk (or soap powder)?	_______	_______	_______	_______
WALKING: Are you able to:				
Walk outdoors on flat ground?	_______	_______	_______	_______
Climb up five steps?	_______	_______	_______	_______

Please tick any AIDS or DEVICES that you usually use for any of these activities:

______ Cane (W) ______ Walking frame (W) ______ Built up or special utensils (E)

______ Crutches (W) ______ Wheelchair (W) ______ Special or built up chair (A)

______ Devices used for dressing (button hook, zipper pull, long handled shoe horn) (D)

______ Other, specify ____

Please tick any category for which you usually need HELP FROM ANOTHER PERSON:

________ Dressing & grooming ________ Eating

________ Rising ________ Walking

For peer review only

We are also interested in learning whether or not you are affected by pain because of your illness.

How much pain have you had because of your arthritis IN THE PAST WEEK?

Place a mark on the line to indicate the severity of the pain

No pain Severe pain

0 10 20 30 40 50 60 70 80 90 100

How much trouble have you had with your stomach (ie nausea, heartburn, bloating, pain, etc)

IN THE PAST WEEK? Place a mark on the line below to indicate the severity of the problem:

No stomach problem Severe stomach problem

0 10 20 30 40 50 60 70 80 90 100

Please tick the response which best describes your usual abilities OVER THE PAST WEEK:

	Without any difficulty	With some difficulty	With much difficulty	Unable to do
HYGIENE: Are you able to:				
Wash and dry your entire body?	________	________	________	________
Take a bath?	________	________	________	________
Get on and off the toilet?	________	________	________	________
REACH: Are you able to:				
Reach and get down a 5lb object (eg a bag of potatoes) from just above your head?	________	________	________	________
Bend down to pick up clothing from the floor?	________	________	________	________
GRIP: Are you able to:				
Open car doors?	________	________	________	________
Open jars which have been previously opened?	________	________	________	________
Turn taps on and off?	________	________	________	________
ACTIVITIES: Are you able to:				
Run errands and shop?	________	________	________	________
Get in and out of a car?	________	________	________	________
Do chores (vacuuming, housework or light gardening)?	________	________	________	________

Please tick any AIDS OR DEVICES that you usually use for any of these activities:

________ Bath rail (H) ________ Bath seat (H)

________ Raised toilet seat (H) ________ Jar opener (for jars previously opened) (G)

________ Long-handled appliances for reach (R) ________ Other (specify) ________________________

Please tick any categories for which you usually need HELP FROM ANOTHER PERSON:

________ Hygiene ________ Gripping and opening things

________ Reach ________ Errands and chores

In general, would you say that your HEALTH IS:

________ Excellent ________ Good ________ Fair ________Poor

Consider ALL THE WAYS THAT YOUR ARTHRITIS AFFECTS YOU. RATE HOW YOU ARE DOING on the following scale by placing a mark on the line below:

Very well Very poorly

0 10 20 30 40 50 60 70 80 90 100

We are interested in knowing about any problems that you may have been having with fatigue.

How much of a problem has fatigue or tiredness been for you IN THE PAST WEEK? Place a mark on the line below:

Fatigue is no problem Fatigue is a major problem

0 10 20 30 40 50 60 70 80 90 100

For peer review only

How much of a problem has sleep (ie resting at night) been for you IN THE PAST WEEK? Place a mark on the line below:

Sleep is no problem Sleep is a major problem

0 10 20 30 40 50 60 70 80 90 100

For peer review only

Lubben Social Network Scale-6

The following 6 questions are about how often you are in contact with your family and friends. Please **tick** the box to best represent your answer for each question.

	None	1	2	3 or 4	5 to 8	9 or more
Family – considering the people to whom you are related by birth, marriage, adoption etc…						
How many relatives do you see or hear from at least once a month?						
How many relatives do you feel at ease with that you can talk about private matters?						
How many relatives do you feel close to such that you could call on them for help?						
Considering all of your friends, including those who live in your neighbourhood						
How many of your friends do you see or hear from at least once a month?						
How many friends do you feel at ease with that you can talk about private matters?						
How many friends do you feel close to such that you could call on them for help?						

University of California, Los Angeles (UCLA) three-item loneliness scale

The following 3 questions are about how often you feel lonely. Please **tick** the box to best represent your answer for each question.

	Hardly Ever	Some of the Time	Often
How often do you feel that you lack companionship?			
How often do you feel left out?			
How often do you feel isolated from others?			

6-item Brief Resilience Scale

The following 6 statement relate to how 'resilient' you feel. Please respond to each item by marking one box per row.

	Strongly Disagree	Disagree	Neutral	Agree	Strongly Agree

For peer review only

I tend to bounce back quickly after hard times					
I have a hard time making it through stressful events					
It does not take me long to recover from a stressful events					
It is hard for me to snap back when something wrong happens					
I usually come through difficult times with little trouble					
I tend to take a long time to get over setbacks in my life					

Revised Life Orientation Test (LOT-R)

Please answer the following questions about yourself. Be as honest as you can throughout and try not to let your responses to one question influence your response to other questions. There are no right or wrong answers.

	Strongly Disagree	Disagree	Neutral	Agree	Strongly Agree
In uncertain times, I usually expect the best					
It's easy for me to relax					
If something can go wrong for me, it will					
I'm always optimistic about my future					
I enjoy my friends a lot					
It's important for me to keep busy					
I hardly ever expect things to go my way					
I don't get upset too easily					
I rarely count on good things happening to me					
Overall, I expect more good things to happen to me than bad					

We would like to thank you for completing these questionnaires.
Your contribution is much appreciated.
If you have any questions or queries about this form, please contact:
NOAR Office Tel: (01603) 597204/5
Email: noar@uea.ac.uk

For peer review only

Supplementary File 2: Timeline of study activity against UK legislation and COVID policy implementation.

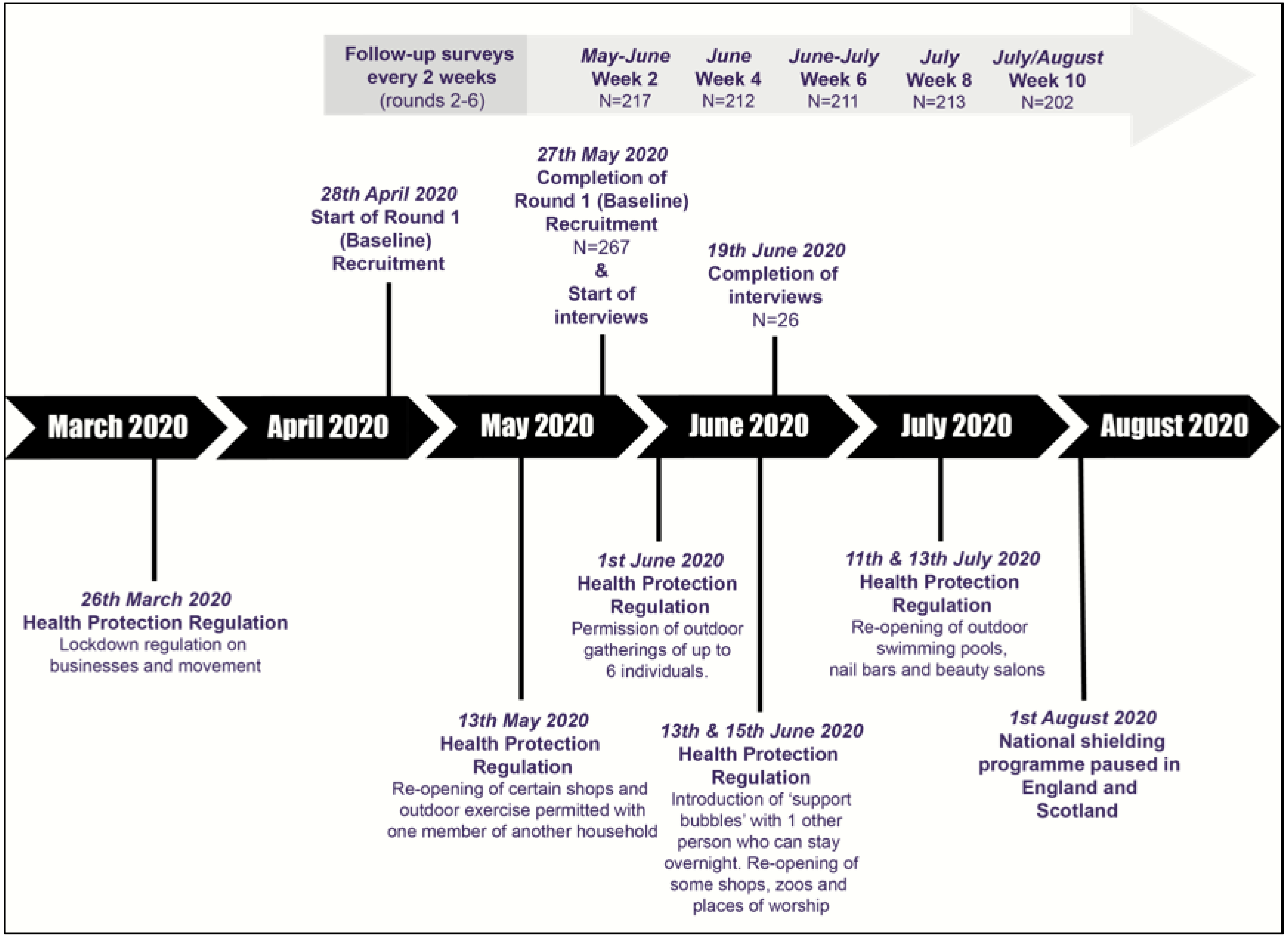

Supplementary File 3: Interview topic guide

1 **How is everyday life for you at the moment?**

Prompts:

- Getting out the house
- Keeping in touch with people
- Changes in routines
- Changes in mood
- Changes in health
- Any adaptions made

2 **How is your joint pain?**

Prompts

- Recent changes and what they think is the cause
- Fatigue
- Medication
- Exercise
- Self help
- Contact with health professionals

3 **How are you managing daily tasks?**

Prompts

- Do you usually have help shopping, cleaning
- Have you made changes to the ways you do things

3a How do you feel about these changes?

4 **How have your social activities changed?**

Prompts

- Keeping in touch with family and friends
- Activities in the community i.e. clubs, quizzes crafts, pub
- Using social media
- Accessing support groups
- New social contacts

4a How do you feel about these changes?

5 **Is there anything else you would like to tell us about your life during this period?**

For peer review only

Supplementary File 4: Characteristics of participants at baseline and Week 10

	Timepoint		P-value (chi-squared)
	**Baseline**	**Week 10**	
N (% of baseline)	703 (100)	490 (70)	
**Age group**			p=0.022
18-60	351 (50)	212 (43)	
60 plus	351 (50)	278 (57)	
**Gender**			p=0.804
Male	574 (82)	402 (83)	
Female	126 (18)	85 (17)	
**MSK Diagnosis**			p=0.632
RA	321 (46)	241 (48)	
IA	41 (6)	29 (6)	
PsA	65 (9)	48 (9)	
Other	269 (39)	171 (37)	

Data are frequency (%); MSK – Musculoskeletal; RA - Rheumatoid arthritis, IA - Inflammatory arthritis; PsA - Psoriatic arthritis; Other - mainly Osteoarthritis (55%) but also including any diagnosis that was not RA, IA or PsA (see **Table 1**)

For peer review only

Supplementary File 5: Interpretive synthesis of quantitative and qualitative findings

**Survey Findings (See Tables 1-2)**	**Interview Findings (See Tables 3-4)**
People with musculoskeletal disease reported an increase in pain and symptoms	*Attributions from symptom increase:*<br>- Changes in access to healthcare<br>- Reduced physical activity<br>- Ergonimics of working at home<br>- Reduced wellbeing<br>- Increased demands at home
Those who reported decreased physical activity were more likely to report increased symptoms	*Increase in symptoms attributed to:*<br>- Reduced routine activitiy<br>- Reduced opportunities for exercise
Increase in symptoms was significantly worse for those aged 18-60 years of age in comparison to older respondents	*Changes to daily life described by working age people included:*<br>- Disrupted daily routines<br>- Managing multiple roles at home, including childcare<br>- Unsuitable home working environment
Over the 10 weeks, greater social isolation, loneliness and reduced optimism were reported	*Feelings of low mood, boredom, social isolation or anxiety were related to:*<br>- Experience of restrictive measures<br>- Being in a clinically vulnerable group<br>- Disease symptoms<br>- A sense of being forgotten about
Half of those (53%) with increased symptoms at baseline had not consulted a healthcare professional	*Disrupted access to healthcare:*<br>- 'Holding on to concerns', wary of burdening healthcare staff, or uncertainty around service availability<br>- Reservations about telephone consultations<br>- Appointments, treatments and investigations postponed or cancelled

STROBE Statement—checklist of items that should be included in reports of observational studies

	Item No	Recommendation	Page No
**Title and abstract**	1	(*a*) Indicate the study's design with a commonly used term in the title or the abstract	Title Page
		(*b*) Provide in the abstract an informative and balanced summary of what was done and what was found	Abstract
**Introduction**			
Background/rationale	2	Explain the scientific background and rationale for the investigation being reported	Intro Para 1-3
Objectives	3	State specific objectives, including any prespecified hypotheses	Intro Para 4
**Methods**			
Study design	4	Present key elements of study design early in the paper	Methods Para 1
Setting	5	Describe the setting, locations, and relevant dates, including periods of recruitment, exposure, follow-up, and data collection	Methods Para 2-4, 9
Participants	6	(*a*) *Cohort study*—Give the eligibility criteria, and the sources and methods of selection of participants. Describe methods of follow-up<br>*Case-control study*—Give the eligibility criteria, and the sources and methods of case ascertainment and control selection. Give the rationale for the choice of cases and controls<br>*Cross-sectional study*—Give the eligibility criteria, and the sources and methods of selection of participants	Methods, Para 2, 8
		(*b*) *Cohort study*—For matched studies, give matching criteria and number of exposed and unexposed<br>*Case-control study*—For matched studies, give matching criteria and the number of controls per case	Not applicable
Variables	7	Clearly define all outcomes, exposures, predictors, potential confounders, and effect modifiers. Give diagnostic criteria, if applicable	Methods Para 4,6
Data sources/ measurement	8*	For each variable of interest, give sources of data and details of methods of assessment (measurement). Describe comparability of assessment methods if there is more than one group	Methods Para 4
Bias	9	Describe any efforts to address potential sources of bias	Discussion Para 5,6
Study size	10	Explain how the study size was arrived at	Methods, Para 3, 6, 10
Quantitative variables	11	Explain how quantitative variables were handled in the analyses. If applicable, describe which groupings were chosen and why	Methods, Para 7, 8
Statistical methods	12	(*a*) Describe all statistical methods, including those used to control for confounding	Methods, Para 7, 8
		(*b*) Describe any methods used to examine subgroups and interactions	Methods, Para 8
		(*c*) Explain how missing data were addressed	Methods Para 8
		(*d*) *Cohort study*—If applicable, explain how loss to follow-up was addressed	Not applicable

For peer review only

		Case-control study—If applicable, explain how matching of cases and controls was addressed<br>*Cross-sectional study*—If applicable, describe analytical methods taking account of sampling strategy	
		(*e*) Describe any sensitivity analyses	Methods Para 8
**Results**			
Participants	13*	(a) Report numbers of individuals at each stage of study—eg numbers potentially eligible, examined for eligibility, confirmed eligible, included in the study, completing follow-up, and analysed	Results, Para 1
		(b) Give reasons for non-participation at each stage	Results, Para 1
		(c) Consider use of a flow diagram	Considered but not prioritised
Descriptive data	14*	(a) Give characteristics of study participants (eg demographic, clinical, social) and information on exposures and potential confounders	Results, Para 1, Table 1
		(b) Indicate number of participants with missing data for each variable of interest	Results, Para 1, Table 1-3
		(c) *Cohort study*—Summarise follow-up time (eg, average and total amount)	Methods, Para 6
Outcome data	15*	*Cohort study*—Report numbers of outcome events or summary measures over time	Results, Para 1, Table 1-3
		Case-control study—Report numbers in each exposure category, or summary measures of exposure	No CC study
		Cross-sectional study—Report numbers of outcome events or summary measures	Not cross-sectional
Main results	16	(*a*) Give unadjusted estimates and, if applicable, confounder-adjusted estimates and their precision (eg, 95% confidence interval). Make clear which confounders were adjusted for and why they were included	Table 2
		(*b*) Report category boundaries when continuous variables were categorized	Methods, Para 8
		(*c*) If relevant, consider translating estimates of relative risk into absolute risk for a meaningful time period	Not applicable
Other analyses	17	Report other analyses done—eg analyses of subgroups and interactions, and sensitivity analyses	Results, Table 3
**Discussion**			
Key results	18	Summarise key results with reference to study objectives	Discussion Para 1
Limitations	19	Discuss limitations of the study, taking into account sources of potential bias or imprecision. Discuss both direction and magnitude of any potential bias	Discussion Para 5,6
Interpretation	20	Give a cautious overall interpretation of results considering objectives, limitations, multiplicity of analyses, results from similar studies, and other relevant evidence	Conclusion Para 1

For peer review only

For peer review only - http://bmjopen.bmj.com/site/about/guidelines.xhtml

Generalisability	21	Discuss the generalisability (external validity) of the study results	Discussion Para 5,6
**Other information**			
Funding	22	Give the source of funding and the role of the funders for the present study and, if applicable, for the original study on which the present article is based	Declarations

*Give information separately for cases and controls in case-control studies and, if applicable, for exposed and unexposed groups in cohort and cross-sectional studies.

Note: An Explanation and Elaboration article discusses each checklist item and gives methodological background and published examples of transparent reporting. The STROBE checklist is best used in conjunction with this article (freely available on the Web sites of PLoS Medicine at http://www.plosmedicine.org/, Annals of Internal Medicine at http://www.annals.org/, and Epidemiology at http://www.epidem.com/). Information on the STROBE Initiative is available at www.strobe-statement.org.

For peer review only

COREQ (COnsolidated criteria for REporting Qualitative research) Checklist

A checklist of items that should be included in reports of qualitative research. You must report the page number in your manuscript where you consider each of the items listed in this checklist. If you have not included this information, either revise your manuscript accordingly before submitting or note N/A.

Topic	Item No.	Guide Questions/Description	Reported on Page No.
**Domain 1: Research team and reflexivity**			
Personal characteristics			
Interviewer/facilitator	1	Which author/s conducted the interview or focus group?	
Credentials	2	What were the researcher's credentials? E.g. PhD, MD	
Occupation	3	What was their occupation at the time of the study?	
Gender	4	Was the researcher male or female?	
Experience and training	5	What experience or training did the researcher have?	
Relationship with participants			
Relationship established	6	Was a relationship established prior to study commencement?	
Participant knowledge of the interviewer	7	What did the participants know about the researcher? e.g. personal goals, reasons for doing the research	
Interviewer characteristics	8	What characteristics were reported about the inter viewer/facilitator? e.g. Bias, assumptions, reasons and interests in the research topic	
**Domain 2: Study design**			
Theoretical framework			
Methodological orientation and Theory	9	What methodological orientation was stated to underpin the study? e.g. grounded theory, discourse analysis, ethnography, phenomenology, content analysis	
Participant selection			
Sampling	10	How were participants selected? e.g. purposive, convenience, consecutive, snowball	
Method of approach	11	How were participants approached? e.g. face-to-face, telephone, mail, email	
Sample size	12	How many participants were in the study?	
Non-participation	13	How many people refused to participate or dropped out? Reasons?	
Setting			
Setting of data collection	14	Where was the data collected? e.g. home, clinic, workplace	
Presence of non-participants	15	Was anyone else present besides the participants and researchers?	
Description of sample	16	What are the important characteristics of the sample? e.g. demographic data, date	
Data collection			
Interview guide	17	Were questions, prompts, guides provided by the authors? Was it pilot tested?	
Repeat interviews	18	Were repeat inter views carried out? If yes, how many?	
Audio/visual recording	19	Did the research use audio or visual recording to collect the data?	
Field notes	20	Were field notes made during and/or after the inter view or focus group?	
Duration	21	What was the duration of the inter views or focus group?	
Data saturation	22	Was data saturation discussed?	
Transcripts returned	23	Were transcripts returned to participants for comment and/or	

For peer review only

Topic	Item No.	Guide Questions/Description	Reported on Page No.
		correction?	
**Domain 3: analysis and findings**			
Data analysis			
Number of data coders	24	How many data coders coded the data?	
Description of the coding tree	25	Did authors provide a description of the coding tree?	
Derivation of themes	26	Were themes identified in advance or derived from the data?	
Software	27	What software, if applicable, was used to manage the data?	
Participant checking	28	Did participants provide feedback on the findings?	
Reporting			
Quotations presented	29	Were participant quotations presented to illustrate the themes/findings? Was each quotation identified? e.g. participant number	
Data and findings consistent	30	Was there consistency between the data presented and the findings?	
Clarity of major themes	31	Were major themes clearly presented in the findings?	
Clarity of minor themes	32	Is there a description of diverse cases or discussion of minor themes?	

Developed from: Tong A, Sainsbury P, Craig J. Consolidated criteria for reporting qualitative research (COREQ): a 32-item checklist for interviews and focus groups. *International Journal for Quality in Health Care*. 2007. Volume 19, Number 6: pp. 349 – 357

Once you have completed this checklist, please save a copy and upload it as part of your submission. DO NOT include this checklist as part of the main manuscript document. It must be uploaded as a separate file.

For peer review only

The impact of COVID-19 pandemic social restriction measures on people with rheumatic and musculoskeletal diseases in the UK: a mixed-methods study.

Journal:	*BMJ Open*
Manuscript ID	bmjopen-2021-048772.R2
Article Type:	Original research
Date Submitted by the Author:	13-May-2021

Complete List of Authors:	Smith, Toby O.; University of Oxford, Nuffield Department of Orthopaedics, Rheumatology and Musculoskeletal Sciences; University of East Anglia,<br>Belderson, P; University of East Anglia<br>Dainty, Jack; University of East Anglia Faculty of Medicine and Health Sciences<br>Birt, Linda; University of East Anglia, School of Health Sciences<br>Durrant, K; University of East Anglia<br>Chipping, JR; University of East Anglia<br>Tsigarides, J; University of East Anglia, Rheumatology Department; Norfolk and Norwich University Hospital, Rheumatology Department<br>Yates, M; University of East Anglia, Rheumatology Department; Norfolk and Norwich University Hospital, Rheumatology Department<br>Naughton, Felix; University of East Anglia Faculty of Medicine and Health Sciences, School of Health Sciences; University of East Anglia<br>Werry, S<br>Notley, Caitlin; University of East Anglia, Norwich Medical School<br>Shepstone, Lee; University of East Anglia, Norwich Medical School<br>MacGregor, Alex; University of East Anglia Faculty of Medicine and Health Sciences, Norwich Medical School

**Primary Subject Heading**:	Rheumatology
Secondary Subject Heading:	Epidemiology
Keywords:	Rheumatology < INTERNAL MEDICINE, Pain management < ANAESTHETICS, COVID-19, Musculoskeletal disorders < ORTHOPAEDIC & TRAUMA SURGERY

SCHOLARONE™
Manuscripts

I, the Submitting Author has the right to grant and does grant on behalf of all authors of the Work (as defined in the below author licence), an exclusive licence and/or a non-exclusive licence for contributions from authors who are: i) UK Crown employees; ii) where BMJ has agreed a CC-BY licence shall apply, and/or iii) in accordance with the terms applicable for US Federal Government officers or employees acting as part of their official duties; on a worldwide, perpetual, irrevocable, royalty-free basis to BMJ Publishing Group Ltd ("BMJ") its licensees and where the relevant Journal is co-owned by BMJ to the co-owners of the Journal, to publish the Work in this journal and any other BMJ products and to exploit all rights, as set out in our licence.

The Submitting Author accepts and understands that any supply made under these terms is made by BMJ to the Submitting Author unless you are acting as an employee on behalf of your employer or a postgraduate student of an affiliated institution which is paying any applicable article publishing charge ("APC") for Open Access articles. Where the Submitting Author wishes to make the Work available on an Open Access basis (and intends to pay the relevant APC), the terms of reuse of such Open Access shall be governed by a Creative Commons licence – details of these licences and which Creative Commons licence will apply to this Work are set out in our licence referred to above.

Other than as permitted in any relevant BMJ Author's Self Archiving Policies, I confirm this Work has not been accepted for publication elsewhere, is not being considered for publication elsewhere and does not duplicate material already published. I confirm all authors consent to publication of this Work and authorise the granting of this licence.

For peer review only

BMJ OPEN

TITLE PAGE

TITLE: The impact of COVID-19 pandemic social restriction measures on people with rheumatic and musculoskeletal diseases in the UK: a mixed-methods study.

AUTHORS: Smith TO,[1,2] Belderson P,[1] Dainty JR,[1] Birt L,[1] Durrant K,[1] Chipping JR,[1] Tsigarides J,[1,3] Yates M,[1,3] Naughton F,[1] Werry S,[4] Notley C,[1] Shepstone L,[1] Macgregor AJ[1,3]

AFFILIATIONS

1. Faculty of Medicine and Health Sciences, University of East Anglia, Norwich, UK
2. Nuffield Department of Rheumatology, Orthopaedics and Musculoskeletal Sciences, University of Oxford, Botnar Research Centre, Windmill Road, Oxford, UK
3. Rheumatology Department, Norfolk and Norwich University Hospital, Norwich, UK
4. Patient and Public Representative, Norwich, UK

Corresponding Author: Professor Alex Macgregor, Faculty of Medicine and Health Sciences, Norwich Medical School, University of East Anglia, Norwich, NR4 7TJ, UK. Email: a.macgregor@uea.ac.uk

Word Count: 4527 manuscript

For peer review only

For peer review only - http://bmjopen.bmj.com/site/about/guidelines.xhtml

ABSTRACT

OBJECTIVES: To determine the impact of COVID-19 pandemic social restriction measures on people with rheumatic and musculoskeletal diseases (RMDs) and to explore how people adapted to these measures over time.

DESIGN: Mixed-methods investigation comprising a national online longitudinal survey and embedded qualitative study.

SETTING: UK online survey and interviews with community-dwelling individuals in the East of England.

PARTICIPANTS: People in the UK with RMDs, were invited to participate in an online survey. A subsection of respondents were invited to participate in the embedded qualitative study.

PRIMARY AND SECONDARY OUTCOME MEASURES: The online survey, completed fortnightly over 10 weeks from April 2020 to August 2020, investigated changes in symptoms, social isolation and loneliness, resilience and optimism. Qualitative interviews were undertaken assessing participant's perspectives on changes in symptoms, exercising, managing instrumental tasks such a shopping, medication and treatment regimens, and how they experienced changes in their social networks.

RESULTS: 703 people with RMDs completed the online survey. These people frequently reported a deterioration in symptoms as a result of COVID-19 pandemic social restrictions (52% reported increase vs. 6% reported a decrease). This was significantly worse for those aged 18 to 60 years compared to older participants (p=0.017). The qualitative findings from 30 individuals with RMDs suggest that the greatest change in daily life was experienced by those in employment. Although some retired people reported reduced opportunity for exercise outside their homes, they did not face the many competing demands experienced by employed people and people with children at home.

CONCLUSIONS: People with RMDs reported a deterioration in symptoms when COVID-19 pandemic social restriction measures were enforced. This was worse for working-aged people. Consideration of this at-risk group, specifically for the promotion of physical activity, changing home-working practices and awareness of healthcare provision is important, as social restrictions continue in the UK.

Keywords: COVID-19 pandemic; lockdown; rheumatology; musculoskeletal diseases; social isolation; occupational adaptation; qualitative; survey

For peer review only

STRENGTHS AND LIMITATIONS OF THIS STUDY

- This study reports the impact of COVID-19 social restrictions during the first COVID-19 'lockdown' on the lived-experiences of people with RMDs.
- This UK longitudinal survey of 703 people included an embedded qualitative study providing explanations on the quantitative results.
- A breadth of important health domains were examined over the initial COVID-19 10-week social restriction period, including pain, social isolation and loneliness, resilience and optimism.
- We only recruited UK residents so while the principles may apply to countries experiencing social restrictions due to COVID-19, results may not be generalisable beyond the UK.

For peer review only - http://bmjopen.bmj.com/site/about/guidelines.xhtml

INTRODUCTION

Rheumatic and musculoskeletal disease (RMDs) are a major cause of disability and reduced quality of life worldwide.[1] People with RMDs experience pain, joint stiffness, fatigue and muscle weakness with resultant physical disability. These symptoms are frequently managed with a combination of physical activity and medications, which for some people include immunosuppressive drugs.

In March 2020, the World Health Organization (WHO) declared the outbreak of a novel coronavirus disease SARs-CoV-2 (COVID-19) to be a pandemic. On 16th March 2020, the UK government instructed all people aged 70 years and over to self-isolate in their homes for up to four months. This was extended to all people on 23rd March for an initial three-week period. People who were taking immunosuppressant medications or with medical comorbidities were instructed to maintain strict self-isolation principles (often referred to as shielding) for an extended period of at least 12 weeks (**Supplementary File 1**).

At the outset, there was concern that people with RMDs may be particularly vulnerable to the unintended consequences of these 'lockdown' measures. They are recognised to be at increased risk of social isolation, through poor social support,[2] the frequent presence of other medical conditions[3,4] and functional disability.[5] Social isolation itself can affect levels of physical disability, psychological distress and pain in RMDs.[6] The loss of exercise opportunities, a key component of self-management programmes, risked increasing levels of disability and reducing mental wellbeing. COVID-19 pandemic measures also significantly impacted on routine healthcare access and NHS services.[7,8] Prior to this study, the resilience of this group to these enforced changes was unknown, as were likely long-term impacts.

This study explores the impact of COVID-19 pandemic social restriction measures on people with RMDs and determines how people adapted to these measures over time. Our aim was to provide insight into the challenges and potential impacts of those most at risk, and to make recommendations as to how best to support people with RMDs throughout further social restriction measures and beyond.

METHODS AND ANALYSIS

<u>Design</u>

Mixed-methods investigation comprising a national online longitudinal survey and embedded qualitative study. The study is reported in accordance with the STROBE statement for reporting observational studies[9] and the COREQ statement for reporting qualitative research.[10]

<u>**Survey Study**</u>

<u>*Cohort and recruitment*</u>

We invited participants who self-reported a diagnosis of chronic (three months or longer) RMDs (disease of the bone, joint or muscle), and who lived in the UK to participate in an online survey.

We used three approaches to recruit potential participants. Firstly, we approached patient-based organisations (Versus Arthritis, Arthritis Action, Royal Osteoporosis Society, National Rheumatoid Arthritis Society, National Ankylosing Spondylitis Society, Fibromyalgia UK, Age UK, PainConcern and

For peer review only - http://bmjopen.bmj.com/site/about/guidelines.xhtml

For peer review only

Scope). All agreed to disseminate an invitation to the survey via their online patient forum or as an email. Secondly, we contacted members from an inception cohort of 1,396 people with inflammatory arthritis – the Norfolk Arthritis Register (NOAR).[11] Finally, we used research team members' Twitter accounts to publicise the study.

Data collection

An online survey administered through the Qualtrics platform (Qualitrics XM, https://www.qualtrics.com/uk/) was provided to all participants. The survey is presented in **Supplementary File 1**. Participant consent was obtained through the platform before proceeding to the study survey. The survey collected information on:

- age, gender, ethnicity, duration of symptoms, type of RMD, medical co-morbidities.
- clinical disease activity.
- changes in medication use, access to healthcare support, physical activity, disease symptoms since COVID-19 lock-down.
- Clinical Health Assessment Questionnaire (CLINHAQ)[12] to assess disability and symptom status including: pain, fatigue, sleep, anxiety.
- the Lubben Social Network Scale 6[13] – short assessment of social isolation.
- University of California, Los Angeles (UCLA) three-item loneliness scale.[14]
- 6-item Brief Resilience Scale (BRS-6).[15]
- revised Life Orientation Test (LOT-R).[16]

We opened Round 1 (baseline) recruitment from 28th April 2020 to 27th May 2020. We sent tweets weekly during Round 1 recruitment period. For those who completed Round 1, we sent the same survey at two-week intervals to investigate how symptoms, social isolation, loneliness, resilience and optimism changed over time (Rounds 2 to 6). We sent a reminder email to participants who missed a subsequent round. The final survey was completed on 20th August 2020.

Data analysis

The analysis first addressed the perceived changes in symptoms since the start of lockdown in response to the question, *"How have your symptoms been since the current COVID measures started?".* The responses were scored as decreased, stable, or increased. The influence of possible explanatory variables on the categories of symptom response was assessed through chi-squared tests. Data were analysed using R (R Core Team[17]).

A second analysis examined whether there were any prospective changes over the 10-week observation period in variables (pain, fatigue, anxiety, sleep, social networks, loneliness, resilience, optimism) that could be sensitive to change as the UK Government guidelines on social restriction measures altered (**Supplementary File 2**). There was a focus on differences in the two age groups (18-60 years and 60+ years) with the cut-off chosen to approximate to 'working' (n=351) and 'non-working' groups (n=351). Mixed models were used to analyse repeated data from the same participants. Time was included as a fixed-effect and 'participant ID' as a random-effect. All analyses were conducted in R using the 'clmm' function in the 'ordinal' package[18] for ordinal response variables or the 'lmer' function in the 'lme4' package[19] for continuous response variables.

Qualitative Study

The qualitative study was an embedded interview study which aimed to explore the subjective experiences of people with RMDs during COVID-19 pandemic social restrictions.

Sample and recruitment

For peer review only - http://bmjopen.bmj.com/site/about/guidelines.xhtml

For peer review only

Survey participants recruited through NOAR were offered the option to take part in interviews; 137 expressed an interest. We designed a purposive sampling strategy, recruiting to achieve variation in age and gender, recognising that rheumatoid arthritis (RA) is more common in women and people aged over 40 years. A sample size of 25 to 30 was planned to support data collection in the window of guidance on self-isolation. This sample target was sufficient to enable robust themes to be developed. All those contacted agreed to take part.

Data collection

The interview topic guide (**Supplementary File 3**) took a narrative approach, starting with open questions on everyday life and then focused on the participant's perspective on changes in symptoms, exercising, managing instrumental tasks such a shopping, medication and treatment regimens, and how they experienced changes in their social networks. Questions were reviewed by clinical and lay colleagues for clarity and relevance. Participants were approached by two researchers (LB, PB) over the telephone or email and received study information 72 hours prior to interview. All gave recorded verbal or written consent before the interview commenced. All interviews were conducted by two experienced qualitative researchers (LB, PB) over the telephone due to COVID-19 restrictions and were audio recorded for transcription. Interviews lasted between 30 and 90 minutes; average duration was 45 minutes.

Data analysis

Data were analysed using a thematic analysis approach guided by the six-steps outlined by Braun and Clarke.[20] As a first step, interviews were transcribed and read to aid familiarization with the data. Two researchers (LB, PB) coded the data in NVivo 12. During coding, descriptive categories were identified relating to symptom attribution, changes to everyday life and wellbeing, narrative of vulnerability, and risk management. Frequent researcher and multidisciplinary meetings led to interpretive themes.[21] To enhance the dependability and confirmability of the results, peer and participants validation was completed.[22] Emerging themes and categories were shared across the research team and rheumatology health practitioners. Interview participants were sent a summary of key themes over email, followed by a phone call to each seeking feedback during August-September 2020. There was strong resonance between the themes and participants' experiences.

The qualitative data were reviewed alongside the survey data to provide detailed explanations of experience of symptoms and wellbeing since restrictive measures commenced. Through this, an interpretative synthesis of results from each research methodology was presented using a mixed-methods approach.

PATIENT AND PUBLIC INVOLVEMENT

Patient involvement began during protocol development stage of the study protocol and continued throughout. A patient-member (SW) provided her personal insights on the questions posed in the survey and qualitative topic guide. She provided advice into the interpretation of the survey and interview analysis. Our patient member assisted in the preparation of the final paper. She will continue to support dissemination through the preparation of public documents and social media outputs to share the results to wider patient communities.

RESULTS

For peer review only - http://bmjopen.bmj.com/site/about/guidelines.xhtml

Survey Results

Baseline assessment

In total, 703 respondents (574 females, 126 males, three others or non-binary) were recruited (262 from NOAR and 441 from the wider-UK base). Their characteristics are summarised in **Table 1**. The majority of respondents were aged 51 to 70 (508/703; 72.3%) and residing in England (660/703; 93.9%). A range of inflammatory and non-inflammatory diseases were represented with RA (44.2%) and osteoarthritis (21.6%) reported most frequently. There were no important differences in the characteristics or responses in the NOAR and non-NOAR identified RA patients, and their data were pooled for analysis.

Table 2 illustrates respondent's symptoms at baseline compared with before the imposition of the COVID-19 pandemic social restriction measures. In total, 365 (52%; 95% CI: 49% to 56%) reported an increase in symptoms related to their RMD, 287 (41%; 95% CI: 37% to 45%) reported that they had stayed the same and 45 (6%; 95% CI: 5% to 9%) reported decreased reduction in symptoms.

Age had a significant influence on the change in symptoms, with a higher proportion of younger respondents (age ranged 18 to 60 years) reporting an increase in symptoms (p=0.017). As might be expected, people who experienced an increase in symptoms were more likely to have changed their medication (p<0.001) and have needed to access medical advice (p<0.001). Physical activity also varied according to the change in reported symptoms (p<0.001): those who reported that their symptoms had increased also reported a decreased level of physical activity (**Table 2**).

Follow-up assessment

Approximately 25-30% of the participants were lost to attrition over the course of the study. At the second timepoint (week=2), there were 525 participants, at week=4 there were 506, at week=6 there were 540, at week=8 there were 521 and at week=10 there were 491 participants. **Supplementary File 4** compares the characteristics of participants at baseline and week=10 and indicates that they were similar. **Figure 1** and **Figure 2** illustrate the changes in responses over 10 weeks stratified by age (the strongest predictor of the increased level of symptoms). For all variables examined (symptoms, supportive social networks, loneliness, resilience and optimism), the younger age groups (18 to 60 years) fared worse than the older group (60 years plus) across all time points. Levels of optimism amongst the younger age groups fell into the range that is classified as 'low'. For the 18 to 60 years age group, there was a significant improvement in the levels of pain (p<0.001) and sleep (p<0.001), over the 10-week period, whilst anxiety levels (p=0.769) and fatigue (p=0.920) stayed the same. The 60 years plus age group had significant improvements in pain (p<0.001) and fatigue (p=0.002) but sleep (p=0.080) and anxiety (p=0.610) stayed the same over the 10 weeks (**Figure 1**). The size of any improvements was marginal in both age groups. In contrast, feelings of social isolation intensified (18 to 60 years: p<0.001; 60 years plus: p<0.001), and levels of resilience (18 to 60 years: p<0.001; 60 years plus: p<0.001) and optimism were significantly reduced (18 to 60 years: p=0.008; 60 years plus: p=0.009; **Figure 2**) but, the effect sizes were, again, small and unlikely to be of clinically relevant.

Qualitative Study

Interviews were undertaken with 26 participants between 27th May 2020 to 19th June 2020. The sample consisted of nine men and 17 women, with a mean age of 59 years (range: 29 to 83 years). Eighteen participants were diagnosed with RA, five with psoriatic arthritis, two with polymyalgia

For peer review only - http://bmjopen.bmj.com/site/about/guidelines.xhtml

rheumatica and one with inflammatory polyarthritis. At the time of the baseline survey, 14 had reported increased symptoms, nine stable symptoms and three decreased symptoms (**Table 3)**.

Most participants described themselves as shielding, although there was variation in interpretation and adherence to the formal guidelines. There were narratives of resilience, acceptance and adaptive coping strategies:

> *I put up a little poster just to remind myself that I can only change what I am able to change. I just need to try and accept what I can't change.* [RMD1 Female 50-54]

Nonetheless, most highlighted challenges or stresses associated with the COVID-19 pandemic. In some, this was accompanied by reported worsening symptoms, including increased pain, stiffness and fatigue. Not all participants directly attributed their experience of symptoms to the COVID-19 context: *"My arthritis has flared up. I don't know if it's the change in situation or it was going to happen anyway."* However, many spoke about reasons related to the pandemic which they perceived had underpinned symptom changes. Several speculated that a combination of these factors may have been at play. The reasons are outlined thematically below and summarised in **Table 4.**

Accessing health care

Many participants described 'holding on' to concerns about their disease because they did not want to trouble health services for fear of burdening the service, stating staff might be too busy due to COVID-19. Some had decided to miss blood tests due to anxiety around exposure to infection. Others were self-managing their symptoms:

> *If I get any breakthrough pain where I'm really not happy, I can take two of my tramadol and…I take nortriptyline at night and I make sure that I'm getting a decent night's sleep. I've just been doing a lot of self-care.* [RMD26 female 55-59]

Health concerns were also sometimes not reported due to postponed or cancelled appointments. There was a commonly expressed acceptance of this situation, feeling that others are 'worse off' at a time of crisis: *They cancelled my appointments…under the circumstances, you accept it…and think well, there's people who are suffering and I'll just stand by.* [RMD6 male 70-74]

However, missing or postponed appointments created anxiety and a sense of detachment from healthcare support for some, compounding delayed advice seeking: *"I would have brought it up [at postponed appointment] and it would have been good to discuss those things, and you do worry"* [RMD13 female 30-34]. Some had telephone appointments instead and although many preferred this, feeling it to be safer during the pandemic, some said that telephone appointments did not substitute entirely for face to face contact: *"They can't see the pain you're in; they can't feel your joints, they can't do any of that…does make everything so much trickier"* [RMD23 female 45-49]. Others explained that they avoided raising what they thought of as smaller concerns during a telephone call. One person described a physiotherapy telephone appointment which they acknowledged had been helpful, but also said *"[I] need human intervention for my body"* [RMD10 female age 55-59].

Physical activity

Shielding instructions, lockdown and fear of catching COVID-19 meant many interviewed participants reported being much more sedentary and not leaving the house at all. The most common attribution for symptom change, including pain, stiffness and fatigue, was reduced activity: *"I think that's just lack of movement, the lack of being able to move about. I think, over time, your body just shuts down due to lack of mobility"* [RMD10 female age 55-59].

Some discussed reduced motivation or limited opportunity to be able to engage in exercise: " *I used to go to the gym a lot, just to force myself. But they're all closed"* [RMD22 male 45-49]. Reduced

For peer review only - http://bmjopen.bmj.com/site/about/guidelines.xhtml

activity was also linked to disruption of everyday routine. This was more often discussed by those of working age who described ceasing a regular daily walk or cycle commute to work, or being on their feet, not only in manual jobs but also within the context of office-based work:

> *Obviously, with me not working, then that's a huge change, I actually spend my life going from one of the departments to another… I would say my exercise routine is pretty rubbish, I'll be honest I have put some weight on during lockdown. That could be part of [worse symptoms], let's face it."* [RMD1 female 50-54]

None reported having received specific advice about maintaining exercise for joint health at the time of interview, but a number were trying to substitute with exercise at home, (e.g. using a treadmill, online classes or walking round the garden). Exercising while avoiding contact with others was even more challenging without access to a private garden space:

> *I've got a long balcony here, it's about 100 yards long. So I should walk round there, but again, you've got to restrict that, because you never know if you're going to meet someone the other way. There isn't two metres to pass….you find you're just getting stiffer and stiffer.* [RMD6 male 70-74]

Home work-space and tasks at home

Several interviewees explained they were working from home in environments which were far from ideal for the maintenance of good joint health, for example using a dining room chair and laptop rather than a desktop computer and office chair. Many envisaged home working for the foreseeable future. Levels of support and provision of equipment from employers was variable. Some employers had made swift efforts to prioritise provision of suitable equipment. However others had not received this support, and one participant was hesitant to complain for fear of losing their job at a time of job insecurity:

> *I'm just sitting at the kitchen table with my laptop. [My employer] said they couldn't do anything because there were so many of us that weren't at work. I was just like well, I'll just get on with it then, because I kind of need my job*. [RMD23 female 45-49]

In response to exacerbation of pain, some interviewees had purchased more ergonomic equipment themselves, while others felt they did not have the space to accommodate this kind of office set-up within their home.

A few participants who were shielding discussed no longer having access to assistance with household tasks which had adversely impacted on their physical or mental health. A few spoke of pacing out household activities but had not received professional advice on this.

Wellbeing

Descriptions of low mood, isolation and boredom were common in the interviews. Many explained they were anxious about being in a clinically-vulnerable group, and also reported a sense of being left behind or forgotten about. Many expressed worries about the future and some felt they would continue staying at home, regardless of relaxation of guidelines. A few said only a vaccine would make them feel 'safe'. Some made an explicit direct link with pain and fatigue symptoms, speculating for example that increased pain may be linked to stress surrounding their current situation. Others commented on the effect on their fatigue levels: *"I've felt more tired, but I think that is because I felt more anxious and I haven't slept so well…..I'd say it's more linked to worrying about COVID"* [RMD11 female 50-54].

For some with caring responsibilities, increased demands and stress associated with managing multiple roles at home, working while schools were closed, was identified as having contributed to

For peer review only - http://bmjopen.bmj.com/site/about/guidelines.xhtml

symptom flare-up. There was also additional stress around decision-making and risk assessment in the context of family life, managing dilemmas about protecting their own health against wider family wellbeing.

> *It's been exhausting, absolutely exhausting. I was getting [child] to try and do his work, but he really needs one-to-one because he gets bored so quickly. So that became a bit of a nightmare… and then working in the evening to kind of catch up….no way of, if my arthritis is really bad, of just resting.* [RMD23 female 45-49]

Improving or stable symptoms

In those who perceived no change in their condition, some attributed this to it having been in a stable, long-term state, for instance with disease already well-controlled by medication or conversely was "*already bad*". One participant who reported stable symptoms credited, in part, the support he had received from his employer prioritising him to receive appropriate office equipment for working at home. Others attributed their stable condition to maintaining routine, exercise and other protective coping behaviours: *[My symptoms] seem to be about the same really. I know if I sit about I'll get stiff, so I do an exercise programme every day and try to keep myself moving around."* [RMD16 female 75-79]

A small number of those interviewed explained that their symptoms had markedly improved as a consequence of COVID-19 measures. One cited better opportunity and motivation to take more exercise such as walks, or joining children for daily exercises. Another participant described benefitting from being at home and a slower pace of activity, and that shielding had offered a period of respite during which they were better able to cope with their condition and prioritise self-care:

> *I'm furloughed….Having the time to just be at home, no one's expectations, my body has a chance to heal and find some balance… it makes you wonder. There's obviously no cure for RA at the moment but it does make you, you know, that emotional health is so key.* [RMD21 female 45-49]

Supplementary File 5 provides a summary of an interpretive synthesis of results, with interview findings, offering an explanatory insight into potential reasons for changes identified through the quantitative investigation.

DISCUSSION

Within the survey cohort, we found that people with RMDs frequently reported a deterioration in pain and symptoms, in addition to greater social isolation, loneliness and reduced optimism to their circumstances. This was significantly worse for those aged 18 to 60 years in comparison to older participants. The qualitative findings suggest that more types of change to daily life was experienced by those in employment. Although some retired people reported reduced opportunity for exercise outside the home, they did not face the many competing demands experienced by employed people and people with children at home.

Relationship to previous evidence

Recently, Persiani et al[23] and Garrido-Cumbrera et al[24] reported their findings of the impact of the first COVID-19 pandemic 'lockdown' in with people with RMDs. These findings reflect those of our UK cohort which illustrated a reduction in physical activity and increase in pain, particularly amongst people aged 50 to 70 years and in work.[23] and poor lifestyle habits negatively impacting on overall

For peer review only - http://bmjopen.bmj.com/site/about/guidelines.xhtml

physical and mental health.[24] These findings may be attributed to a disruption in their normal working patterns, changes in their home environment and loss of social networks. Our qualitative interviews indicated that the enforced COVID-19 pandemic social restrictions led to stressful changes in working routine, the addition of extra roles within the home (e.g. through home schooling) and deleterious workstation ergonomics due to lack of space and equipment. The latter point may be particularly important. Where occupational health requires work-station assessments for ergonomic safety, these were not practical in this phase of the pandemic. As people with pre-existing RMDs are particularly sensitive to the negative impacts of poor static postures, changes in workspace and working hours may have accounted for these reported physical responses.[25] As homeworking continues, employers should be aware of this challenge for employees with existing RMDs, to help reduce home issues that could lead to symptom flares.

Clinical implications

The findings have implications for clinical practice. Firstly, the cohort we recruited largely demonstrated persistence in their worsening symptoms over time. As the qualitative research highlighted, reluctance to engage with health services, particularly during times of higher perceived risk, may mean that individuals are less likely to seek help for physical and mental health or only seek help when their symptoms become more severe, which may be more challenging to manage. Advertising and promoting services and how to access these may therefore be important strategies across primary and secondary care services to negate this issue. Secondly, due to changes in daily routine and increased symptoms, there may be greater disability within this population. Support to encourage resumption of physical activity routines and encouraging activity and engagement may be important for all.[26,27] Finally, these findings indicate that younger, working aged-people with RMDs were particularly affected by COVID-19 pandemic restrictions. Specific consideration of the impact on these individuals should be considered as they, potentially unexpectedly, may be at considerable risk of poorer musculoskeletal outcomes compared to older individuals. Employers should ensure that their workers, whilst working from home, have suitable risk assessments and provision of equipment to minimise the risks of exacerbating musculoskeletal pain, particularly for those with pre-existing RMDs.

In the months after the data for this paper were collected (August 2020), some enforced changes have been lifted. However, for many people in the UK and internationally, measures to restrict social activity remain and their impacts are ongoing. The findings from this study therefore remain pertinent.[28] Furthermore, whilst some activities can be recommenced, such as attending hospital appointments and communal exercise, many people reported longer-term concerns regarding their risk of COVID-19 and have refrained from resuming their normal routines. Additional support may be required to encourage individuals with the greatest anxiety to return to some of these social pursuits. This will be particularly important to avoid problems associated with social isolation, once longer-term restrictions are lifted. Previous literature has demonstrated the association between social isolation and higher incidence of cardiovascular disease,[29,30] mental conditions,[31] dementia[32] and mortality.[33]

Strengths and limitations

This study is a large, national cohort of individuals with RMDs. The mixed-methods approach provides novel and important real-time exploratory and explanatory findings to the changes in symptoms that this cohort reported during the initial 10 weeks of COVID-19 pandemic restrictions. Method triangulation[34] led to a more comprehensive understanding of the experiences of this specific group of people who may be considered particularly at risk of unintended consequences from social restrictions during the pandemic.

For peer review only - http://bmjopen.bmj.com/site/about/guidelines.xhtml

Whilst these are key strengths, the study presents with three important limitations. Firstly, whilst we can report the self-reported levels of disability and impairment, the sample were self-selecting when completing the survey. Whilst the characteristics of this cohort are typical of those with RMDs,[35] responder bias may have an impact on the external validity of these findings. Secondly, whilst we are able to make assumptions based on a cohort that includes a range of different RMDs, we acknowledged that only a small proportion of the population were BAME and we did not collect data to assess the respondents' level of social deprivation. Previous literature has highlighted these two factors to be potentially important in determining COVID-19 prognosis.[36] Including these groups may have provided a different description of the behaviours and perceptions of these individuals towards COVID-19 and the impact on their symptoms. Finally, the qualitative study recruited people from the NOAR cohort who had also completed the survey. These were all patients with inflammatory rheumatological conditions. It remains unclear whether the responses those individuals included in our study can be applicable to non-inflammatory musculoskeletal conditions.

CONCLUSIONS

People with RMDs frequently experienced deterioration in pain and symptoms when COVID-19 pandemic social restriction measures were enforced. As the restrictions continued over the following 10-weeks, levels of social isolation and loneliness increased and optimism decreased. These changes were greater among those aged 18 to 60 years when compared with older age groups. Close attention to those at risk through the promotion of physical activity, changing home-working practices and awareness of healthcare provision is important as social restrictions continue in the UK.

For peer review only - http://bmjopen.bmj.com/site/about/guidelines.xhtml

DECLARATIONS & ACKNOWLEDGEMENTS

Authors' Contributors: TS, PB, JD, KD, JC, JT, MY, FN, SW, CN, LS, AM researched the topic and devised the study; KD, JC, TS, MY performed the quantitative data collection; JD performed the statistical analyses; PB, LB performed the qualitative data collection; PB, LB performed the qualitative analysis; SW provided Patient and Public Involvement support; TS, PB, JD, KD, JC, JT, MY, FN, SW, CN, LS, AM provided the first draft of the manuscript; TS, PB, JD, KD, JC, JT, MY, FN, SW, CN, LS, AM contributed equally to manuscript preparation; TS, PB, JD, KD, JC, JT, MY, FN, SW, CN, LS, AM approved the submitted manuscript. AM and TS obtained research funding. AM acts a guarantor.

Funding: Funding was obtained to support the conduct of this study from Action Arthritis (Norfolk) and the University of East Anglia.

Ethical Considerations: Ethical approval was obtained from the University of East Anglia's Faculty of Medicine and Health Science's Research Ethics Committee (RE: 2019/20-104 & 2019/20-105)

Conflict of Interest: No author declares a conflict of interest in relation to this work.

Trial Sponsor: University of East Anglia (Research and Innovation Team, Registry and Council House, University of East Anglia, Norwich, NR4 7TJ. Email: RIN.reception@uea.ac.uk). The views expressed are those of the author(s) and not necessarily those of the sponsor.

Provenance and Peer Review: Not commissioned; externally peer reviewed.

Acknowledgements: The authors thank the following organisations who assisted in distributing the survey across their networks: Pain Concern; Arthritis Action; National Rheumatoid Arthritis Society; National Ankylosing Spondylitis Society; Paget's Association; Parathyroid UK; The Health Policy Partnership; and Scope.

Data Sharing: Data are available upon reasonable request. Additional data available upon request.

Patient consent for publication: Not required.

Word count: 4527

For peer review only

TABLE AND FIGURE LEGENDS

Figure 1: Change in symptoms from baseline over the 10-week follow-up interval.

Figure 2: Change in behaviour outcomes from baseline over the 10-week follow-up interval.

Table 1: Respondent characteristics and responses to health provision access from online survey at baseline.

Table 2: Association at baseline between rheumatic and musculoskeletal diseases (RMD) symptoms and selected participant characteristics and questionnaire responses.

Table 3: Interview sub-sample characteristics (N=26).

Table 4: Key themes generated from the qualitative study - perceived underlying attributions for symptom change.

Supplementary File 1: Online survey (baseline).

Supplementary File 2: Timeline of study activity against UK legislation and COVID policy implementation.

Supplementary File 3: Interview topic guide.

Supplementary File 4: Characteristics of participants at baseline and Week 10.

Supplementary File 5: Interpretive synthesis of quantitative and qualitative findings.

For peer review only

For peer review only - http://bmjopen.bmj.com/site/about/guidelines.xhtml

REFERENCES

1. GBD 2015 Disease and Injury Incidence and Prevalence Collaborators. Global, regional, and national incidence, prevalence, and years lived with disability for 310 diseases and injuries, 1990–2015: a systematic analysis for the Global Burden of Disease Study 2015. Lancet. 2016;388(10053):1545–1602.

2. Ethgen O, Vanparijs P, Delhalle S, Rosant S, Bruyère O, Reginster J-Y. Social support and health-related quality of life in hip and knee osteoarthritis. Quality of Life Research. 2004;13(2):321-30.

3. Veronese N, Smith T, Reginster JY, Maggi S. Osteoarthritis increases the risk of cardiovascular disease: data from the osteoarthritis initiative. J Nutr Health Aging. 2018;22(3):371-376.

4. Veronese N, Stubbs B, Solmi M, Smith TO, Noale M, Cooper C, et al. Association between lower limb osteoarthritis and incidence of depressive symptoms: data from the osteoarthritis initiative. Age Ageing. 2017;46(3):470-476.

5. McAlindon T, Cooper C, Kirwan J, Dieppe P. Determinants of disability in osteoarthritis of the knee. Annals of the rheumatic diseases. 1993;52(4):258.

6. Smith TO, Dainty JR, Williamson E, Martin KR. Association between musculoskeletal pain with social isolation and loneliness: analysis of the English Longitudinal Study of Ageing. Br J Pain. 2019;13(2):82-90.

7. Pope JE. What does the COVID-19 pandemic mean for rheumatology patients? Curr Treatm Opt Rheumatol. 2020;1-4.

8. Nune A, Iyengar K, Ahmed A, Sapkota H. Challenges in delivering rheumatology care during COVID-19 pandemic. Clin Rheumatol. 2020;39:2817-2821.

9. von Elm E, Altman DG, Egger M, Pocock SJ, Gotzsche PC, Vandenbroucke JP. The Strengthening the Reporting of Observational Studies in Epidemiology (STROBE) Statement: guidelines for reporting observational studies. J Clin Epidemiol. 2008;61(4):344-349.

10. Tong A, Sainsbury P, Craig J. Consolidated criteria for reporting qualitative research (COREQ): a 32-item checklist for interviews and focus groups. Int J Qual Health Care. 2007;19(6):349-357.

11. Symmons DP, Barrett EM, Bankhead CR, Scott DG, Silman AJ. The incidence of rheumatoid arthritis in the United Kingdom: results from the Norfolk Arthritis Register. Br J Rheumatol 1994;33:735–9.

12. Wolfe F. A brief clinical health assessment instrument: CLINHAQ. Arthritis Rheum. 1989;32 (suppl): S9.

13. Lubben J. Assessing social networks among elderly populations. Family Community Health. 1988;11:42-52.

14. Hughes ME, Waite LJ, Hawkley LC, Cacioppo JT. A short scale for measuring loneliness in large surveys. Results from two population-based studies. Res Aging. 2004;26:655-672.

For peer review only - http://bmjopen.bmj.com/site/about/guidelines.xhtml

15. Smith BW, Tooley EM, Christopher PJ, Kay VS. Resilience as the ability to bounce back from stress: a neglected personal resource? J Positive Psychol. 2010;5:166-176.

16. Scheier MF, Carver C, Bridges MW. Distinguishing optimism from neuroticism (and trait anxiety, self-mastery, and self-esteem): A re-evaluation of the Life Orientation Test. J Personality Social Psychol. 1994;67:1063-1078.

17. R Core Team. R: A language and environment for statistical computing. R Foundation for Statistical Computing, Vienna, Austria. Available at: www.R-project.org/. Accessed on: 04 Jan 2021.

18. Christensen RHB. Ordinal - regression models for ordinal data. R package version 2019.12-10. Available at: CRAN.R-project.org/package=ordinal. Accessed: 04 Jan 2021.

19. Bates D, Maechler M, Bolker B, Walker S. Fitting linear mixed-effects models using lme4. J Statist Software. 2015;67(1):1-48.

20. Braun V, Clarke V. Using thematic analysis in psychology. Qualitative Res Psychol. 2006;3(2):77-101.

21. Morse JM. Confusing categorises and themes**.** Qualitative Health Res. 2008;18(6):727-728.

22. Birt L, Scott S, Cavers D, Campbell C, Walter F. Member checking: a tool to enhance trustworthiness or merely a nod to validation? Qualitat Health Res. 2016;26(13):1802-1811.

23. Persiani P, De Meo D, Giannini E, Calogero V, Speziale Varsamis T, Cavallo AU, Martini L, Cera G, Coluzzi F, Villani C. The aftermath of COVID-19 lockdown on daily life activities in orthopaedic patients. J Pain Res. 2021;14:575-583.

24. Garrido-Cumbrera M, Marzo-Ortega H, Christen L, Plazuelo-Ramos P, Webb D, Jacklin C, Irwin S, Grange L, Makri S, Frazão Mateus E, Mingolla S, Antonopoulou K, Sanz-Gómez S, Correa-Fernández J, Carmona L, Navarro-Compán V. Assessment of impact of the COVID-19 pandemic from the perspective of patients with rheumatic and musculoskeletal diseases in Europe: results from the REUMAVID study (phase 1). RMD Open. 2021;7:e001546.

25. Baldwin D, Johnstone B, Ge B, Hewett J, Smith M, Sharp G. Randomized prospective study of a work place ergonomic intervention for individuals with rheumatoid arthritis and osteoarthritis. Arthritis Care Res. 2012;64:1527-1535.

26. Castro da Rocha FA, Melo LDP, Berenbaum F. Tackling osteoarthritis during COVID-19 pandemic. Ann Rheumat Dis. 2020. In Press. Online*:* doi: 10.1136/annrheumdis-2020-218372

27. Pinto AJ, Dunstan DW, Owen N, Bonfa E, Gualano B. Combating physical inactivity during the COVID-19 pandemic. Nat Rev Rheumatol. 2020;16:347–348.

28. Rogers NT, Waterlow NR, Brindle H, Enria L, Eggo RM, Lees S, et al. Behavioral change towards reduced intensity physical activity is disproportionately prevalent among adults with serious health issues or self-perception of high risk during the UK COVID-19 lockdown. Front Public Health. 2020;8:575091.

29. Valtorta NK, Kanaan M, Gilbody S, Ronzi S, Hanratty B. Loneliness and social isolation as risk factors for coronary heart disease and stroke: systematic review and meta-analysis of longitudinal observational studies. Heart. 2016;102(13):1009-1016.

For peer review only - http://bmjopen.bmj.com/site/about/guidelines.xhtml

30. Holt-Lunstad J, Smith TB. Loneliness and social isolation as risk factors for CVD: implications for evidence-based patient care and scientific inquiry. Heart. 2016;102(13):987-989.

31. Alpass FM, Neville S. Loneliness, health and depression in older males. Aging Mental Health. 2003;7(3):212-216.

32. Rafnsson SB, Orrell M, d'Orsi E, Hogervorst E, Steptoe A. Loneliness, social integration, and incident dementia over 6 years: prospective findings from the English Longitudinal Study of Ageing. J Gerontol B Psychol Sci Soc Sci2020; 75(1):114-124.

33. Steptoe A, Shankar A, Demakakos P, Wardle J. Social isolation, loneliness, and all-cause mortality in older men and women. Proc Natl Acad Sci USA. 2013;110(15):5797-5801.

34. Denzin NK. The research act: A theoretical introduction to sociological methods. New Jersey: Transaction Publishers, 1970.

35. Danielsson L, Kvarstein G, Bergvik S. Mediators of pain and physical function in female and male patients with chronic pain. J Pain Res. 2020;13:1059–1071.

36. Raisi-Estabragh Z, McCracken C, Bethell MS, Cooper J, Cooper C, Caulfield MJ, Munroe PB, Harvey NC, Petersen SE Greater risk of severe COVID- 19 in Black, Asian and Minority Ethnic populations is not explained by cardiometabolic, socioeconomic or behavioural factors, or by 25(OH)-vitamin D status: study of 1326 cases from the UK Biobank. J Public Health. 2020;42(3):451-460.

For peer review only - http://bmjopen.bmj.com/site/about/guidelines.xhtml

Table 1: Respondent characteristics and responses to health provision access from online survey at baseline

**Characteristics**		**Frequency (%)**
N		703
Gender	Female	574 (82.0)
	Male	126 (18.0)
	Non-binary	1 (0.1)
	Prefer to self-describe	1 (0.1)
	Prefer not to say	1 (0.1)
Age	18-30	21 (3.0)
	31-40	44 (6.3)
	41-50	113 (16.1)
	51-60	173 (24.6)
	61-70	203 (28.9)
	71-80	132 (18.8)
	80+	16 (2.3)
Region of respondent	England	660 (93.9)
	Scotland	21 (3.0)
	Wales	19 (2.7)
	Northern Ireland	2 (0.3)
Ethnic Group	White	682 (97.8)
	Mixed/Multiple ethnic group	4 (0.6)
	Asian	7 (1.0)
	Black, African, Caribbean	3 (0.4)
	Arab	1 (0.1)
RMD Diagnosis	Rheumatoid Arthritis	311 (44.2)
	Osteoarthritis	152 (21.6)
	Mechanical low back pain	70 (9.9)
	Fibromyalgia	68 (9.6)
	Psoriatic Arthritis	64 (9.1)
	Inflammatory Polyarthritis	63 (8.9)
	Hypermobility	40 (5.6)
	Specific RMD diagnosis not reported	33 (4.7)
	Connective tissue disease (e.g. Lupus, Scleroderma, Myositis)	25 (3.5)
	Ankylosing Spondylitis	22 (3.1)
	Osteoporosis	18 (2.5)
	Polymyalgia Rheumatica	10 (1.4)
	Ligament/Tendon Injury/Bursitis	9 (1.3)
	Neck pain	6 (0.8)
	Gout	5 (0.7)
	Other	63 (8.9)
How have your RMD symptoms been since the COVID measures started?	Decreased	45 (6.5)
	Stayed the same	287 (41.1)
	Increased	365 (52.4)
Pain (Scale: 0-10)	Mean (SD)	4.8 (2.6)
General Health (Scale: 0-10)	Mean (SD)	4.1 (2.4)
Total Lubben Social Network Score (Scale: 0-30)	Mean (SD)	14.8 (5.5)
Total UCLA Loneliness (Scale: 3-9)	Mean (SD)	5.1 (2.0)
Difficulty accessing medication	Yes	82 (11.7)
	No	616 (88.3)
Required someone to help access	Yes	309 (44.3)

For peer review only - http://bmjopen.bmj.com/site/about/guidelines.xhtml

medications	No	389 (55.7)
Changed medications since COVID-19 outbreak	Yes	103 (14.8)
	No	595 (85.2)
Required to seek advice from a health professional on condition	Yes	252 (36.1)
	No	446 (63.9)
Who did you contact	General Practitioner	158 (22.3)
	Practice Nurse/GP Nurse Practitioner	23 (3.2)
	Rheumatology Department	97 (13.7)
	Physiotherapy or Occupational Therapist	16 (2.3)
	Pharmacist	16 (2.3)
	Hospital Department (non-RMD)	10 (1.4)
	A&E	3 (0.4)
	Private chiropractor, osteopath or massage therapist	1 (0.1)
	Royal Osteoporosis Society	1 (0.1)
	Endocrinology Department	7 (1.0)
	Pain Clinic	2 (0.3)
	Counsellor or Health Psychologist	2 (0.3)
	Massage Therapist	1 (0.1)
	Podiatrist	1 (0.1)
	Nutritionist	1 (0.1)
	NHS 111	2 (0.3)
How easy has it been to get advice? (Scale: 0-10)	(Mean (SD) value. scale 0-10)	4.8 (3.3)

A&E- accident and emergency; CI – confidence intervals; JIA – Juvenile Inflammatory Arthritis; NHS – National Health Service; RMD - rheumatic and musculoskeletal diseases; SD – standard deviation; UCLA – University of California Los Angeles Loneliness Score

For peer review only

Table 2: Association at baseline between rheumatic and musculoskeletal disease (RMD) symptoms and selected participant characteristics and questionnaire responses.

	"How have your symptoms been since the current COVID measures started?"			**Chi-squared test of association with outcome (symptoms)**
	**Decreased**	**Stable**	**Increased**	p-value
n (%)	45 (6)	287 (41)	365 (52)	
Age group				p=0.017
18-60	22 (6)	125 (36)	200 (58)	
60 plus	33 (7)	162 (46)	165 (47)	
Gender				p=0.11
Male	8 (7)	60 (49)	54 (44)	
Female	37 (6)	224 (39)	311 (54)	
RMD diagnosis*				p<0.001
RA	17 (5)	155 (48)	149 (46)	
IA	4 (10)	24 (60)	12 (30)	
PsA	4 (6)	22 (34)	38 (59)	
Other	19 (7)	83 (31)	166 (62)	
Situation				p=0.880
At home	24 (7)	141 (40)	191 (54)	
Self-isolating	8 (7)	49 (40)	64 (53)	
Shielding	13 (6)	97 (44)	110 (50)	
Difficulty accessing medication?				p=0.103
Yes	4 (5)	26 (32)	52 (63)	
No	41 (7)	261 (42)	313 (51)	
Change medication?				p<0.001
Yes	9 (9)	21 (20)	73 (71)	
No	36 (6)	266 (45)	292 (49)	
Consult health professional?				p<0.001
Yes	15 (6)	64 (25)	172 (69)	
No	30 (7)	223 (50)	193 (43)	
Physical activity				p<0.001
Decreased	31 (7)	141 (30)	302 (64)	
Same	3 (3)	89 (75)	26 (22)	
Increased	11 (10)	57 (54)	37 (35)	

Data are frequency (%) unless stated otherwise; RMD = Rheumatic and musculoskeletal diseases

* RA=Rheumatoid arthritis, IA=Inflammatory arthritis, PsA=Psoriatic arthritis, Other=mainly Osteoarthritis (55%) but also including any diagnosis that was not RA, IA or PsA (see **Table 1**)

For peer review only - http://bmjopen.bmj.com/site/about/guidelines.xhtml

Table 3: Interview sub-sample characteristics (N=26)

Characteristics		Frequency
**Gender**	Female	17
	Male	9
**Age**	18-30	1
	31-40	4
	41-50	5
	51-60	4
	61-70	3
	71-80	8
	80+	1
**Ethnic Group**	White	26
**RMD Diagnosis**	Rheumatoid Arthritis	18
	Psoriatic Arthritis	5
	Inflammatory Polyarthritis	1
	Polymyalgia Rheumatica	2
**How have your RMD symptoms been since the COVID measures started? (reported at baseline)**	Decreased	3
	Stayed the same	9
	Increased	14

RMD = rheumatic and musculoskeletal diseases

For peer review only

1 **Table 4**: Key themes generated from the qualitative study - perceived underlying attributions for symptom change

Experience of symptoms	Attributions from interview data	Representative quote
**Increased symptoms**	**Accessing healthcare**<br>Holding on to concerns	*I don't like to worry them, they've got enough to do … It's something that I feel I can manage until when this is all over hopefully I'll get an appointment to go in and I'll mention it then.* [RMD17 female age 70-74]
	Postponement or cancelation of appointments, treatments and investigations	*I think that's something I feel like I've missed out on with the lockdown is, I would actually quite have liked to have some proper physio, especially on the knees and that. Because the swelling just doesn't seem to want to go, as much as I poke and prod it and things like that.* [RMD 10 female age 50-59]<br><br>*It was planned for me to have an MRI scan on my neck, but then this all happened and it's just sort gone out the window…. It's quite hard, day-to-day, with the pain levels in my neck, but under the current situation, I would rather put up with the pain than put myself at risk by going into a hospital.* [RMD20 male age 50-55]
	Telephone appointments	*Where I've missed out because of the lockdown, is that although when I phoned the rheumatology department they've been really helpful, it's not like having proper management. So all of the questions that I would normally have asked, I don't seem to ask so much on the phone... It would be nice to say to somebody, "This bit hurts, what can I do about it? Is this any good?" and things like that. But you need to have the physical and visual side of that.* [RMD 10 female age 50-54]
	**Reduced physical activity**<br>Reduced planned exercise (including less opportunity or access to facilities to exercise)	*I think I'm so much less active, before all this I was going swimming three times a week, that was the best exercise for me really, because it didn't put as much pressure on my body as going to the gym, running or anything like that. And, I don't think I fully appreciated how much swimming helped, whereas now, obviously, I'm not doing anything and I am just only getting out of the house for a little bit once a week, I've just found everywhere has just stiffened up so much…It's a bit scary, really.* [RMD5 female 25-29]<br><br>*[My joints are] getting stiff because you don't go out much to get exercise…. sitting down a lot makes your hips an your back ache and don't get up often where your chair is*. [RMD6 male 70-74]
	Reduced routine activity (e.g. walking to shops, going to work)	*I've been finding, like, sitting at my desk all day for work, I do get quite sore and quite stiff after work, because I think maybe when I was in an office environment I would be going out at lunchtime or just walking around the office to talk to different people…generally if I'm not sat at my desk working, I suppose I'm sat on the sofa, I'm not really that mobile around the house.* [RMD5 female 25-29]<br><br>*[Pain and stiffness] have been a bit more apparent because I haven't been able to get out as much and use my joints. I find when I've been cooped up in a space and sitting down for a while, then obviously it affects you more. I can walk to work and back again and it frees things up a little bit. But obviously not being able to do that, it's not the same… it gets worse when you can't be as mobile as you could be.* [RMD12 male 35-39]
	**Working at home**<br>Lack of ergonomic work-space	*At work I have a Varidesk…so, I can stand up. I also have like a proper chair, like a medical chair… [Working at home] was horrendous because I was working off my lap on a laptop, bending over. So, my back and my shoulder was very, very painful.* [RMD2 female 35-39]

For peer review only - http://bmjopen.bmj.com/site/about/guidelines.xhtml

	Struggling with household tasks	*I used to have someone come on a Thursday and do my chores for me…So perhaps it's something like that as well, you know, sort of without the help there…. I think with the loss of energy you get more of a hurting in the legs at night and that.* [RMD19 female 70-74]
	**Wellbeing** Managing multiple roles within the home (Home-schooling, childcare and work)	*I probably went way too hard on myself, trying to plan lessons for [children]. You're also then working, you're also trying to run the house… completely overdid it……I do think the flare that I had was related to being in lockdown. I'm not sure I would have had it that bad if we were out of lockdown, because I would have been able to maybe adjust working life, the girls would have been at school, or my parents would have had them…. It was definitely probably reactive from overdoing it… trying to juggle so many plates.* [RMD13 female 30-34]
	Anxiety or low mood associated with the pandemic and being in a clinically vulnerable group.	*I do struggle quite a lot with my mental health and I've found that my physical health has got so much worse, as well… it's definitely harder at the moment.* [RMD5 female 25-29]<br><br>*Currently, I've got swelling ….and I've got pain in multiple areas. So, I would say I'm having a flare up…maybe it's the stress had an impact on my immune system.* [RMD2 female 35-39]
**Stable symptoms**	Maintaining exercise	*In terms of my arthritis and stuff, that's not been – touch wood – too bad. I've tried to keep on top of it by trying to stay as active as I can really… I've been probably doing 2K, 3K, 5Ks a week and then us, as a family unit, we try and go for a walk…so the pain has not been too bad.* [RMD14-male-35-39]
	Pre-existing stable or well-controlled condition	*I don't think there's a lot of difference really, it's quite well controlled anyway.* [RMD4 female 65-69]
**Decreased symptoms**	An opportunity for respite, improved wellbeing	*The arthritis was getting worse [and since lockdown] I've had no flare ups. It's all been very much under control, and I think that's partly being removed from the work situation but it's partly having that easier pace of life, and if my body needs to sleep there's not an alarm set, it just sleeps. I think before this I was feeling very tired and unwell….I now feel far calmer, physically rested, which helps no end.* [RMD21 female 45-49]
	More opportunity for exercise	*I'm actually feeling really good right now….I like to think it's the exercises…I've definitely done more daily exercises with my son and skipping and stuff and more bike rides. I like cycling anyway, but I've been on more long bike rides and probably been taking the dog for a walk probably more than I'd done before.* [RMD11 female 50-54]

For peer review only - http://bmjopen.bmj.com/site/about/guidelines.xhtml

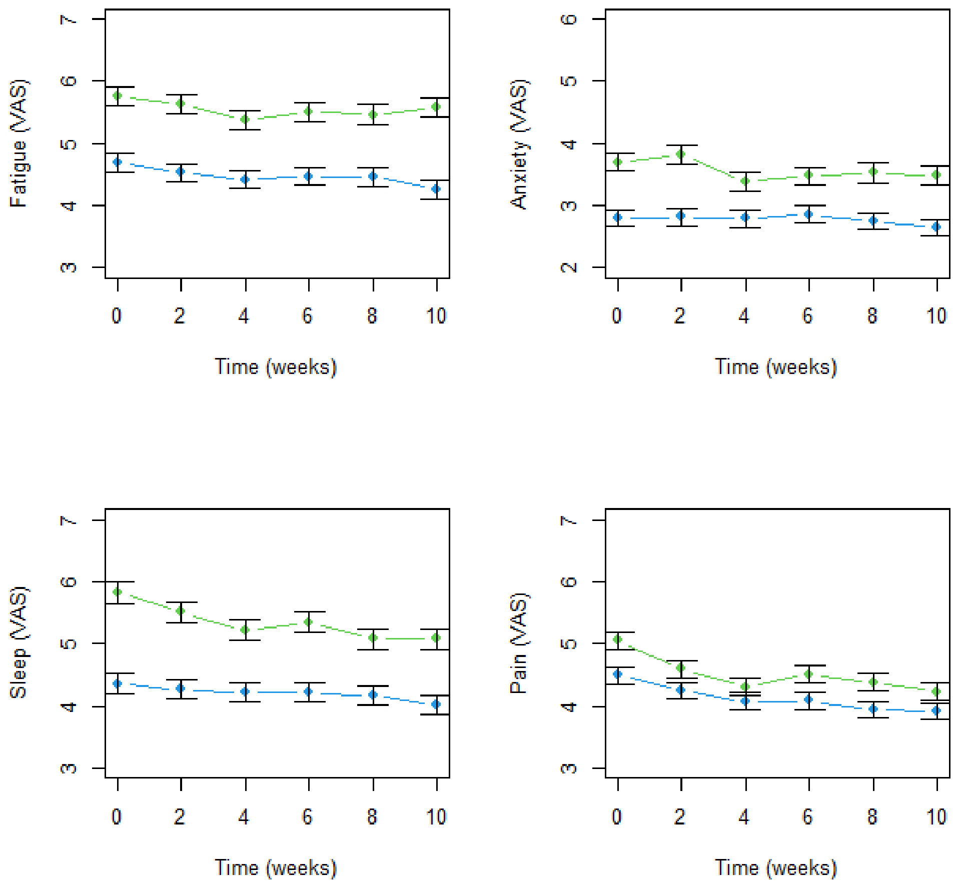

Figure 1: Change in symptoms from baseline over the 10-week follow-up interval.

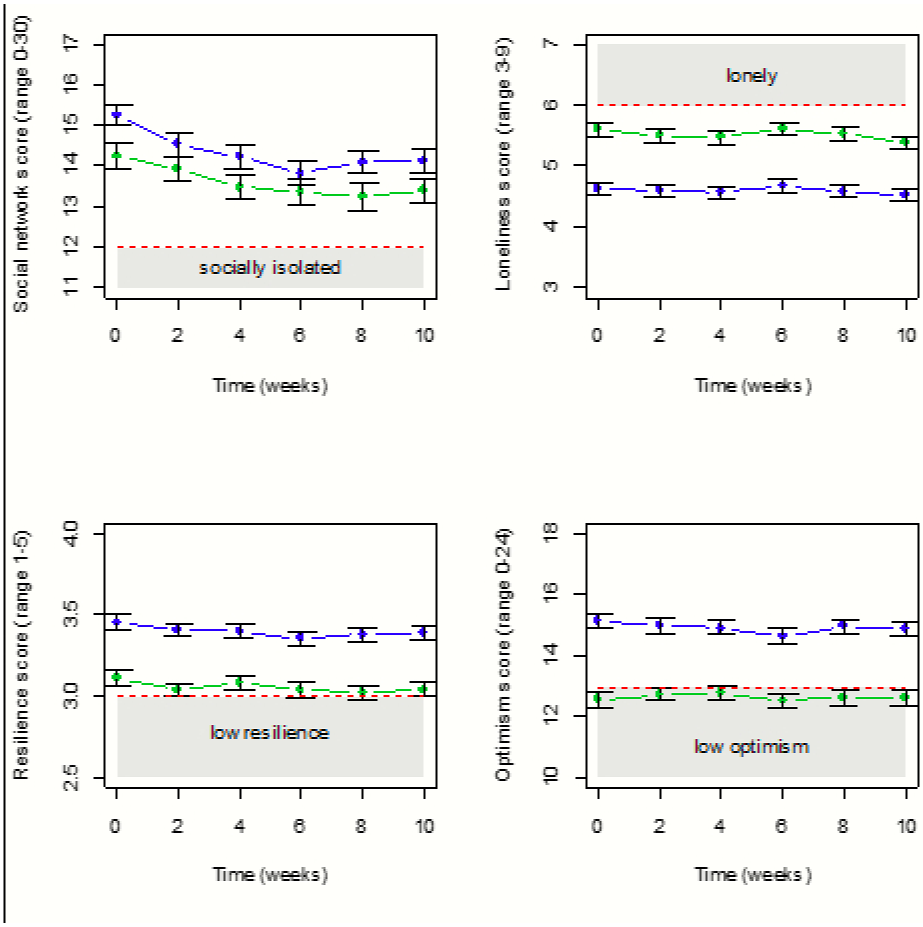

Figure 2: Change in behaviour outcomes from baseline over the 10-week follow-up interval.

Supplementary File 1: Online survey (baseline)

The impact of COVID-19 self-isolation measures on people with musculoskeletal diseases

Questionnaire

Version 0.1 - dated: 19 April 2020

Information to help you decide if you want to take part in this research

You are being invited to take part in a research project so we can understand how you are managing self-isolation during the coronavirus pandemic (Covid-19). We are particular interested in your joint pain, wellbeing and social contacts. Please ensure you have read and understood this information before continuing.

What is this project about?

This study aims to identify how the Covid-19 pandemic is impacting on the health and wellbeing of people with bone, joint or muscle pain (musculoskeletal disease). It aims to better understand how the current Covid-19 self-isolation is affecting people, their experiences of isolating and how they are managing their joint pain during this time. It will tell us about the effects of social isolation measures and changes in health services on people's physical health and wellbeing. The findings will be used to help understand who is at greatest risk of poor health and wellbeing during self-isolation so we can better support them during this outbreak.

Why have I been asked?

You have been sent this survey or have accessed it via social media. The project is entirely voluntary. You do not have to be in isolation to take part.

What will taking part involve?

You will be asked to complete a 15-minute survey online. Then every 2 weeks for the next 12 weeks, we will ask you to complete the same survey again. The whole study will last for about 12 weeks while these social isolation measures are in place.

What will you ask and what will happen to the information I give you?

You will be asked questions about yourself, your current joint pain, your medication and health care requirements for joint pain, and some questions on your physical health and wellbeing. We will also ask you whether you are isolating, some questions on social isolation, loneliness and resilience to this change in lifestyle, and some questions on your arthritis symptoms. Some of these questions are considered sensitive data, such as questions on your wellbeing. No information provided will be passed to any third parties. Your data will then be analysed by researchers from UEA and will be published in scientific papers and used to inform advice given during Covid-19. Nobody will be able to identify you from any materials we publish or present from this study.

You will only be identified to the research team members by your email address. We need this so that we can send you the follow-up fortnightly survey links for the next 12 weeks. We will

not use your email address for any other purpose. We will not share this email address with any other people or organisations.

This study has received approval from the University of East Anglia's Faculty of Medicine and Health Science's Research Ethics Committee.

How long will my data be stored for?

Your data will only be used for this study and not shared. Your data will be stored for 10 years by UEA after the end of the research. At this point the data will be reviewed, and if they are still deemed to be of public interest, they may be retained for longer.

How do I find out the results?

Researchers at UEA will provide a summary of the findings online through the UEA website. We will provide a hyperlink to all participants (via their email address) of this once the data has been analysed and reported.

Concerns

If you have any concerns about the study, you can contact the UEA study team at 01603 597205 or email noar@uea.ac.uk. If you feel your concerns have not been handled satisfactorily, you can contact the Dean of the Norwich Medical School (Professor William Fraser) at: W.Fraser@uea.ac.uk

Consent

By taking part, you are agreeing that you have read and understood the information above about the study. If you have any questions or concerns please contact 01603 597205 before agreeing to take part and completing the survey.

I understand that:

- My participation is completely voluntary.
- The data gathered in this study will be stored securely and it will not be possible to identify me in any outputs from this research.
- Only UEA research staff, will have access to the data.

I consent by ticking this box: ☐

Survey

Date of birth ☐☐ / ☐☐ / ☐☐☐☐

Date completed ☐☐ / ☐☐ / ☐☐☐☐

Email Address (to send the follow-up surveys) ☐

Female ☐

Male ☐

Age (years) ☐

What is was/your occupation:______________________________________

Are you currently:

a. Working now	☐	g. Homekeeper/parent/carer	☐
b. Unemployed	☐	h. Student	☐
c. Off sick	☐	i. Working reduced hours	☐
d. Retired	☐	j. Working, but there has been a change in duties	☐
e. Not working – health grounds	☐	k. Maternity/Paternity leave	☐
f. Never worked	☐		

	YES	NO
**Are you currently off work because of coronavirus self-isolation?**	☐	☐
**Are you currently working from home because of coronavirus self-isolation?**	☐	☐

For peer review only

Which category best describes your ethnic group or background. (Tick only one box)

White	☐
Black, Caribbean	☐
Black, African	☐
Any other Black background	☐
Indian	☐
Pakistani	☐
Bangladeshi	☐
Any other Asian background	☐
Chinese	☐
White and Black Caribbean	☐
White and Black African	☐
White and Asian	☐
Any other mixed background	☐
Any other ethnic group	☐

For peer review only

Your Bone, Joint and Muscle Disease

What is your musculoskeletal disease diagnosis? (Please tick all relevant boxes)

Rheumatoid Arthritis (RA)	
Osteoarthritis (OA)	
Fibromyalgia	
Ankylosing Spondylitis	
Psoriatic Arthritis (PsA)	
Inflammatory Polyarthritis (IP)	
Mechanical Low Back Pain	
Other (please specify) ________________	
Don't Know	

Where do you feel your pain?

	Left	Right
Hip		
Knee		
Ankle		
Foot		
Shoulder		
Elbow		
Wrist		
Hand		
Neck		
Back (mid or low back)		

For peer review only

General Health Questions

1. Have you had any of the following conditions?

	YES	NO
Heart condition (such as heart failure or past heart attack)		
High blood pressure		
Stroke or TIA (mini stroke/transient visual loss)		
Breathing condition (such as asthma, emphysema, COPD)		
Diabetes		
Stomach Ulcer		
Liver problems		
Kidney problems		
Cancer (except skin cancer)		
Depression or Anxiety		
Glaucoma		
Dementia		
Epilepsy		

For peer review only

<u>Changes since self-isolation</u>

Are you self-isolated for COVID-19 (i.e. not leaving home for activities other than shopping, exercise once daily or essential tasks)?

❑ Yes ❑ No

Have you had difficulty accessing medications?

❑ Yes ❑ No

Have you needed someone to help to access your medications?

❑ Yes ❑ No

Have you had to change your medications since the COVID-19 pandemic started?

❑ Yes ❑ No

Have you needed to seek advice from a health professional on your condition?

❑ Yes ❑ No

Has your normal level of physical activity changed since the COVID self isolation started?

❑ Decreased ❑ Stayed same ❑ Increased

How has your joint pain been overall since the COVID self isolation started?

❑ Decreased ❑ Stayed same ❑ Increased

How have your levels of energy been since the COVID self isolation started?

❑ Decreased ❑ Stayed same ❑ Increased

For peer review only

Clinical Health Assessment Questionnaire (CLINHAQ)

We are interested in learning how your illness affects your ability to function in daily life.

Please tick the response which best describes your usual abilities OVER THE PAST WEEK:

	Without any difficulty	With some difficulty	With much difficulty	Unable to do
DRESSING & GROOMING: Are you able to:				
Dress yourself, including tying shoelaces & doing buttons?	______	______	______	______
Shampoo your hair?	______	______	______	______
RISING: Are you able to:				
Stand up from an armless straight chair?	______	______	______	______
Get in and out of bed?	______	______	______	______
EATING: Are you able to:				
Cut your meat?	______	______	______	______
Lift a full cup or glass to your mouth?	______	______	______	______
Open a new carton of milk (or soap powder)?	______	______	______	______
WALKING: Are you able to:				
Walk outdoors on flat ground?	______	______	______	______
Climb up five steps?	______	______	______	______

Please tick any AIDS or DEVICES that you usually use for any of these activities:

______ Cane (W) ______ Walking frame (W) ______ Built up or special utensils (E)

______ Crutches (W) ______ Wheelchair (W) ______ Special or built up chair (A)

______ Devices used for dressing (button hook, zipper pull, long handled shoe horn) (D)

______ Other, specify ____

Please tick any category for which you usually need HELP FROM ANOTHER PERSON:

________ Dressing & grooming ________ Eating

________ Rising ________ Walking

We are also interested in learning whether or not you are affected by pain because of your illness.

How much pain have you had because of your arthritis IN THE PAST WEEK?
Place a mark on the line to indicate the severity of the pain

No pain Severe pain

0 10 20 30 40 50 60 70 80 90 100

How much trouble have you had with your stomach (ie nausea, heartburn, bloating, pain, etc)

IN THE PAST WEEK? Place a mark on the line below to indicate the severity of the problem:

No stomach problem Severe stomach problem

0 10 20 30 40 50 60 70 80 90 100

For peer review only

Please tick the response which best describes your usual abilities OVER THE PAST WEEK:

	Without any difficulty	With some difficulty	With much difficulty	Unable to do
HYGIENE: Are you able to:				
Wash and dry your entire body?	_______	_______	_______	_______
Take a bath?	_______	_______	_______	_______
Get on and off the toilet?	_______	_______	_______	_______
REACH: Are you able to:				
Reach and get down a 5lb object (eg a bag of potatoes) from just above your head?	_______	_______	_______	_______
Bend down to pick up clothing from the floor?	_______	_______	_______	_______
GRIP: Are you able to:				
Open car doors?	_______	_______	_______	_______
Open jars which have been previously opened?	_______	_______	_______	_______
Turn taps on and off?	_______	_______	_______	_______
ACTIVITIES: Are you able to:				
Run errands and shop?	_______	_______	_______	_______
Get in and out of a car?	_______	_______	_______	_______
Do chores (vacuuming, housework or light gardening)?	_______	_______	_______	_______

Please tick any AIDS OR DEVICES that you usually use for any of these activities:

________ Bath rail (H) ________ Bath seat (H)

________ Raised toilet seat (H) ________ Jar opener (for jars previously opened) (G)

________ Long-handled appliances for reach (R) ________ Other (specify) ______________________

Please tick any categories for which you usually need HELP FROM ANOTHER PERSON:

________ Hygiene ________ Gripping and opening things

________ Reach ________ Errands and chores

In general, would you say that your HEALTH IS:

________ Excellent ________ Good ________ Fair ________Poor

Consider ALL THE WAYS THAT YOUR ARTHRITIS AFFECTS YOU. RATE HOW YOU ARE DOING on the following scale by placing a mark on the line below:

Very well Very poorly

0 10 20 30 40 50 60 70 80 90 100

We are interested in knowing about any problems that you may have been having with fatigue.

How much of a problem has fatigue or tiredness been for you IN THE PAST WEEK? Place a mark on the line below:

Fatigue is no problem Fatigue is a major problem

0 10 20 30 40 50 60 70 80 90 100

For peer review only

How much of a problem has sleep (ie resting at night) been for you IN THE PAST WEEK? Place a mark on the line below:

Sleep is no problem Sleep is a major problem

0 10 20 30 40 50 60 70 80 90 100

For peer review only

Lubben Social Network Scale-6

The following 6 questions are about how often you are in contact with your family and friends. Please **tick** the box to best represent your answer for each question.

	None	1	2	3 or 4	5 to 8	9 or more
Family – considering the people to whom you are related by birth, marriage, adoption etc…						
How many relatives do you see or hear from at least once a month?						
How many relatives do you feel at ease with that you can talk about private matters?						
How many relatives do you feel close to such that you could call on them for help?						
Considering all of your friends, including those who live in your neighbourhood						
How many of your friends do you see or hear from at least once a month?						
How many friends do you feel at ease with that you can talk about private matters?						
How many friends do you feel close to such that you could call on them for help?						

University of California, Los Angeles (UCLA) three-item loneliness scale

The following 3 questions are about how often you feel lonely. Please **tick** the box to best represent your answer for each question.

	Hardly Ever	Some of the Time	Often
How often do you feel that you lack companionship?			
How often do you feel left out?			
How often do you feel isolated from others?			

6-item Brief Resilience Scale

The following 6 statement relate to how 'resilient' you feel. Please respond to each item by marking one box per row.

	Strongly Disagree	Disagree	Neutral	Agree	Strongly Agree

I tend to bounce back quickly after hard times					
I have a hard time making it through stressful events					
It does not take me long to recover from a stressful events					
It is hard for me to snap back when something wrong happens					
I usually come through difficult times with little trouble					
I tend to take a long time to get over setbacks in my life					

Revised Life Orientation Test (LOT-R)

Please answer the following questions about yourself. Be as honest as you can throughout and try not to let your responses to one question influence your response to other questions. There are no right or wrong answers.

	Strongly Disagree	Disagree	Neutral	Agree	Strongly Agree
In uncertain times, I usually expect the best					
It's easy for me to relax					
If something can go wrong for me, it will					
I'm always optimistic about my future					
I enjoy my friends a lot					
It's important for me to keep busy					
I hardly ever expect things to go my way					
I don't get upset too easily					
I rarely count on good things happening to me					
Overall, I expect more good things to happen to me than bad					

We would like to thank you for completing these questionnaires.
Your contribution is much appreciated.
If you have any questions or queries about this form, please contact:
NOAR Office Tel: (01603) 597204/5
Email: noar@uea.ac.uk

For peer review only

Supplementary File 2: Timeline of study activity against UK legislation and COVID policy implementation.

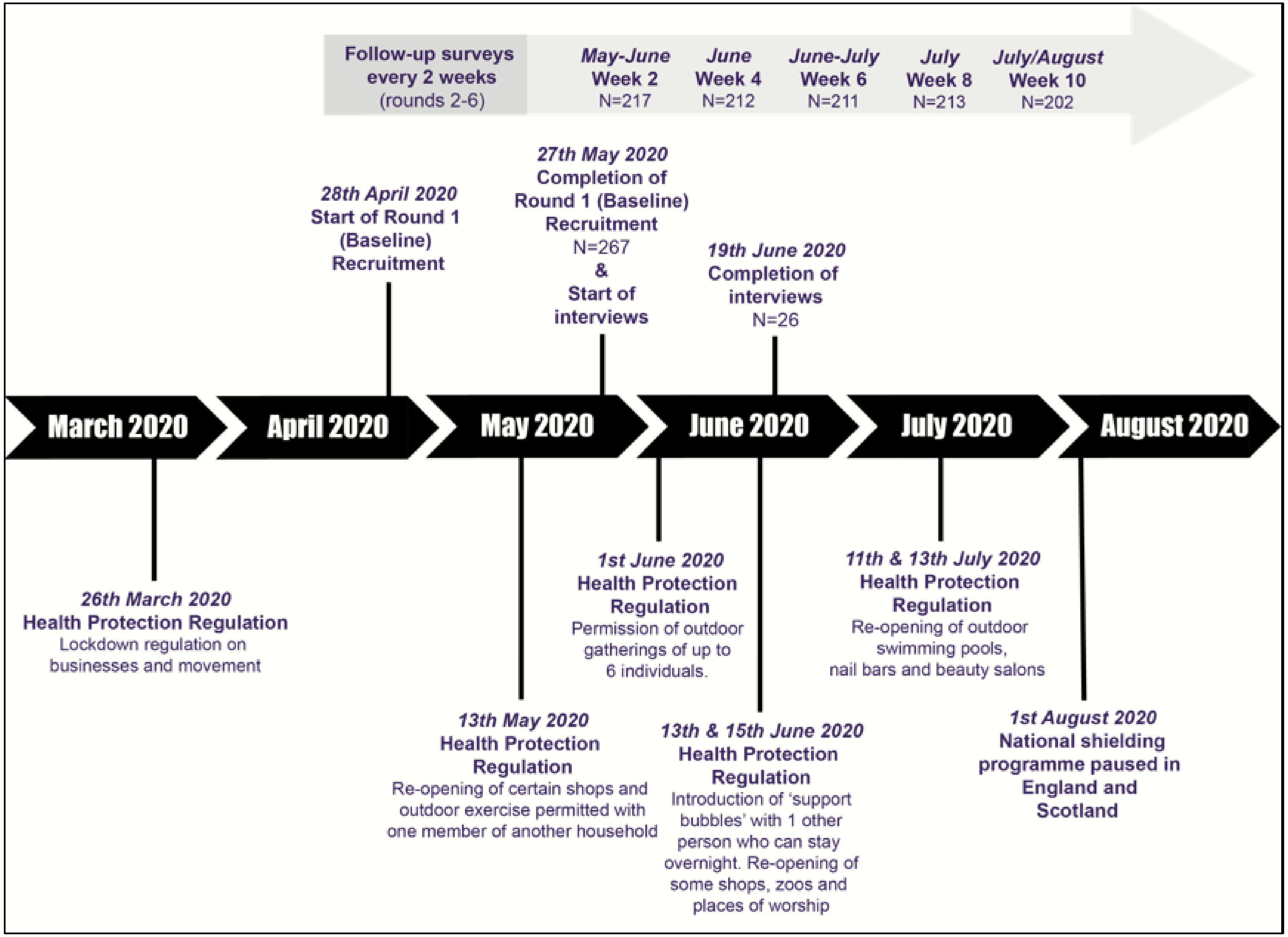

Supplementary File 3: Interview topic guide

1 **How is everyday life for you at the moment?**

Prompts:

- Getting out the house
- Keeping in touch with people
- Changes in routines
- Changes in mood
- Changes in health
- Any adaptions made

2 **How is your joint pain?**

Prompts

- Recent changes and what they think is the cause
- Fatigue
- Medication
- Exercise
- Self help
- Contact with health professionals

3 **How are you managing daily tasks?**

Prompts

- Do you usually have help shopping, cleaning
- Have you made changes to the ways you do things

3a How do you feel about these changes?

4 **How have your social activities changed?**

Prompts

- Keeping in touch with family and friends
- Activities in the community i.e. clubs, quizzes crafts, pub
- Using social media
- Accessing support groups
- New social contacts

4a How do you feel about these changes?

5 **Is there anything else you would like to tell us about your life during this period?**

For peer review only

Supplementary File 4: Characteristics of participants at baseline and Week 10

	Timepoint		P-value (chi-squared)
	Baseline	Week 10	
N (% of baseline)	703 (100)	490 (70)	
**Age group**			p=0.022
18-60	351 (50)	212 (43)	
60 plus	351 (50)	278 (57)	
**Gender**			p=0.804
Male	574 (82)	402 (83)	
Female	126 (18)	85 (17)	
**MSK Diagnosis**			p=0.632
RA	321 (46)	241 (48)	
IA	41 (6)	29 (6)	
PsA	65 (9)	48 (9)	
Other	269 (39)	171 (37)	

Data are frequency (%); MSK – Musculoskeletal; RA - Rheumatoid arthritis, IA - Inflammatory arthritis; PsA - Psoriatic arthritis; Other - mainly Osteoarthritis (55%) but also including any diagnosis that was not RA, IA or PsA (see **Table 1**)

For peer review only

Supplementary File 5: Interpretive synthesis of quantitative and qualitative findings

Survey Findings (See Tables 1-2)	Interview Findings (See Tables 3-4)
People with musculoskeletal disease reported an increase in pain and symptoms	*Attributions from symptom increase:*<br>- Changes in access to healthcare<br>- Reduced physical activity<br>- Ergonimics of working at home<br>- Reduced wellbeing<br>- Increased demands at home
Those who reported decreased physical activity were more likely to report increased symptoms	*Increase in symptoms attributed to:*<br>- Reduced routine activitiy<br>- Reduced opportunities for exercise
Increase in symptoms was significantly worse for those aged 18-60 years of age in comparison to older respondents	*Changes to daily life described by working age people included:*<br>- Disrupted daily routines<br>- Managing multiple roles at home, including childcare<br>- Unsuitable home working environment
Over the 10 weeks, greater social isolation, loneliness and reduced optimism were reported	*Feelings of low mood, boredom, social isolation or anxiety were related to:*<br>- Experience of restrictive measures<br>- Being in a clinically vulnerable group<br>- Disease symptoms<br>- A sense of being forgotten about
Half of those (53%) with increased symptoms at baseline had not consulted a healthcare professional	*Disrupted access to healthcare:*<br>- 'Holding on to concerns', wary of burdening healthcare staff, or uncertainty around service availability<br>- Reservations about telephone consultations<br>- Appointments, treatments and investigations postponed or cancelled

STROBE Statement—checklist of items that should be included in reports of observational studies

	Item No	Recommendation	Page No
**Title and abstract**	1	(*a*) Indicate the study's design with a commonly used term in the title or the abstract	Title Page
		(*b*) Provide in the abstract an informative and balanced summary of what was done and what was found	Abstract
**Introduction**			
Background/rationale	2	Explain the scientific background and rationale for the investigation being reported	Intro Para 1-3
Objectives	3	State specific objectives, including any prespecified hypotheses	Intro Para 4
**Methods**			
Study design	4	Present key elements of study design early in the paper	Methods Para 1
Setting	5	Describe the setting, locations, and relevant dates, including periods of recruitment, exposure, follow-up, and data collection	Methods Para 2-4, 9
Participants	6	(*a*) *Cohort study*—Give the eligibility criteria, and the sources and methods of selection of participants. Describe methods of follow-up<br>*Case-control study*—Give the eligibility criteria, and the sources and methods of case ascertainment and control selection. Give the rationale for the choice of cases and controls<br>*Cross-sectional study*—Give the eligibility criteria, and the sources and methods of selection of participants	Methods, Para 2, 8
		(*b*) *Cohort study*—For matched studies, give matching criteria and number of exposed and unexposed<br>*Case-control study*—For matched studies, give matching criteria and the number of controls per case	Not applicable
Variables	7	Clearly define all outcomes, exposures, predictors, potential confounders, and effect modifiers. Give diagnostic criteria, if applicable	Methods Para 4,6
Data sources/ measurement	8*	For each variable of interest, give sources of data and details of methods of assessment (measurement). Describe comparability of assessment methods if there is more than one group	Methods Para 4
Bias	9	Describe any efforts to address potential sources of bias	Discussion Para 5,6
Study size	10	Explain how the study size was arrived at	Methods, Para 3, 6, 10
Quantitative variables	11	Explain how quantitative variables were handled in the analyses. If applicable, describe which groupings were chosen and why	Methods, Para 7, 8
Statistical methods	12	(*a*) Describe all statistical methods, including those used to control for confounding	Methods, Para 7, 8
		(*b*) Describe any methods used to examine subgroups and interactions	Methods, Para 8
		(*c*) Explain how missing data were addressed	Methods Para 8
		(*d*) *Cohort study*—If applicable, explain how loss to follow-up was addressed	Not applicable

For peer review only - http://bmjopen.bmj.com/site/about/guidelines.xhtml

For peer review only

		Case-control study—If applicable, explain how matching of cases and controls was addressed<br>*Cross-sectional study*—If applicable, describe analytical methods taking account of sampling strategy	
		(*e*) Describe any sensitivity analyses	Methods Para 8
**Results**			
Participants	13*	(a) Report numbers of individuals at each stage of study—eg numbers potentially eligible, examined for eligibility, confirmed eligible, included in the study, completing follow-up, and analysed	Results, Para 1
		(b) Give reasons for non-participation at each stage	Results, Para 1
		(c) Consider use of a flow diagram	Considered but not prioritised
Descriptive data	14*	(a) Give characteristics of study participants (eg demographic, clinical, social) and information on exposures and potential confounders	Results, Para 1, Table 1
		(b) Indicate number of participants with missing data for each variable of interest	Results, Para 1, Table 1-3
		(c) *Cohort study*—Summarise follow-up time (eg, average and total amount)	Methods, Para 6
Outcome data	15*	*Cohort study*—Report numbers of outcome events or summary measures over time	Results, Para 1, Table 1-3
		Case-control study—Report numbers in each exposure category, or summary measures of exposure	No CC study
		Cross-sectional study—Report numbers of outcome events or summary measures	Not cross-sectional
Main results	16	(*a*) Give unadjusted estimates and, if applicable, confounder-adjusted estimates and their precision (eg, 95% confidence interval). Make clear which confounders were adjusted for and why they were included	Table 2
		(*b*) Report category boundaries when continuous variables were categorized	Methods, Para 8
		(*c*) If relevant, consider translating estimates of relative risk into absolute risk for a meaningful time period	Not applicable
Other analyses	17	Report other analyses done—eg analyses of subgroups and interactions, and sensitivity analyses	Results, Table 3
**Discussion**			
Key results	18	Summarise key results with reference to study objectives	Discussion Para 1
Limitations	19	Discuss limitations of the study, taking into account sources of potential bias or imprecision. Discuss both direction and magnitude of any potential bias	Discussion Para 5,6
Interpretation	20	Give a cautious overall interpretation of results considering objectives, limitations, multiplicity of analyses, results from similar studies, and other relevant evidence	Conclusion Para 1

For peer review only

Generalisability	21	Discuss the generalisability (external validity) of the study results	Discussion Para 5,6
**Other information**			
Funding	22	Give the source of funding and the role of the funders for the present study and, if applicable, for the original study on which the present article is based	Declarations

*Give information separately for cases and controls in case-control studies and, if applicable, for exposed and unexposed groups in cohort and cross-sectional studies.

Note: An Explanation and Elaboration article discusses each checklist item and gives methodological background and published examples of transparent reporting. The STROBE checklist is best used in conjunction with this article (freely available on the Web sites of PLoS Medicine at http://www.plosmedicine.org/, Annals of Internal Medicine at http://www.annals.org/, and Epidemiology at http://www.epidem.com/). Information on the STROBE Initiative is available at www.strobe-statement.org.

For peer review only

COREQ (COnsolidated criteria for REporting Qualitative research) Checklist

A checklist of items that should be included in reports of qualitative research. You must report the page number in your manuscript where you consider each of the items listed in this checklist. If you have not included this information, either revise your manuscript accordingly before submitting or note N/A.

Topic	Item No.	Guide Questions/Description	Reported on Page No.
**Domain 1: Research team and reflexivity**			
Personal characteristics			
Interviewer/facilitator	1	Which author/s conducted the interview or focus group?	
Credentials	2	What were the researcher's credentials? E.g. PhD, MD	
Occupation	3	What was their occupation at the time of the study?	
Gender	4	Was the researcher male or female?	
Experience and training	5	What experience or training did the researcher have?	
Relationship with participants			
Relationship established	6	Was a relationship established prior to study commencement?	
Participant knowledge of the interviewer	7	What did the participants know about the researcher? e.g. personal goals, reasons for doing the research	
Interviewer characteristics	8	What characteristics were reported about the inter viewer/facilitator? e.g. Bias, assumptions, reasons and interests in the research topic	
**Domain 2: Study design**			
Theoretical framework			
Methodological orientation and Theory	9	What methodological orientation was stated to underpin the study? e.g. grounded theory, discourse analysis, ethnography, phenomenology, content analysis	
Participant selection			
Sampling	10	How were participants selected? e.g. purposive, convenience, consecutive, snowball	
Method of approach	11	How were participants approached? e.g. face-to-face, telephone, mail, email	
Sample size	12	How many participants were in the study?	
Non-participation	13	How many people refused to participate or dropped out? Reasons?	
Setting			
Setting of data collection	14	Where was the data collected? e.g. home, clinic, workplace	
Presence of non-participants	15	Was anyone else present besides the participants and researchers?	
Description of sample	16	What are the important characteristics of the sample? e.g. demographic data, date	
Data collection			
Interview guide	17	Were questions, prompts, guides provided by the authors? Was it pilot tested?	
Repeat interviews	18	Were repeat inter views carried out? If yes, how many?	
Audio/visual recording	19	Did the research use audio or visual recording to collect the data?	
Field notes	20	Were field notes made during and/or after the inter view or focus group?	
Duration	21	What was the duration of the inter views or focus group?	
Data saturation	22	Was data saturation discussed?	
Transcripts returned	23	Were transcripts returned to participants for comment and/or	

For peer review only

Topic	Item No.	Guide Questions/Description	Reported on Page No.
		correction?	
**Domain 3: analysis and findings**			
Data analysis			
Number of data coders	24	How many data coders coded the data?	
Description of the coding tree	25	Did authors provide a description of the coding tree?	
Derivation of themes	26	Were themes identified in advance or derived from the data?	
Software	27	What software, if applicable, was used to manage the data?	
Participant checking	28	Did participants provide feedback on the findings?	
Reporting			
Quotations presented	29	Were participant quotations presented to illustrate the themes/findings? Was each quotation identified? e.g. participant number	
Data and findings consistent	30	Was there consistency between the data presented and the findings?	
Clarity of major themes	31	Were major themes clearly presented in the findings?	
Clarity of minor themes	32	Is there a description of diverse cases or discussion of minor themes?	

Developed from: Tong A, Sainsbury P, Craig J. Consolidated criteria for reporting qualitative research (COREQ): a 32-item checklist for interviews and focus groups. *International Journal for Quality in Health Care*. 2007. Volume 19, Number 6: pp. 349 – 357

Once you have completed this checklist, please save a copy and upload it as part of your submission. DO NOT include this checklist as part of the main manuscript document. It must be uploaded as a separate file.

For peer review only
